# Alternative splicing-induced thioesterase domain deficiency drives the production of natural orsellinic acid derivatives with a pantetheine moiety in nonreducing polyketide synthases

**DOI:** 10.1016/j.synbio.2026.07.008

**Published:** 2026-08-07

**Authors:** Qingpei Liu, Liwei Zhou, Yifu Gong, Xinqiong Liu, Jiajie Ma, Wei Liu, Haiyan Fu, Xiaolong Yang

**Affiliations:** a School of Pharmaceutical Sciences, South-Central Minzu University, Wuhan, 430074, PR China; b Anhui Province Key Laboratory of Bioactive Natural Products, School of Pharmacy, Anhui University of Chinese Medicine, Hefei, 230012, PR China; c College of Life Science, South-Central Minzu University, Wuhan, 430074, PR China

**Keywords:** Polyketide synthases, Alternative splicing, Natural products, Orsellinic acid derivatives, Pantetheine, Thioesterase domain

## Abstract

Nonreducing polyketide synthase Preu6 catalyzes didepside formation by interaction of starter acyl transferase and thioesterase (TE) domains. Here, we show that an aberrant transcript, *preu6β*, is generated to encode the TE-deficient protein *via* alternative splicing in A5SS pattern. Upon heterologous expression in *Saccharomyces cerevisiae* BJ5464-NpgA, coupled with *in vitro* chemical and enzymatic assays, Preu6*β* affords compound **9**, the first natural orsellinic acid (OA) derivative with a pantetheine (PANT) moiety. We also demonstrate that TE inactivation represents a previously unrecognized mechanism that governs the selective formation of OA derived-PANT products.

## Graphical abstract

**Figure:**
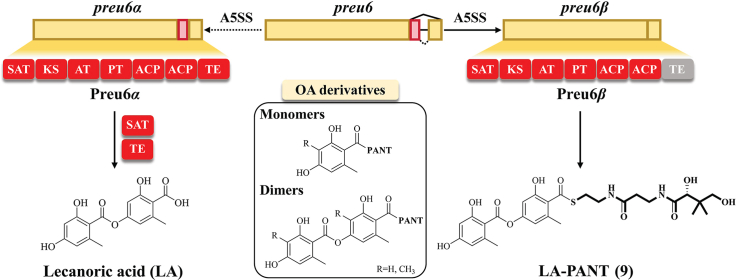

Orsellinic acid (OA) derivatives produced by filamentous fungi are a classic class of natural polyketides [1]. Beyond monomers, they predominantly form polymers such as depsides, depsidones, and diphenyl ethers (DPEs) *via* ester or ether linkages, which display intriguing structural diversity and significant biological activities [[2], [3], [4]]. Over the past five years, the underlying mechanism for ester linkage formation in depsides, catalyzed by the nonreducing polyketide synthases (nrPKSs), has attracted considerable scientific interest, leading to the elucidation of two principal catalytic strategies (Fig. S1) [5]. One involves the thioesterase (TE) domain-catalyzed esterification, exemplified by the multifunctional TE domain first reported in AN7909, which integrates a series of successive steps including chain transfer, esterification, and hydrolysis [6]; while TE domain could also be responsible for two sequential rounds of esterification reactions in the synthesis of tridepsides, as demonstrated in thielavin A [7]. The second one is the starter acyl transferase (SAT) domain-catalyzed ester bond formation with only two experimentally confirmed examples to date: DrcA and Preu6 (also known as DpeA), whose SAT domains exhibit high structural similarity with template modeling score (TM-score) of 0.9471 (Fig. S2) [[8], [9], [10], [11]]. Notably, Preu6 is a novel nrPKS characterized from *Preussia isomera* XL-1326, as we previously reported, in which we elucidated that it catalyzes the efficient synthesis of lecanoric acid (LA) through a specific physical interaction between the SAT and TE domains *via* a combination of domain swaps, domain dissections and reconstitutions, and yeast two-hybrid assays in *Saccharomyces cerevisiae* [9].

Interestingly, a subsequent analysis of the transcripts of *preu6* gene demonstrated that there exist two types of spliceosomes: *preu6α* and *preu6β* (Fig. S3). *Preu6α* retains the first part of intron 3 (i_3_-1) (70 bp), thereby encoding the nrPKS Preu6*α*, harboring a full set of modules: an N-terminal loading module (SAT domain), a central chain elongation module (ketoacyl synthase [KS], acyl transferase [AT], acyl carrier protein [ACP], and product template [PT] domains), and a C-terminal processing module (TE domain) (Table S1) [12]. In contrast, the removal of i_3_-1 in *preu6β* induced a frameshift, and introduced a premature termination codon to encode the TE domain-deficient Preu6*β*. The splicing pattern of *preu6α/β* corresponds to a basic type of alternative splicing (AS), alternative 5′ splice site (A5SS) (Fig. 1A) [13]. The overall temporal expression of *preu6α/β* across XL-1326 from 4 to 14 days revealed that the expression of *preu6α* increased from day 6, reached a maximum at day 10, and subsequently declined, while the aberrant mRNA *preu6β* represented a relatively stable expression (Fig. 1B). Samples were further treated with an inhibitor of the nonsense-mediated mRNA decay (NMD) pathway, then monitored the expression dynamics of the two spliceosomes by specific days 6, 10 and 14 [14]. The abundance of *preu6β* increased markedly, especially at day 6 and day 10, whereas the expression levels of *preu6α* remained largely unaltered, indicating that NMD pathway is actively involved in the post-transcriptional regulation of the aberrant mRNA *preu6β*, to possibly avoid the generation of excessive truncated proteins in XL-1326 cells (Fig. 1B) [15].

**Fig. 1:**
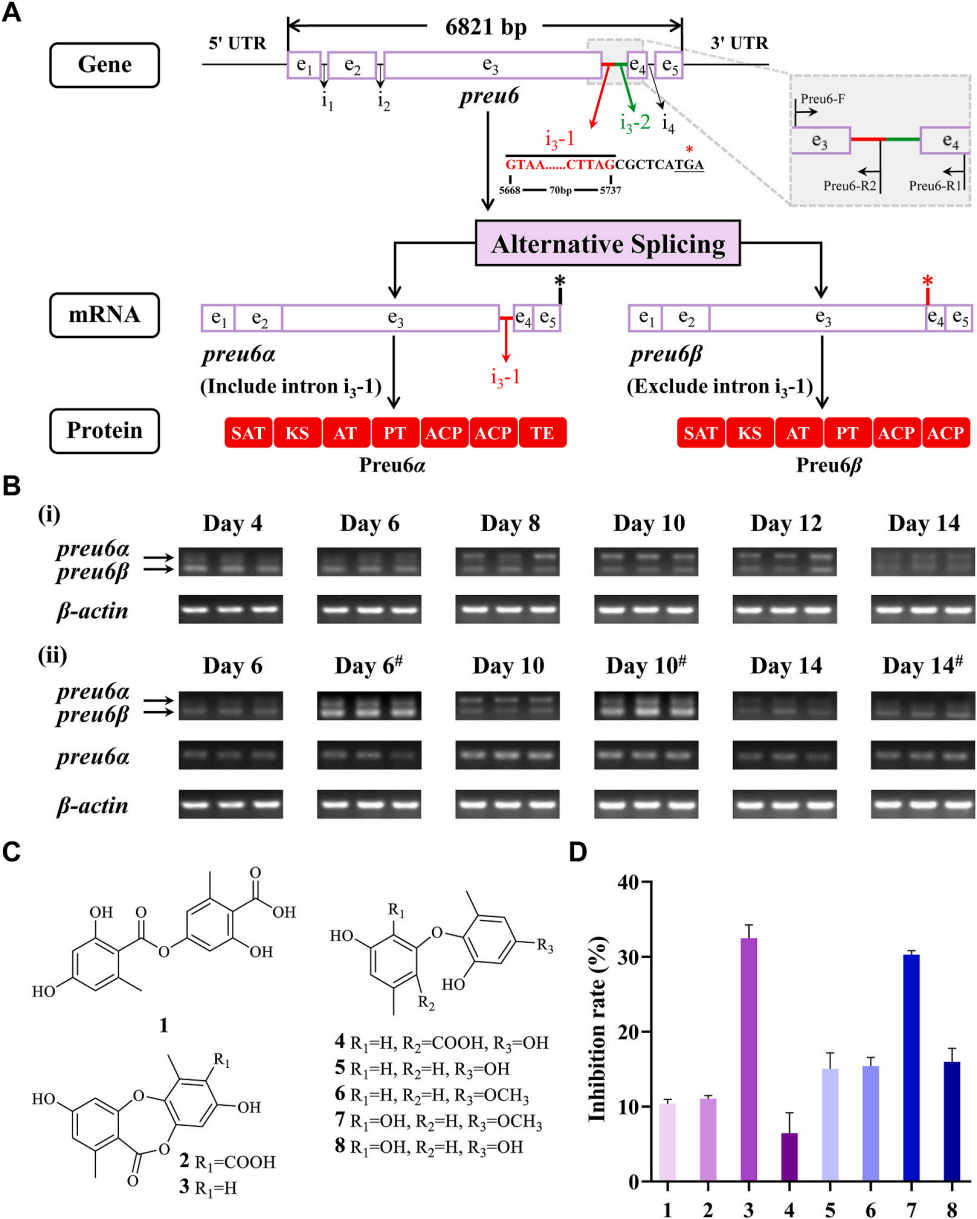
Alternative splicing (AS) event in *preu6* gene. A) Schematic representation of the AS sites and the corresponding AS mode. *, the termination codon. B) RT-PCR analysis of the transcripts *preu6α/β* in the native organism *P. isomera* XL-1326 cultivated on PDA plates for (i) 4, 6, 8, 10, 12, 14 days; and (ii) 6, 10, 14 days at 28°C. ***#*** indicates the presence of 5 μg/mL cycloheximide in PDA medium. C) Structures of compounds **1**–**8** by the *dpe* gene cluster. D) Inhibition rates of compounds **1**–**8** against XL-1326.

Harboring Preu6*α*, the *dpe* gene cluster performed iterative structural transformations from “depsides to depsidones to DPEs” *via* a multistep and multi-enzyme cascade, yielding compounds **1**–**8**, which exemplifies a novel biosynthetic paradigm for the assembly of DPEs (Fig. 1C and S4) [10]. Notably, compounds **1**–**8** displayed varying levels of inhibitory activities against the wildtype strain XL-1326, especially compounds **3** and **7**, whose inhibition rates exceeded 30% (Fig. 1D); moreover, **3** and **7** also showed moderate antifungal efficacy against three agricultural pathogenic fungi, *Botryosphaeria berengeriana*, *Helminthosporium maydis*, and *Verticillium dahliae* Kleb with minimum inhibitory concentrations (MIC) of 25–50 μg/mL (Table S2). Thus, we propose that the wildtype strain XL-1326 may balance environmental fitness with self-protection through AS of the PKS gene *preu6* to modulate the production of *dpe* cluster-encoded metabolites. An analogous regulatory mechanism has been described in *Shiraia bambusicola*, where the AS of the key PKS gene *sbPKS* controls the biosynthetic output of the hypocrellin as part of the defense against environmental stresses [16].

*ASpks1*, a nrPKS gene encoding 3-methylorcinaldehyde synthase during the xenovulene A biosynthesis in *Acremonium strictum*, undergoes the AS event to yield transcript encoding the reductive release (R) domain-deficient PKS, which biosynthesized 3-methylorsellinic acid (3-MOA) instead in *Aspergillus oryzae* (Fig. S5) [17]. Here, upon heterologous expression in *S. cerevisiae* BJ5464-NpgA, TE-deficient Preu6*β* afforded a set of new metabolites (compounds **9**–**11**), instead of releasing LA (**1**), which is generated by the full protein Preu6*α*. Correspondingly, the Preu6*α*(H2177A), with the histidine^2177^ residue of the catalytic triad in TE domain mutating to alanine, reproduced this product profile (Fig. 2A) [9,18]. Compounds **10** (*p*-hydroxyphenylethyl orsellinate, new compound) and **11** (ethyl orsellinate) may be released utilizing *p*-hydroxyphenylethanol and ethanol as nucleophiles, respectively, which are either present in the media or endogenously produced by *S. cerevisiae* (Fig. 2B and C) [19,20]. Notably, compound **9** (**LA-PANT**) comprises the LA core connected with a pantetheine (PANT) moiety *via* a thioester bond, representing the first isolated natural PANT product, whose configuration at C-15 was further supported by CD analysis and ECD calculations (Fig. S6). We hypothesized that **9** might be released by spontaneous hydrolysis of the phosphoester bond (a) in ACP-bound LA, in a manner analogous to the previously observed low-level spontaneous release of OA [9]. Alternatively, we considered that yeast might harbor an isoenzyme of the ACP phosphodiesterase (AcpH, GenBank accession: P21515.2), which was discovered in *Escherichia coli* and could remove the LA-phosphopantethine (LA-PPANT) from the serine residue of ACP domain by hydrolyzing the phosphoester bond (b) [21]. A subsequent hydrolysis of the additional phosphate group would happen to yield compound **9** (Fig. 2C). Unfortunately, no homologues of AcpH were mined in the genome of *S. cerevisiae*, suggesting that an elusive AcpH-like isoenzyme needs to be further investigated and identified. In addition, *in vitro* chemical and enzymatic assays using crude protein extracts from the *S. cerevisiae* BJ5464-NpgA expression system further confirmed that Preu6*β* is responsible for the formation of compound **9** (Fig. S7).

**Fig. 2:**
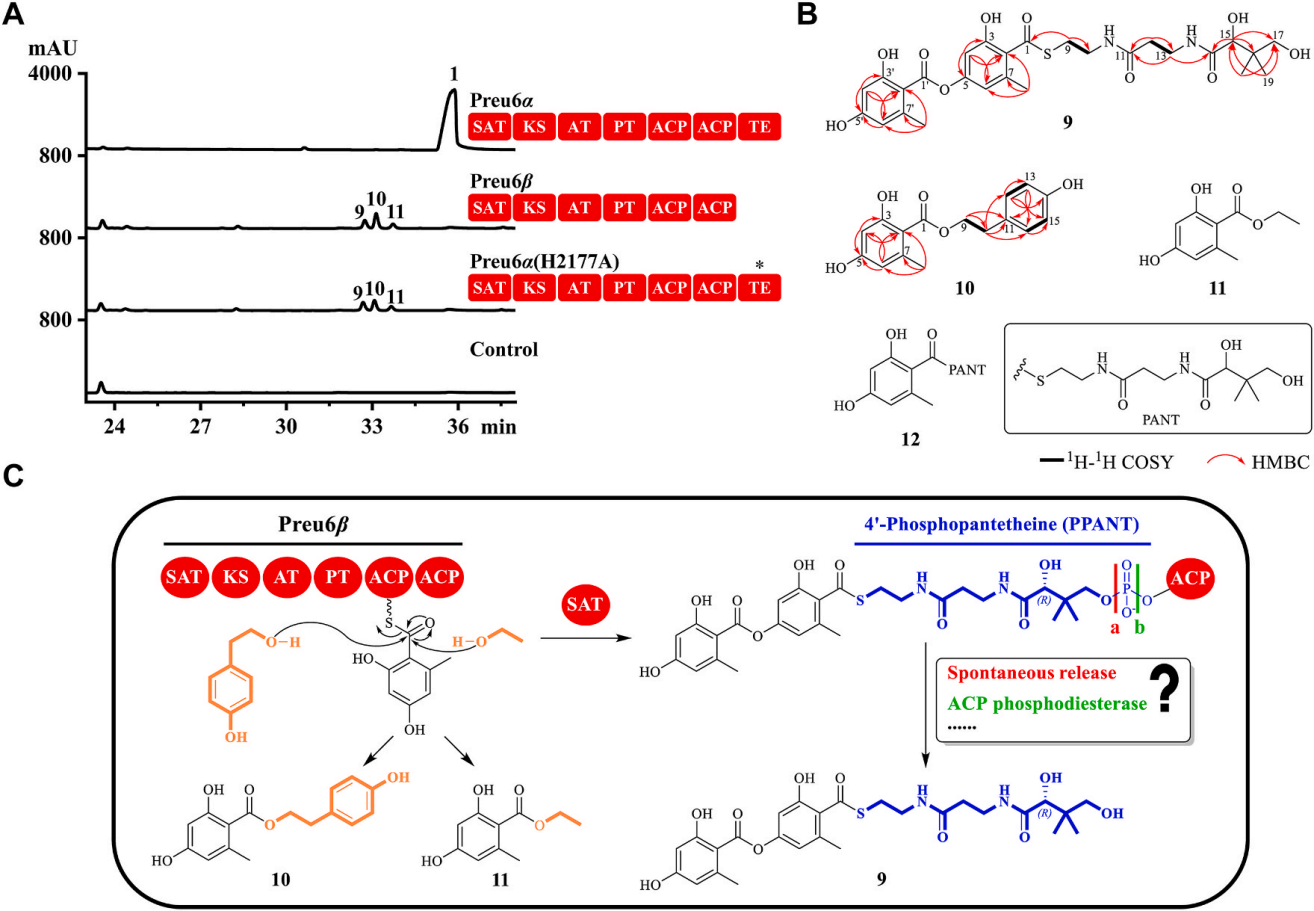
Biosynthesis of **LA-PANT** (**9**) with TE-inactivated Preu6. A) Product profiles (λ = 300 nm) of *S. cerevisiae* BJ5464-NpgA transformants expressing the indicated nrPKSs. Control, vector YEpADH2p-FLAG-URA. B) Structures of the OA derivatives, and key 2D correlations of new natural product **9** and new compound **10**. C) Proposed biosynthetic models for the OA derivatives **9**–**11**. *, the mutation site.

In contrast to Preu6*α*, TE-deficient Preu6*β* and TE-mutated Preu6*α*(H2177A) are competent to biosynthesize PANT product **9**, suggesting that TE domain inactivation might be a critical determinant for the formation of PANT metabolites. To test this hypothesis, we constructed the targeted deletions of TE/methyltransferase domain (MT)-TE, or mutations in TE of the representative nrPKSs involved in two distinct modes for ester bond formation (AN7909 and DrcA) as well as the monomeric 3-MOA synthesis (Preu3) (Fig. S1 and S2) [22]. AN7909-ΔTE could not produce compound **9**, which further proved the function of its TE domain in catalyzing esterification [6]; without TE, the truncated protein could not accumulate the corresponding ACP-bound LA. However, AN7909-ΔTE also failed to generate compound **12** (**OA-PANT**), likely reflecting the intrinsic low yield toward ACP-tethered OA in this system (Fig. 3A). In contrast, DrcA-Δ(MT + TE) with SAT-catalyzed ester bond formation phenocopied Preu6*β* by producing both **9** and **12** [8]. Interestingly, TE-mutated DrcA(H2663A) yielded a spectrum of PANT products derived from OA monomers and dimers with different methylation levels (Fig. S8). A parallel result was observed for Preu3-Δ(MT + TE) produced compound **12**, and the Preu3(H2496A) was capable of generating PANT product derived from 3-MOA (Fig. 3A and S8). Taken together, TE domain inactivation is a prerequisite for the biosynthesis of OA sourced-PANT products; meanwhile, OA derivatives with different methylation patterns are competent precursors for assembling their corresponding PANT products.

**Fig. 3:**
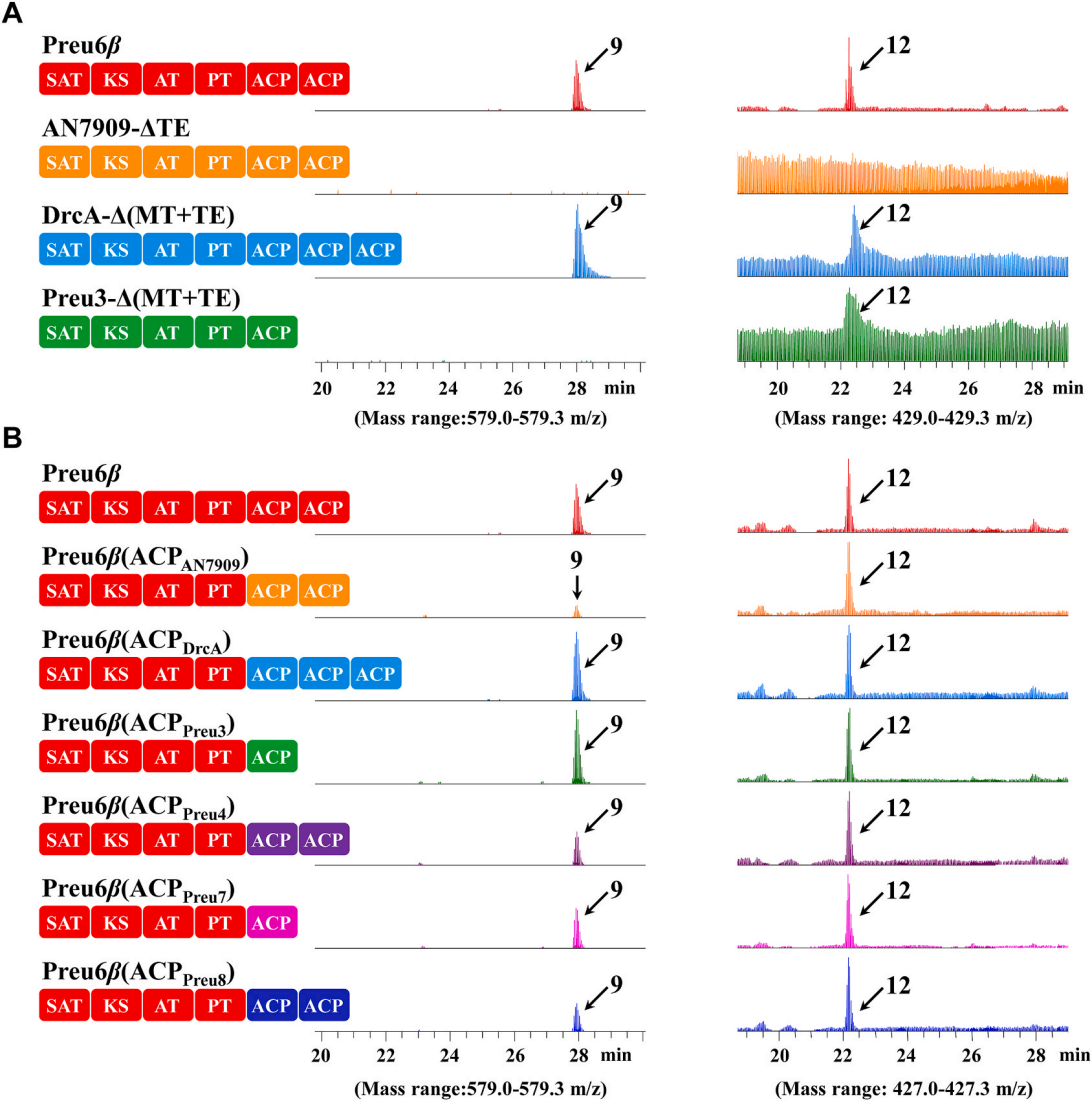
Characterization of the crucial factors for the OA derived-PANT product formation. LC-MS analyses of the products from *S. cerevisiae* BJ5464-NpgA strains expressing the indicated nrPKSs related to the targeted domain deletions A) and ACP domain replacements B).

The PANT moiety present in these OA originated-PANT products is most likely derived from the PPANT arm tethered to the ACP domain, hence this domain itself might influence the formation of the PANT products. At first, the tandem ACPs in Preu6*β* were inactivated individually or simultaneously, revealing that single ACP inactivation only reduced the yield of PANT products (Fig. S9). To further investigate the heterologous ACPs, we systematically replaced the tandem ACP domains of Preu6*β* with those originated from various nrPKSs, including ones involved in the biosynthesis of the OA-sourced dimers (AN7909 and DrcA) and monomer (Preu3), even ones also from *P. isomera* XL-1326 but responsible for the formation of polyketides structurally unrelated to OA derivatives such as naphthalene pyrone (Preu4), anthracenone (Preu7), and isocoumarins (Preu8) [[22], [23], [24], [25]]. Notably, all resulting chimeric nrPKSs retained the ability to produce compounds **9** and **12**, which indicates that the ACP domains, whether derived from PKSs related to the OA similar or disparate products, do not affect the formation of the OA derived-PANT products (Fig. 3B).

Furthermore, we investigated whether the polyketides with distinct structural scaffolds from OA-derivatives were capable of generating the corresponding PANT products. To this end, we constructed the *S. cerevisiae* transformants harboring the TE-mutated Preu4, Preu7, and other TE-mutated nrPKSs together with their cognate hrPKSs (LtLasS1-LtLasS2, AtCurS1-AtCurS2), involved in the biosynthesis of naphthalene pyrone, anthracenone, and benzenediol lactones, including resorcylic acid lactone featuring C2–C7 bridge (desmethyl-lasiodiplodin) and dihydroxyphenylacetic acid lactone with C3–C8 linkage (dehydrocurvularin), respectively [[22], [23], [24],^26^,^27^]. However, no corresponding PANT products derived from these polyketide classes were detected by LC-MS (Fig. 4), suggesting that TE domain inactivation might drive the formation of PANT products for specific structural features of OA derivatives.

**Fig. 4:**
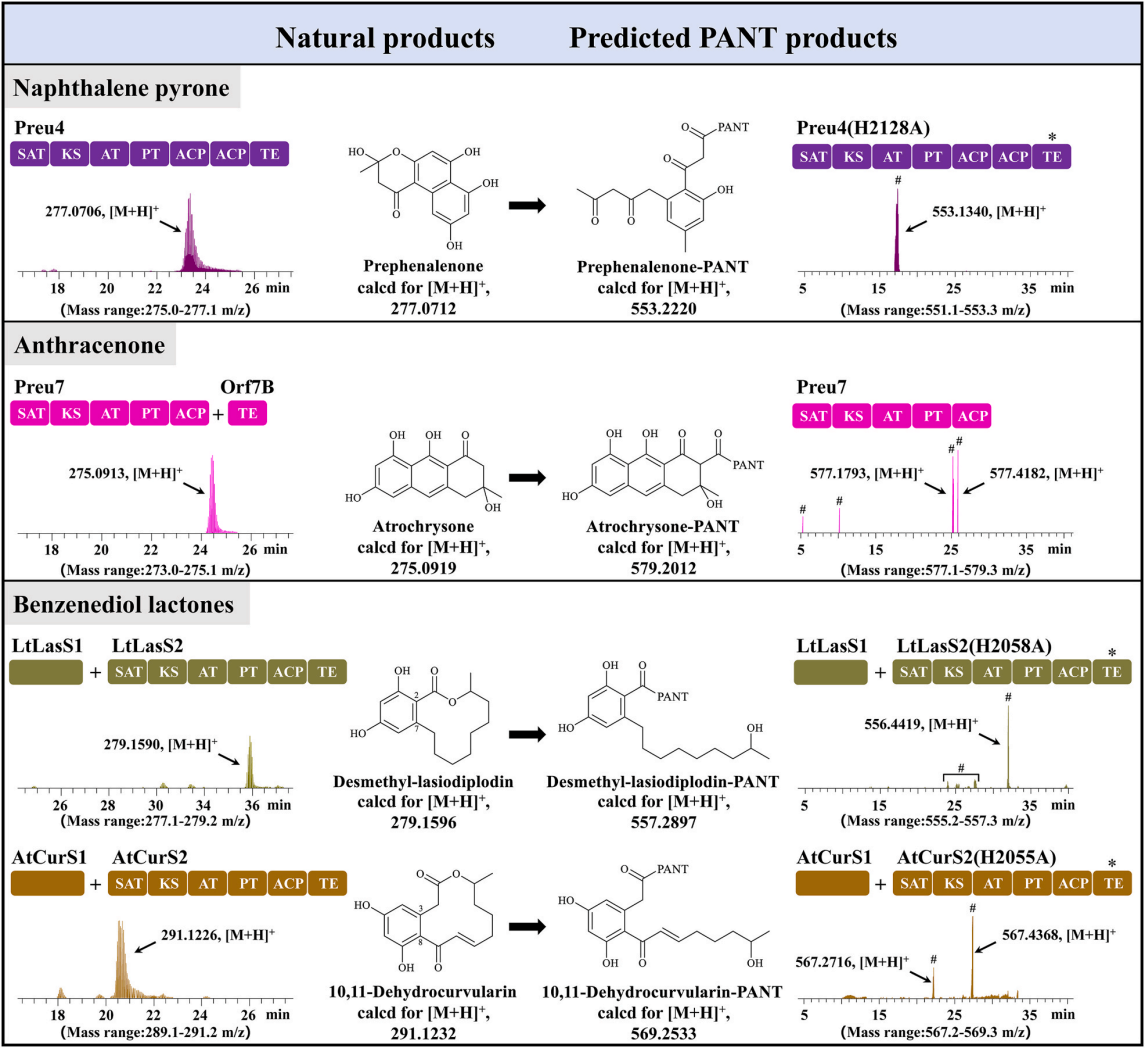
Searching for the corresponding PANT products in *S. cerevisiae* BJ5464-NpgA transformants expressed the TE-inactivated nrPKSs solely or together with the cognate hrPKSs originally synthesized naphthalene pyrone, anthracenone, and benzenediol lactones, respectively. *, the mutation sites. ***#*** indicates these peaks are not the expected PANT products.

In summary, we demonstrated that Preu6, the novel nrPKS identified from *P. isomera* XL-1326, not only catalyzed the formation of didepside through specific interaction of SAT and TE domains but also underwent the AS event in A5SS pattern during gene transcription, thereby generating a TE domain-deficient truncated protein, Preu6*β*. A comparable phenomenon has been reported for the PKS gene *ASpks1* in its native organism *Acremonium strictum*, where two types of proteins either with whole or R-deficient domains were generated by AS, leading to the biosynthesis of 3-methylorcinaldehyde or 3-MOA upon heterologous expression in *A. oryzae*, respectively (Fig. S5). By contrast, *ASpks1* gene in *A. oryzae* only yielded the transcripts that are translatable into the full PKS protein. Therefore, they proposed that AS of *ASpks1* in its native host served as a regulatory mechanism to modulate the relative production of 3-methylorcinaldehyde and 3-MOA under different circumstances [17]. In our study, expression of the aberrant *preu6β* was regulated by the NMD pathway, in addition, the truncated protein Preu6*β* failed to yield any detectable polyketide products through heterologous expression in *A. oryzae* (Fig. S10). Compounds **9**–**11** could also not be detectable by HRESIMS in the native organism XL-1326 (Fig. S11). Thus, we suppose that *preu6β* represents a nonsense mRNA in XL-1326, which may be exploited as a shunting regulatory strategy to moderate the yield of the *dpe* cluster-derived products in response to environmental conditions, while avoiding potential self-toxicity.

Importantly, we characterized the first natural PANT product **9** from the *S. cerevisiae* transformant of Preu6*β*. Through a combinatorial biosynthesis strategy, we further demonstrated that: (i) inactivation of the TE domain is a prerequisite for the formation of OA derived-PANT products, whereas swapping the ACP domains from different sources of nrPKSs into Preu6*β* do not affect their production; (ii) OA-based monomers and dimers with different methylation patterns were competent to give rise to the corresponding PANT products. In contrast, no PANT products were detected from naphthalene pyrone, anthracenone, or benzenediol lactones, indicating that the capacity of OA derivatives to generate PANT products might be due to their specific structures. As one of the very few experimental examples (Fig. S5) [16,17,28], our study extended the current understanding of the functional scope of AS in fungal PKSs, in parallel, the discovery of OA-originated natural PANT products introduced a previously unrecognized structural class into the natural product repertoire.

## Data availability statement

The data underlying this study are available in the published article and its Supporting Information.

## CRediT authorship contribution statement

**Qingpei Liu:** Data curation, Formal analysis, Funding acquisition, Investigation, Methodology, Project administration, Resources, Supervision, Writing – original draft, Writing – review & editing. **Liwei Zhou:** Data curation, Formal analysis, Investigation, Methodology, Writing – original draft. **Yifu Gong:** Data curation, Formal analysis, Investigation, Methodology. **Xinqiong Liu:** Formal analysis. **Jiajie Ma:** Formal analysis, Writing – original draft. **Wei Liu:** Formal analysis. **Haiyan Fu:** Formal analysis. **Xiaolong Yang:** Conceptualization, Funding acquisition, Project administration, Resources, Supervision, Writing – review & editing.

## Declaration of competing interest

The authors declare that they have no known competing financial interests or personal relationships that could have appeared to influence the work reported in this paper.

## Supporting Information

Plasmids and primers used, RT-PCR analysis, domain analysis, 3D-structure comparison of the SAT domains, antifungal assay results, NMR, HRESIMS, and UV spectra of the related compounds in this study (PDF).
