# Supplementary material for: Alternative splicing-induced thioesterase domain deficiency drives the production of natural orsellinic acid derivatives with a pantetheine moiety in nonreducing polyketide synthases
Source: Synth Syst Biotechnol. 2026 Aug 7;17:115–20. doi: 10.1016/j.synbio.2026.07.008 (PMC13476535; doi:10.1016/j.synbio.2026.07.008)
Supplement: Supplementary file 1 [file mmc1.pdf]

Supporting Information

for

Alternative Splicing-Induced Thioesterase Domain Deficiency Drives the Production of Natural Orsellinic Acid Derivatives with a Pantetheine Moiety in Nonreducing Polyketide Synthases

Qingpei Liu[1,#], Liwei Zhou[1,#], Yifu Gong[1,#], Xinqiong Liu[3], Jiajie Ma[1], Wei Liu[1], Haiyan Fu[1], and Xiaolong Yang[1,2,*]

[1] School of Pharmaceutical Sciences, South-Central Minzu University, Wuhan 430074, P.R. China

[2] Anhui Province Key Laboratory of Bioactive Natural Products, School of Pharmacy, Anhui University of Chinese Medicine, Hefei 230012, P.R. China

[3] College of Life Science, South-Central Minzu University, Wuhan 430074, P.R. China

[#] These authors contributed equally to this work.

* Corresponding author: Xiaolong Yang, Email: 2019001@mail.scuec.edu.cn

Contents

1 SI Materials and Methods 1

1.1 Molecular biology, microbiology, and bioinformatics 1

1.1.1 Construction of the *Saccharomyces cerevisiae* expression plasmids 1

1.1.2 Construction of the *Aspergillus oryzae* expression plasmids 5

1.1.3 Total biosynthesis of the orsellinic acid derivatives 6

1.1.4 Reverse-transcription PCR analysis 7

1.1.5 Amino acid alignments of the OA derivative related nrPKSs 7

1.2 Protein expression in *S. cerevisiae*, and *in vitro* biochemical assay 7

1.3 Isolation and characterization of the OA derivatives 8

1.3.1 General methods 8

1.3.2 Compounds **9-11** 9

1.4 Antifungal bioassays 11

1.4.1 Antifungal activities of compounds **1-8** against seven agricultural pathogenic fungi ... 11

1.4.2 Antifungal activities of compounds **1-8** against the native organism XL-1326 11

2 SI Tables 12

Table S1. Domain analysis of the nrPKSs 12

Table S2. Antifungal activities of compounds **1-8** (MIC, μg/mL)[a] 13

Table S3. Primers used in this study 14

Table S4. Plasmids used in this study 16

Table S5. The HRESIMS data analysis of compounds in this study 18

Table S6. ^{1}H NMR and ^{13}C NMR data 19

Table S6.1. Compound **9** (in methanol-d_4) 19

Table S6.2. Compound **10** (in methanol-d_4) 20

Table S7. The amino acid sequences of Preu6α and Preu6β 21

3 SI Figures 23

Figure S1. Biosynthetic models for the OA derivatives 23

Figure S2. Comparisons between Preu6 and other OA-related nrPKSs 24

Figure S3. Sequence analysis of the *preu6α/β* transcripts 25

Figure S4. Biosynthetic model for DPEs **1-8** 26

Figure S5. Alternative splicing (AS) event in *ASpks1* gene 27

Figure S6. Experimental ECD spectra of compound **9** in MeOH 28

Figure S7. Confirmation of the catalytic activity of Preu6*β* 29

Figure S8. Searching for the corresponding PANT products in the TE-mutated Preu3 and DrcA transformants 30

Figure S9. Functional analysis of the tandem ACP domains of Preu6*β* in the production of compounds **9-11** 31

Figure S10. Metabolite analysis of Preu6*β* expressed in *A. oryzae* 32

Figure S11. HRESIMS analysis of compounds **9-11** in the native strain XL-1326 33

Figure S12. UV-VIS spectra of compounds **9-11** 34

Figure S13. HRESIMS spectra of the related compounds in this study 35

- *Figure S13.1.* (+)-HRESIMS spectrum of **9** 35
- *Figure S13.2.* (+)-HRESIMS spectrum of **10** 35
- *Figure S13.3.* (+)-HRESIMS spectrum of **11** 36
- *Figure S13.4.* (+)-HRESIMS spectrum of **12** 36
- *Figure S13.5.* (-)-HRESIMS spectrum of orsellinic acid 37
- *Figure S13.6.* (-)-HRESIMS spectrum of lecanoric acid 37
- *Figure S13.7.* (+)-HRESIMS spectrum of prephenalenone 38
- *Figure S13.8.* (+)-HRESIMS spectrum of atrochrysone 38
- *Figure S13.9.* (+)-HRESIMS spectrum of desmethyl-lasiodiplodin 39
- *Figure S13.10.* (+)-HRESIMS spectrum of 10,11-dehydrocurvularin 39
- *Figure S13.11.* (-)-HRESIMS spectrum of 3-methylorsellinic acid 40
- *Figure S13.12.* (-)-HRESIMS spectrum of 3-methylorsellinic acid-PANT 40
- *Figure S13.13.* (-)-HRESIMS spectrum of isonorobtusatic acid 41
- *Figure S13.14.* (-)-HRESIMS spectrum of isonorobtusatic acid-PANT 41
- *Figure S13.15.* (-)-HRESIMS spectrum of norobtusatic acid 42
- *Figure S13.16.* (-)-HRESIMS spectrum of norobtusatic acid-PANT 42
- *Figure S13.17.* (-)-HRESIMS spectrum of 4-*O*-demethylbarbatic acid 43
- *Figure S13.18.* (-)-HRESIMS spectrum of 4-*O*-demethylbarbatic acid-PANT 43

Figure S13.19. (-)-HRESIMS spectrum of CJ-20,557 44

Figure S13.20. (-)-HRESIMS spectrum of CJ-20,557-PANT 44

Figure S14. NMR spectra of the OA derivatives 45

Figure S14.1. ^{1}H NMR spectrum of **9** in methanol-d_4 45

Figure S14.2. ^{13}C NMR spectrum of **9** in methanol-d_4 46

Figure S14.3. DEPT 135 spectrum of **9** in methanol-d_4 47

Figure S14.4. HSQC spectrum of **9** in methanol-d_4 48

Figure S14.5. HMBC spectrum of **9** in methanol-d_4 49

Figure S14.6. ^{1}H-^{1}H COSY spectrum of **9** in methanol-d_4 50

Figure S14.7. ^{1}H NMR spectrum of **10** in methanol-d_4 51

Figure S14.8. ^{13}C NMR spectrum of **10** in methanol-d_4 52

Figure S14.9. HSQC spectrum of **10** in methanol-d_4 53

Figure S14.10. HMBC spectrum of **10** in methanol-d_4 54

Figure S14.11. ^{1}H-^{1}H COSY spectrum of **10** in methanol-d_4 55

Figure S14.12. ^{1}H NMR spectrum of **11** in methanol-d_4 56

Figure S14.13. ^{13}C NMR spectrum of **11** in methanol-d_4 57

REFERENCE 58

1 SI Materials and Methods

1.1 Molecular biology, microbiology, and bioinformatics

1.1.1 Construction of the *Saccharomyces cerevisiae* expression plasmids

The primers used in this study are listed in **Table S3**. The plasmids are summarized in **Table S4**.

The YEpADH2p-FLAG-URA plasmids harboring the encoding genes for the nonreducing polyketide synthases (nrPKSs), Preu3 (YEpPreu3),[1-2] Preu6 (also known as DpeA, renamed as Preu6*α* in this study, YEpPreu6*α*),[2] Preu6(H2177A) [renamed as Preu6*α*(H2177A), YEpPreu6*α*(H2177A)],[3] Preu4 (YEpPreu4),[4] Preu7 (YEpPreu7),[5] Preu8 (YEpPreu8),[6] LtLasS2 (YEpLtLasS2),[7] and AtCurS2 (YEpAtCurS2);[8] and YEpADH2p-FLAG-TRP plasmids carrying the encoding genes for the highly reducing PKSs (hrPKSs), LtLasS1 (YEpLtLasS1),[7] and AtCurS1 (YEpAtCurS1),[8] respectively, were conducted as previously described. The Udwary-Merski algorithm was used to predict domain boundaries in some of the nrPKSs **(Tables S1)**.[9]

The YEpADH2p-FLAG-URA plasmid carrying the *preu6β* gene was constructed as follows. Using YEpPreu6*α* as the template, a 991-bp fragment (Preu6*β*-1) and a 941-bp fragment (Preu6*β*-2) were amplified with primer pairs Preu6*α*-BglII-F/Preu6*β*-R and Preu6*β*-F/Preu6*α*-3R, respectively. Then, Preu6*β*-1 and Preu6*β*-2, which contain appropriate overlapping segments, were merged with the *Bgl*II/*Pme*I fragment of YEpPreu6*α* (10,970 bp) using the SE Seamless Cloning and Assembly Kit (ZOMANBIO, Beijing, China) to yield plasmid YEpPreu6*β*.

The YEpADH2p-FLAG-URA plasmid carrying the encoding gene for DrcA-Δ(MT+TE) was constructed by deleting the gene segment encoding the *C*-methyltransferase (MT) and thioesterase (TE) domains of DrcA (A1,928-T2,701). Using the synthesized cDNA fragments of *drcA* as templates,[10] a 1,200-bp fragment (DrcA-1), a 2,381-bp fragment (DrcA-2), and a 2,320-bp fragment [DrcA-Δ(MT+TE)-3] were amplified with primer pairs DrcA-1F/DrcA-1R, DrcA-2F/DrcA-2R, and DrcA-3F/DrcA-ACP-R, respectively. Then, DrcA-1, DrcA-2, and DrcA-Δ(MT+TE)-3, which contain appropriate overlapping segments, were merged with the *Nde*I/*Pme*I fragment of YEpADH2p-FLAG-URA (6,257 bp) using the SE Seamless Cloning and Assembly Kit to yield plasmid YEpDrcA-Δ(MT+TE).

The YEpADH2p-FLAG-URA plasmid carrying the encoding gene for AN7909-ΔTE was constructed by deleting the gene segment encoding the TE domain of AN7909 (D1,843-E2,103). Using the synthesized cDNA fragments of *AN7909* as templates,[11] a 984-bp fragment (AN7909-1), a 2,277-bp

fragment (AN7909-2), and a 2,385-bp fragment (AN7909-ΔTE-3) were amplified with primer pairs AN7909-1F/AN7909-1R, AN7909-2F/AN7909-2R, and AN7909-3F/AN7909-ACPlinker-R, respectively. Then, AN7909-1, AN7909-2, and AN7909-ΔTE-3, which contain appropriate overlapping segments, were merged with the *Nde*I/*Pme*I fragment of YEpADH2p-FLAG-URA (6,257 bp) using the SE Seamless Cloning and Assembly Kit to yield plasmid YEpAN7909-ΔTE.

The YEpADH2p-FLAG-URA plasmid carrying the encoding gene for Preu3-Δ(MT+TE) was constructed by deleting the gene segment encoding the MT and TE domains of Preu3 (V1,789-A2,523). Using YEpPreu3 as the template, a 2,522-bp fragment (Preu3-1), a 2,554-bp fragment (Preu3-2), and a 405-bp fragment [Preu3-Δ(MT+TE)-3] were amplified with primer pairs Preu3-1F/Preu3-1R, Preu3-2F/Preu3-2R, and Preu3-3F/Preu3-ACPlinker-R, respectively. Then, Preu3-1, Preu3-2, and Preu3-Δ(MT+TE)-3, which contain appropriate overlapping segments, were merged with the *Nde*I/*Pme*I fragment of YEpADH2p-FLAG-URA (6,257 bp) using the SE Seamless Cloning and Assembly Kit to yield plasmid YEpPreu3-Δ(MT+TE).

The YEpADH2p-FLAG-URA plasmid carrying chimeric Preu6β(ACP_{AN7909}) was constructed by replacing the gene segment (D1,648-S1,891, ACP1-ACP2-linker) of *preu6β* with the gene segment (R1,589-L1,842, linker-ACP1-ACP2-linker) of *AN7909*. A 235-bp fragment, [Preu6β(ACP_{AN7909})-1], was amplified with primer pair Preu6α-BglII-F/AN7909-BglII-R using YEpPreu6β as the template. Next, an 822-bp fragment, [Preu6β(ACP_{AN7909})-2], was amplified with primer pair AN7909-ACPlinker-F/AN7909-ACPlinker-R using the synthesized cDNA fragment of *AN7909* as the template. Then, Preu6β(ACP_{AN7909})-1 and Preu6β(ACP_{AN7909})-2, which contain appropriate overlapping segments, were merged with the *Bgl*II/*Pme*I fragment of YEpPreu6β (10,970 bp) using the SE Seamless Cloning and Assembly Kit to yield plasmid YEpPreu6β(ACP_{AN7909}).

The YEpADH2p-FLAG-URA plasmid carrying chimeric Preu6β(ACP_{DrcA}) was constructed by replacing the gene segment (D1,648-S1,891, ACP1-ACP2-linker) of *preu6β* with the gene segment (A1,659-A1,927, ACP1-ACP2-ACP3) of *DrcA*. An 867-bp fragment, [Preu6β(ACP_{DrcA})-2], was amplified with primer pair DrcA-ACP-F/DrcA-ACP-R using the synthesized cDNA fragment of *drcA* as the template. Then, Preu6β(ACP_{AN7909})-1 and Preu6β(ACP_{DrcA})-2, which contain appropriate overlapping segments, were merged with the *Bgl*II/*Pme*I fragment of YEpPreu6β (10,970 bp) using the SE Seamless Cloning and Assembly Kit to yield plasmid YEpPreu6β(ACP_{DrcA}).

The YEpADH2p-FLAG-URA plasmid carrying chimeric Preu6β(ACP$_{Preu3}$) was constructed by replacing the gene segment (D1,648-S1,891, ACP1-ACP2-linker) of *preu6β* with the gene segment (Q1,586-P1,788, linker-ACP-linker) of *preu3*. A 672-bp fragment, [Preu6β(ACP$_{Preu3}$)-2], was amplified with primer pair Preu3-ACPlinker-F/Preu3-ACPlinker-R using YEpPreu3 as the template. Then, Preu6β(ACP$_{AN7909}$)-1 and Preu6β(ACP$_{Preu3}$)-2, which contain appropriate overlapping segments, were merged with the *Bgl*II/*Pme*I fragment of YEpPreu6β (10,970 bp) using the SE Seamless Cloning and Assembly Kit to yield plasmid YEpPreu6β(ACP$_{Preu3}$).

The YEpADH2p-FLAG-URA plasmid carrying chimeric Preu6β(ACP$_{Preu4}$) was constructed by replacing the gene segment (D1,648-S1,891, ACP1-ACP2-linker) of *preu6β* with the gene segment (S1,672-L1,845, ACP1-ACP2) of *preu4*. A 582-bp fragment, [Preu6β(ACP$_{Preu4}$)-2], was amplified with primer pair Preu4-ACP-F/Preu4-ACP-R using YEpPreu4 as the template. Then, Preu6β(ACP$_{AN7909}$)-1 and Preu6β(ACP$_{Preu4}$)-2, which contain appropriate overlapping segments, were merged with the *Bgl*II/*Pme*I fragment of YEpPreu6β (10,970 bp) using the SE Seamless Cloning and Assembly Kit to yield plasmid YEpPreu6β(ACP$_{Preu4}$).

The YEpADH2p-FLAG-URA plasmid carrying chimeric Preu6β(ACP$_{Preu7}$) was constructed by replacing the gene segment (D1,648-S1,891, ACP1-ACP2-linker) of *preu6β* with the gene segment (P1,677-S1,821, linker-ACP-linker) of *preu7*. A 498-bp fragment, [Preu6β(ACP$_{Preu7}$)-2], was amplified with primer pair Preu7-ACPlinker-F/Preu7-ACPlinker-R using YEpPreu7 as the template. Then, Preu6β(ACP$_{AN7909}$)-1 and Preu6β(ACP$_{Preu7}$)-2, which contain appropriate overlapping segments, were merged with the *Bgl*II/*Pme*I fragment of YEpPreu6β (10,970 bp) using the SE Seamless Cloning and Assembly Kit to yield plasmid YEpPreu6β(ACP$_{Preu7}$).

The YEpADH2p-FLAG-URA plasmid carrying chimeric Preu6β(ACP$_{Preu8}$) was constructed by replacing the gene segment (D1,648-S1,891, ACP1-ACP2-linker) of *preu6β* with the gene segment (S1,655-L1,849, ACP1-ACP2) of *preu8*. A 645-bp fragment, [Preu6β(ACP$_{Preu8}$)-2], was amplified with primer pair Preu8-ACP-F/Preu8-ACP-R using YEpPreu8 as the template. Then, Preu6β(ACP$_{AN7909}$)-1 and Preu6β(ACP$_{Preu8}$)-2, which contain appropriate overlapping segments, were merged with the *Bgl*II/*Pme*I fragment of YEpPreu6β (10,970 bp) using the SE Seamless Cloning and Assembly Kit to yield plasmid YEpPreu6β(ACP$_{Preu8}$).

YEpADH2p-FLAG-URA plasmid carrying mutated DrcA(H2663A) was constructed by replacing the histidine at position 2,663 of DrcA with an alanine. Using the synthesized cDNA fragments of *drcA*

as templates, a 2,642-bp fragment DrcA(H2663A)-1 and a 177-bp fragment DrcA(H2663A)-2 were amplified with primer pairs DrcA-BglII-F/DrcA-H2663A-R and DrcA-H2663A-F/DrcA-TE-R. Then, DrcA(H2663A)-1 and DrcA(H2663A)-2, which contain appropriate overlapping segments, were merged with the *Bgl*II/*Pme*I fragment of YEpDrcA-Δ(MT+TE) (11,662 bp) using the SE Seamless Cloning and Assembly Kit to yield plasmid YEpDrcA(H2663A).

YEpADH2p-FLAG-URA plasmid carrying mutated Preu3(H2496A) was constructed by replacing the histidine at position 2,496 of Preu3 with an alanine. Using YEpPreu3 as the template, an 819-bp fragment Preu3(H2496A)-1 and a 144-bp fragment Preu3(H2496A)-2 were amplified with primer pairs Preu3-BglII-F/Preu3-H2496A-R and Preu3-H2496A-F/Preu3-TE-R. Then, Preu3(H2496A)-1 and Preu3(H2496A)-2, which contain appropriate overlapping segments, were merged with the *Bgl*II/*Pme*I fragment of YEpPreu3-Δ(MT+TE) (12,984 bp) using the SE Seamless Cloning and Assembly Kit to yield plasmid YEpPreu3(H2496A).

YEpADH2p-FLAG-URA plasmid carrying mutated Preu4(H2128A) was constructed by replacing the histidine at position 2,128 of Preu4 with an alanine. Using YEpPreu4 as the template, a 226-bp fragment Preu4(H2128A)-1 and a 132-bp fragment Preu4(H2128A)-2 were amplified with primer pairs Preu4-RruI-F/Preu4-H2128A-R and Preu4-H2128A-F/Preu4-TE-R. Then, Preu4(H2128A)-1 and Preu4(H2128A)-2, which contain appropriate overlapping segments, were merged with the *Rru*I/*Pme*I fragment of YEpPreu4 (12,473 bp) using the SE Seamless Cloning and Assembly Kit to yield plasmid YEpPreu4(H2128A).

YEpADH2p-FLAG-URA plasmid carrying mutated LtLasS2(H2058A) was constructed by replacing the histidine at position 2,058 of LtLasS2 with an alanine. Using YEpLtLasS2 as the template, a 1,825-bp fragment LtLasS2(H2058A)-1 and a 147-bp fragment LtLasS2(H2058A)-2 were amplified with primer pairs LtLasS2-RruI-F/LtLasS2-H2058A-R and LtLasS2-H2058A-F/LtLasS2-TE-R. Then, LtLasS2(H2058A)-1 and LtLasS2(H2058A)-2, which contain appropriate overlapping segments, were merged with the *Rru*I/*Pme*I fragment of YEpLtLasS2 (10,646 bp) using the SE Seamless Cloning and Assembly Kit to yield plasmid YEpLtLasS2(H2058A).

YEpADH2p-FLAG-URA plasmid carrying mutated AtCurS2(H2055A) was constructed by replacing the histidine at position 2,055 of AtCurS2 with an alanine. Using YEpAtCurS2 as the template, a 1,479-bp fragment AtCurS2(H2055A)-1 and a 147-bp fragment AtCurS2(H2055A)-2 were amplified with primer pairs AtCurS2-Eco91I-F/AtCurS2-H2055A-R and AtCurS2-H2055A-F/AtCurS2-TE-R.

Then, AtCurS2(H2055A)-1 and AtCurS2(H2055A)-2, which contain appropriate overlapping segments, were merged with the *Eco*91I/*Pme*I fragment of YEpAtCurS2 (11,001 bp) using the SE Seamless Cloning and Assembly Kit to yield plasmid YEpAtCurS2(H2055A).

YEpADH2p-FLAG-URA plasmid carrying mutated YEpPreu6*β*(S1681A) was constructed by replacing the serine at position 1,681 of Preu6*β* with an alanine. Using YEpPreu6*β* as the template, a 349-bp fragment Preu6*β*(S1681A)-1 and a 788-bp fragment Preu6*β*(S1681A)-2 were amplified with primer pairs Preu6*β*-S1681A-1F/Preu6*β*-S1681A-1R and Preu6*β*-S1681A-2F/Preu6*β*-S1681A-2R. Then, Preu6*β*(S1681A)-1 and Preu6*β*(S1681A)-2, which contain appropriate overlapping segments, were merged with the *Kpn2*I/*Bgl*II fragment of YEpPreu6*β* (11,760 bp) using the SE Seamless Cloning and Assembly Kit to yield plasmid YEpPreu6*β*(S1681A).

YEpADH2p-FLAG-URA plasmid carrying mutated YEpPreu6*β*(S1790A) was constructed by replacing the serine at position 1,790 of Preu6*β* with an alanine. Using YEpPreu6*β* as the template, a 676-bp fragment Preu6*β*(S1790A)-1 and a 1,223-bp fragment Preu6*β*(S1790A)-2 were amplified with primer pairs Preu6*β*-S1681A-1F/Preu6*β*-S1790A-1R and Preu6*β*-S1790A-2F/Preu6*α*-3R. Then, Preu6*β*(S1790A)-1 and Preu6*β*(S1790A)-2, which contain appropriate overlapping segments, were merged with the *Pme*I/*Bgl*II fragment of YEpPreu6*β* (10,970 bp) using the SE Seamless Cloning and Assembly Kit to yield plasmid YEpPreu6*β*(S1790A).

YEpADH2p-FLAG-URA plasmid carrying mutated YEpPreu6*β*(S1681,1790A) was constructed by replacing the serine at position 1,681 and 1,790 of Preu6*β* with an alanine. Using YEpPreu6*β*(S1681A) as the template, a 676-bp fragment Preu6*β*(S1681,1790A)-1 and a 1,223-bp fragment Preu6*β*(S1790A)-2 were amplified with primer pairs Preu6*β*-S1681A-1F/Preu6*β*-S1790A-1R and Preu6*β*-S1790A-2F/Preu6*α*-3R. Then, Preu6*β*(S1681,1790A)-1 and Preu6*β*(S1790A)-2, which contain appropriate overlapping segments, were merged with the *Pme*I/*Bgl*II fragment of YEpPreu6*β* (10,970 bp) using the SE Seamless Cloning and Assembly Kit to yield plasmid YEpPreu6*β*(S1681,1790A).

All newly constructed plasmids were confirmed by DNA sequencing.

1.1.2 Construction of the *Aspergillus oryzae* expression plasmids

The primers used in this study are listed in **Table S3**. The plasmids are summarized in **Table S4**. The plasmid harboring the encoding gene for DpeA [renamed as Preu6*α* in this study, pUSA(Preu6*α*)],[12] was conducted as previously described.

The pUSA plasmid harboring the encoding gene for Preu6*β*, pUSA(Preu6*β*), was constructed as follows. Using YEpPreu6*β* as the template, a 2,214-bp fragment (pUSA-Preu6*β*-1), a 2,230-bp fragment (pUSA-Preu6*β*-2), and a 2,224-bp fragment (pUSA-Preu6*β*-3) were amplified with primer pairs pUSA-Preu6*β*-1F/pUSA-Preu6*β*-1R, pUSA-Preu6*β*-2F/pUSA-Preu6*β*-2R, and pUSA-Preu6*β*-3F/pUSA-Preu6*β*-3R, respectively. Then, pUSA-Preu6*β*-1, pUSA-Preu6*β*-2, and pUSA-Preu6*β*-3, which contain appropriate overlapping segments, were merged with the *Kpn*I fragment of pUSA (7,133 bp) using the SE Seamless Cloning and Assembly Kit to yield plasmid pUSA(Preu6*β*), which was confirmed by DNA sequencing.

1.1.3 Total biosynthesis of the orsellinic acid derivatives

1.1.3.1 Total biosynthesis in *S. cerevisiae*

For the production of all orsellinic acid (OA) derivatives, the respective expression plasmids were independently introduced into *S. cerevisiae* BJ5464-NpgA cells (*MATα*, *ura3-52*, *his3-Δ200*, *leu2-Δ1*, *trp1*, *pep4*::*HIS3*, *prb1-Δ1.6R*, *can1*, *GAL*) using the LiAc-PEG4000 method,[13,14] and the transformants were selected on appropriate synthetic drop-out (SD) plates.[3] Three to five independent *S. cerevisiae* transformants were performed for the production of OA derivatives, with fermentations of representative isolates repeated at least three times. Recombinant yeast cells were cultured in 150 mL Erlenmeyer flasks containing 25 mL of the appropriate SD medium at 30ºC with shaking at 200 rpm until the OD_{600} reached 0.6. Then, an equal volume of YPD medium (1% yeast extract, 2% peptone, and 1% dextrose) was added, and the fermentation continued under the same conditions for an additional 72 h.[2]

1.1.3.2 Total biosynthesis in *A. oryzae*

The appropriate expression plasmids were transformed into *A. oryzae* NSAR1 (*niaD-* , *sC-* , Δ*argB*, *adeA-*) by the protoplast-polyethylene glycol method, respectively, and the transformants were selected on the appropriate nutrient deficient media plates.[12] For each transformation, at least twelve independent *A. oryzae* transformants were performed for the production of OA derivatives, and fermentations with representative isolates repeated at least three times. The selected transformants were cultured in 500 mL Erlenmeyer flasks containing 150 mL of DPY medium (2% dextrin, 1% polypeptone, 2.5% yeast extract, 0.5% KH_2PO_4, and 0.05% $MgSO_4{\cdot}7H_2O$) at 30ºC with shaking at 160 rpm for 4 days. The equivalent Czapek-Dox (CD) medium with hipolypepton (10 g/L) and starch (20 g/L) was then added to induce expression under the *α*-amylase promoter, then the cultures were shaken for another 4 days.

1.1.4 Reverse-transcription PCR analysis

Total RNA was extracted from the mycelia of *Preussia isomera* XL-1326 cultivated on the cellophane-covered potato dextrose agar (PDA) plates for 4, 6, 8, 10, 12, and 14 days at 28ºC. First-strand cDNA was synthesized using the PrimeScript II First Strand cDNA Synthesis Kit (TAKARA, Shiga, Japan) following the manufacturer's instructions. To analyze the expression level of *preu6α/β*, three independent biological replicates were performed, each with three technical replicates. The RT-PCR reaction mixture (20.0 μL total volume) consisted of 10.0 μL of DreamTaq™ Green PCR Master Mix (2×) (Thermo Fisher Scientific, Vilnius, Lithuania), 1.0 μL each of 10 μM forward and reverse primer, 75 ng of cDNA template, and nuclease-free water up to the total volume. The PCR was carried out in a T100 Thermal Cycler (Bio-Rad, Hercules, CA, USA) under the following conditions: initial denaturation at 98ºC for 3 min, 29 amplification cycles at 98ºC for 5 s, 60ºC for 10 s, and 72ºC for 30 s with a final extension step at 72ºC for 5 min. The *β-actin* gene served as reference. A 232-bp fragment Preu6*α1* and a 162-bp fragment Preu6*β* were amplified with primer pair Preu6-F/Preu6-R1. The primers used are listed in **Table S3**.

To investigate the effect of the nonsense-mediated mRNA decay (NMD) pathway on the degradation of Preu6*α/β*, a prepared cycloheximide stock solution was added into the PDA medium to a final concentration of 5 μg/mL. The mycelia of XL-1326, treated with or without cycloheximide, were collected at 6, 10, and 14 days of incubation. We extracted total RNA from these mycelia and performed RT-PCR. Besides fragments Preu6*α1* and Preu6*β*, a 172-bp fragment Preu6*α2* was amplified with primer pair Preu6-F/Preu6-R2. The subsequent experimental procedures were performed as described above.

1.1.5 Amino acid alignments of the OA derivative related nrPKSs

Based on the domain division in **Table S1**, percent identities between the indicated domains were conducted using BLASTP (https://blast.ncbi.nlm.nih.gov/Blast.cgi). In addition, we predicted the 3D structures of the SAT domains in Preu6, AN7909, Preu3, and DrcA using AlphaFold3 (https://alphafoldserver.com/), respectively.[15] We downloaded the predicted 3D models, then submitted to US-align (https://www.aideepmed.com/US-align/) to obtain the TM-score.[16] Structural visualization and comparative analyses were subsequently performed using ChimeraX.[17]

1.2 Protein expression in *S. cerevisiae*, and *in vitro* biochemical assay

YEpADH2p-FLAG-URA plasmid carrying Preu6*β* plus 9×His tag was constructed as follows. Using YEpPreu6*β* as the template, a 1,030-bp fragment Preu6*β*-C9his-1 and a 966-bp fragment Preu6*β*-C9his-

2 were amplified with primer pairs Preu6*β*-C9his-1F/Preu6*β*-C9his-1R and Preu6*β*-C9his-2F/Preu6*β*-C9his-2R. Then, Preu6*β*-C9his-1 and Preu6*β*-C9his-2, which contain appropriate overlapping segments, were merged with the *Pme*I/*Bgl*II fragment of YEpPreu6*β* (10,970 bp) using the SE Seamless Cloning and Assembly Kit to yield plasmid YEpPreu6*β*(C9His), which was confirmed by DNA sequencing.

3 μL of plasmid YEpPreu6*β*(C9His) was transformed into *S. cerevisiae* strain BJ5464-NpgA. The cultivation of yeast was performed as described in **Section 1.1.3.1**. The cells were harvested by centrifugation (2500 g, 20 minutes, 4ºC), resuspended in 20 mL lysis buffer (50 mM NaH_2PO_4, pH 8.0, 0.15 M NaCl, 10 mM imidazole), and lysed through sonication on ice. Cellular debris was removed by centrifugation (35,000 g, 1 hour, 4ºC).[18]

The *in vitro* enzymatic reactions were performed in 50 mM Tris-HCl buffer (pH 7.5) containing 2 mM acetyl-CoA, 3 mM malonyl-CoA, 4 mM NADPH, and crude enzyme extracts at a final protein concentration of 5 mg/mL, in a total volume of 100 μL. After incubation at 25ºC for 20 h, the reactions were quenched by adding 200 μL of ethyl acetate (EtOAc). Then, the organic phase was collected and evaporated to dryness, redissolved in MeOH and analyzed by LC-MS.[19]

1.3 Isolation and characterization of the OA derivatives

1.3.1 General methods

Upon completion of the fermentations, *S. cerevisiae* BJ5464-NpgA cultures were adjusted to pH 5.0 and extracted with equal volumes of ethyl acetate; *A. oryzae* NSAR1 cultures (300 mL/flask) were extracted with 200 mL ethyl acetate with shaking at 200 rpm for 0.5 h. Both *S. cerevisiae* BJ5464-NpgA and *A. oryzae* NSAR1 cultures were extracted three times each. The crude extracts were pooled, dried *in vacuo*, and redissolved in methanol. Samples were routinely analyzed *via* ultra-high-performance liquid chromatography (UHPLC) on a Thermo Scientific UltiMate 3000 system, fitted with a Sunniest PFP & C18 column (250 × 4.6 mm, 5.0 μm, ChromaNik Technologies). The mobile phases consisted of water (A, containing 0.1% acetic acid) and methanol (B), and the flow rate was kept at 1.0 mL/min. The system was run with the following gradient program: from 5% B to 100% B for 40 min, then kept at 100% B for 5 min. The column was balanced with 5% B for 4 min before each sample injection. Absorbance was monitored with the diode array detector (DAD) of UltiMate 3000 at 210, 254, 270 and 300 nm wavelength.

For isolation of the OA derivatives, the crude extracts were initially fractionated by octadecylsilyl (ODS) column chromatography eluted with a stepwise gradient of methanol/water. The resulting

fractions containing the target compounds were subsequently applied to silica gel column chromatography eluted with gradient dichloromethane/methanol, then purified by semi-preparative HPLC on an Agilent 1260 Infinity II system. UHPLC-HRESIMS spectra were acquired on a Thermo Scientific UltiMate 3000 UHPLC coupled with a Thermo Scientific Q Exactive mass spectrometer operated in positive and negative ion mode using full scanning of 150-1000 *m/z*; capillary voltage of 3.8 kV; capillary temperature of 325ºC; sheath gas flow velocity of 40 arb; auxiliary gas velocity of 20 arb; auxiliary gas heating temperature of 350ºC.

To identify the target peaks, we extracted ion chromatograms for the expected ions in the corresponding positive- and negative-ion modes, and compared the observed masses with the theoretical values to calculate the mass errors according to the formula (**Table S5**):

$$Mass\ error\ (ppm) = \frac{m_{observed} - m_{calculated}}{m_{calculated}} \times 10^6$$

^1H, ^{13}C, and 2D NMR, including proton-proton correlation spectroscopy (^1H-^1H COSY), heteronuclear single quantum coherence (HSQC), heteronuclear multiple bond correlation (HMBC) spectra were recorded on a Bruker Avance III^{TM} HD 600 MHz spectrometer in CD_3OD as the solvent. Chemical shift values (δ) are given in parts per million (ppm), and the coupling constants (*J* values) are in Hz. Chemical shifts were referenced to the residual solvent peaks of methanol-d_4. Optical rotations were recorded on a Rudolph Autopol IV automatic polarimeter (Rudolph Research Analytical, Hackettstown, U.S.A.). Measured values of circular dichroism (CD) were acquired with a JASCO-810 instrument (JASCOCo., Ltd., Tokyo, Japan). Unless otherwise stated, chemicals and solvents were of reagent grade and used as obtained from commercial sources.

Conformational searches were performed using molecular mechanics with the MMFF94s force field, and only conformers with populations higher than 1% were considered for further study. These conformers were fully optimized by density functional theory (DFT) at the B3LYP/6-31G(d) level using the Gaussian 09 program package (Gaussian, Inc., Wallingford, CT, USA). The optimized predominant conformers were then used for ECD calculations by time-dependent density functional theory (TDDFT) at the B3LYP/6-31G (d,p) level in MeOH, employing the IEFPCM solvation model.[20]

1.3.2 Compounds 9-11

The crude extract (2.55 g) of an 8 L fermentation broth of *S. cerevisiae* BJ5464-NpgA carrying plasmid YEpPreu6β was subjected to ODS column, eluted with a gradient of methanol/water (20:80,

40:60, 60:40, 80:20, v/v) to yield fractions A-D. After UHPLC-MS analysis, fraction C (170.3 mg) was further applied to silica gel column and eluted with gradient dichloromethane/methanol (98:2, 94:6, v/v) to yield fractions E-F. Fraction E was then purified by semi-preparative HPLC on an Agilent 1260 Infinity II system with an Agilent 1260 diode array detector, fitted with a Sunniest PFP & C18 column (250 × 4.6 mm, 5.0 µm, ChromaNik Technologies), and eluted with a gradient of MeOH/H_2O (0-15 min, 70:30, v/v) to yield compounds **10** (20.3 mg, t_R=8.1 min) and **11** (8.2 mg, t_R=9.9 min). Fraction F just contained pure compound **9** (8.8 mg).

Compound **9** was obtained as a colorless oil with the molecular formula $C_{27}H_{34}N_2O_{10}S$ determined by HRESIMS (*m/z* 579.20056 $[M+H]^+$, calcd. for $C_{27}H_{35}N_2O_{10}S$, 579.2012). The UV spectrum of **9** showed absorption maxima at 311.0 nm (**Figure S12**). The ^1H and ^{13}C NMR spectra revealed 27 proton and 27 carbon signals (**Table S6.1**). Examination of the HRESIMS, NMR, and UV data suggests that this polyketide derived from a lecanoric acid (LA) core, with twelve units of unsaturation that can be attributed to two aromatic rings and four double bonds. The ^1H NMR spectrum displayed four methyl signals at δ 0.92 (6H, s), 2.56 (s) and 2.29 (s), as well as four methylene signals at δ 2.45 (t, J = 6.6 Hz), 3.21 (t, J = 6.6 Hz), 3.47 (overlap), and 3.49 (overlap). Examination of the ^{13}C NMR and DEPT 135 spectra showed a total of 27 carbon resonances, including 13 quaternary carbons at δ 196.6, 176.1, 174.0, 171.0, 166.5, 164.5, 156.3, 152.9, 144.7, 138.3, 127.1, 105.3 and 40.4; five methine carbons at δ 115.6, 112.9, 108.2, 101.9 and 77.3; five methylene carbons at δ 70.3, 40.0, 36.4, 36.3 and 29.9; and four methyl carbons at δ 24.3, 21.3, 20.9, and 19.2. ^1H-^1H COSY correlations were observed from H-9/H-10 and H-12/H-13. These connectivities, together with key HMBC correlations from H-9 to C-1 and C-10; H-12 to C-11 and C-13; H-13 to C-11 and C-14; H-15 to C-14, C-16, and C-17; H-18 to C-15 and C-17; H-19 to C-15 and C-17; and the HRESIMS data as well, indicated the presence of a pantetheine (PANT) moiety (**Figures S13.1, 14.1-14.6**). Accordingly, the structure of compound **9** was elucidated as a conjugate composed of a LA-derived polyketide moiety and a PANT moiety. In addition, the measured specific rotation of compound **9**, $[\alpha]_D^{21}$ +21 (*c* 0.05, CH_3OH), was consistent with that of the **2-PANT**, $[\alpha]_D^{21}$ +28 (*c* 0.05, CH_3OH), which was obtained *via* chemical synthesis in our earlier work.[3]

Compound **10** was obtained as a colorless oil with the molecular formula $C_{16}H_{16}O_5$ determined by HRESIMS (*m/z* 289.10690 $[M+H]^+$, calcd. for $C_{16}H_{17}O_5$, 289.1076). The UV spectrum of **10** showed absorption maxima at 302.0 nm (**Figure S12**). The ^1H and ^{13}C NMR spectra revealed 13 proton and 16 carbon signals (**Table S6.2**). The ^1H NMR spectrum displayed one methyl signal at δ 2.58 (s), two

methylene signals at *δ* 2.85 (t, *J* = 6.9 Hz) and 3.77 (t, *J* = 6.9 Hz), and four methine signals at *δ* 6.22 (d, *J* = 2.5, 1H), 6.29 (d, *J* = 2.5, 1H), 7.12 (d, *J* = 8.3, 1H) and 7.31 (d, *J* = 8.3, 1H). The ^{13}C NMR spectrum displayed seven quaternary carbon signals at *δ* 171.6, 166.6, 164.5, 150.0, 144.8, 138.5 and 105.4; six methine carbons at *δ* 131.1, 131.1, 122.8, 122.8, 112.9 and 101.9; two methylene carbons at *δ* 64.1 and 39.6; and one methyl carbon at *δ* 24.4. ^1H-^1H COSY correlations were observed from the H-9/H-10, H-12/H-13, and H-15/H-16. These connectivities, together with key HMBC correlations from H-9 to C-10 and C-11; H-10 to C-11, C-12, and C-16, demonstrated the assembly of the carbon framework (**Figures S13.2, 14.7-14.11**). Taken together with the biosynthetic pathway, these spectroscopic data established compound **10** to be *p*-hydroxyphenylethyl orsellinate.

Compound **11** was obtained as a white solid with the molecular formula $C_{10}H_{12}O_4$ determined by HRESIMS (*m/z* 197.08089 $[M+H]^+$, calcd. for $C_{10}H_{13}O_4$, 197.0814) (**Figure S13.3**). The UV spectrum of **11** showed absorption maxima at 307.0 nm (**Figure S12**). The ^1H and ^{13}C NMR spectra were consistent with those reported for ethyl orsellinate in the literature (**Figures S14.12-14.13**).[21]

1.4 Antifungal bioassays

1.4.1 Antifungal activities of compounds 1-8 against seven agricultural pathogenic fungi

Compounds **1-8** were evaluated for their antifungal activities against seven agricultural pathogenic fungi: *Verticillium dahliae* Kleb, *Rhizoctonia solani, Sclerotinia sclerotiorum, Fusarium oxysporum* f.sp. Vasinfectum, *Alternaria fragriae, Helminthosporium maydis*, and *Botryosphaeria berengeriana*. Modified antifungal bioassays were conducted as described previously.[22] Media containing 1% DMSO and ketoconazole were used as the negative and positive controls, respectively.

1.4.2 Antifungal activities of compounds 1-8 against the native organism XL-1326

The inhibitory effects of compounds **1-8** from the *dpe* gene cluster on the growth of *P. isomera* XL-1326 were assessed using a modified confrontation culture method.[23]

2 SI Tables

Table S1. Domain analysis of the nrPKSs

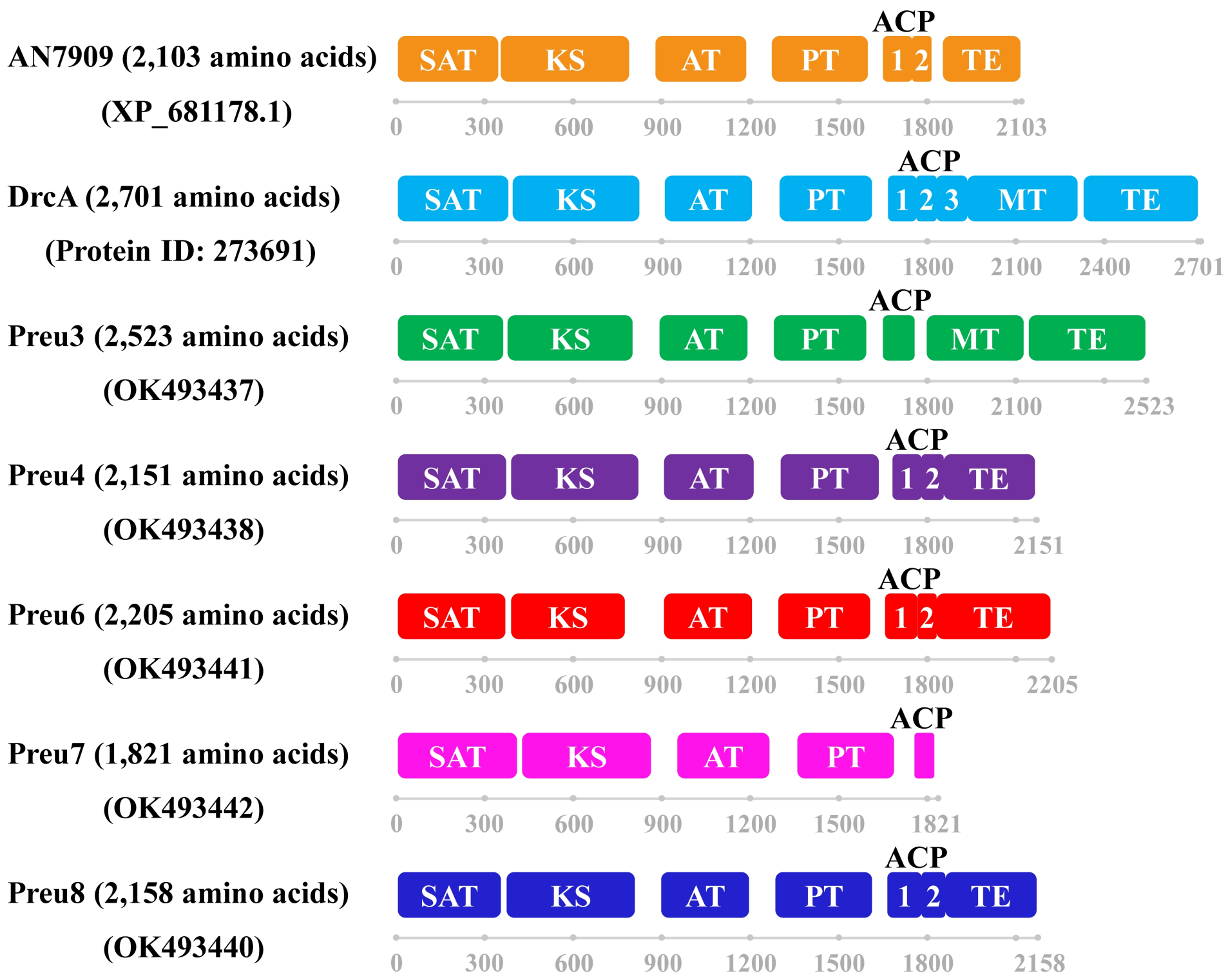

nrPKSs	SAT	KS	AT	PT	ACP	TE	MT
**AN7909**	M^{1}-P^{336}	D^{349}-P^{783}	K^{871}-Q^{1177}	R^{1265}-T^{1588}	ACP1:D^{1641}-G^{1740}<br>ACP2:P^{1741}-G^{1805}	D^{1843}-E^{2103}	——
**DrcA**	M^{1}-A^{375}	S^{390}-P^{816}	V^{904}-S^{1199}	L^{1293}-V^{1605}	ACP1:A^{1659}-S^{1754}<br>ACP2:N^{1755}-T^{1822}<br>ACP3:A^{1823}-A^{1927}	A^{2319}-T^{2701}	A^{1928}-G^{2301}
**Preu3**	M^{1}-A^{356}	D^{373}-S^{794}	L^{886}-P^{1182}	L^{1273}-A^{1585}	D^{1640}-S^{1747}	A^{2132}-A^{2523}	V^{1789}-G^{2114}
**Preu4**	M^{1}-R^{368}	D^{383}-A^{813}	K^{902}-G^{1203}	A^{1295}-S^{1627}	ACP1:S^{1672}-V^{1771}<br>ACP2:D^{1772}-L^{1845}	R^{1846}-G^{2151}	——
**Preu6**	M^{1}-V^{363}	D^{383}-P^{807}	S^{900}-S^{1197}	V^{1287}-T^{1596}	ACP1:D^{1648}-S^{1755}<br>ACP2:N^{1756}-S^{1819}	A^{1820}-A^{2205}	——
**Preu7**	M^{1}-P^{403}	T^{420}-P^{855}	P^{945}-P^{1256}	K^{1351}-A^{1676}	S^{1747}-D^{1811}	——	——
**Preu8**	M^{1}-R^{351}	N^{369}-G^{802}	P^{890}-G^{1186}	V^{1277}-P^{1602}	ACP1:S^{1655}-S^{1770}<br>ACP2:P^{1771}-L^{1849}	G^{1850}-L^{2158}	——

Table S2. Antifungal activities of compounds **1-8** (MIC, μg/mL)[a]

Strains	**1**	**2**	**3**	**4**	**5**	**6**	**7**	**8**	Ketoconazole
Verticillium dahliae Kleb	>100	>100	50	>100	>100	>100	50	>100	<0.78
Rhizoctonia solani	>100	>100	>100	>100	>100	>100	>100	>100	<0.78
Sclerotinia sclerotiorum	>100	>100	>100	>100	>100	>100	>100	>100	<0.78
Fusarium oxysporum f.sp. Vasinfectum	>100	>100	>100	>100	>100	>100	>100	>100	<0.78
Alternaria fragriae	>100	>100	>100	>100	>100	>100	>100	>100	<0.78
Helminthosporium maydis	>100	>100	25	>100	>100	>100	25	>100	<0.78
Botryosphaeria berengeriana	100	>100	25	>100	>100	>100	25	>100	<0.78

[a] MIC – minimum inhibitory concentration.

Table S3. Primers used in this study

Name	Sequence (5′→3′)	PCR product (Size in bp)	For construction of
Preu6*α*-BglII-F	GATGACAGGCGTGAGGTTCAACAAGATGGAG ATCTCAAAGTTAGCGAAATCGCTCTCG	Preu6*β*-1 (991 bp)	YEpPreu6*β*
Preu6*β*-R	TCATGTGGCCACCGCCGTGGATCATGAGCGCC GTTTGTATTCGACGGTCTTG		
Preu6*β*-F	TATCAAGTCAAGACCGTCGAATACAAACGGCG CTCATGATCCACGGCGGTG	Preu6*β*-2 (941 bp)	
Preu6*α*-3R	AGTGATGGTGATGGTGATGTCCGTTTAAACTC AAGCGTTCTGAATCAAAAAGTCAAGCC		
DrcA-1F	GACTACAAAGACGATGACGACAAGCTTCATAT GGCCTCTCCGTGGTCTTCTAACC	DrcA-1 (1,200 bp)	YEpDrcA-Δ(MT+TE)
DrcA-1R	GGAAGACTGGTCCGTTGGGATGCCGGGACTCG GTTCCAGTTCCGGGGCTCGAGTA		
DrcA-2F	AGTCCCGGCATCCCAACGGACCAGTCT	DrcA-2 (2,381 bp)	
DrcA-2R	TGCTCCAGCCGGTGCACCGCGTGATAG		
DrcA-3F	TTTCTATCACGCGGTGCACCGGCTGGAGCAGC GTCTCGGCTCATGCATCTGGCTC	DrcA-Δ(MT+TE)-3 (2,320 bp)	
DrcA-ACP-R	GATGGTGATGGTGATGTCCGTTTAAACCTATG CACCCACTGTGGAAAGCAAATCC		
AN7909-1F	GACTACAAAGACGATGACGACAAGCTTCATAT GGCTCCAAATCACGTTCT	AN7909-1 (984 bp)	YEpAN7909-ΔTE
AN7909-1R	ACTCTGGATTCCCGACGTCTCAG		
AN7909-2F	GCCTCAACTGAGACGTCGGGAATCCAGAGTCT CCTGGAGAAGAATGGCCT	AN7909-2 (2,277 bp)	
AN7909-2R	GCCATCCGACTTTAGGTATTC		
AN7909-3F	GCCCTCGACGAATACCTAAAGTCGGATGGCAA GGTGTCGACCACACGTCTGCGAG	AN7909-ΔTE-3 (2,385 bp)	
AN7909-ACPlinker-R	GATGGTGATGGTGATGTCCGTTTAAACCTACA GTTTGTGCTGGCGGCTGGAGTC		
Preu3-1F	GACTACAAAGACGATGACGACAAGCTTCATAT GAACCCTCCTAGCGCTTTGGCCTTTGG	Preu3-1 (2,522 bp)	YEpPreu3-Δ(MT+TE)
Preu3-1R	GCAATGGCCCGACAATTGGCTTCCACCGCATC AGTGCCATCTCCGGATATAAAC		
Preu3-2F	GGTGGAAGCCAATTGTCGGGCCATTGCCAAAT	Preu3-2 (2,554 bp)	
Preu3-2R	CGAATCAATGCCCAGATCGTCAAGCGT		
Preu3-3F	ACGCTTGACGATCTGGGCATTGATTCGCTTATG GTCATGGAGGTGCAAACTGAG	Preu3-Δ(MT+TE)-3 (405 bp)	
Preu3-ACPlinker-R	AGTGATGGTGATGGTGATGTCCGTTTAAACCT AAGGGTTAGGGGAGTCCAACGCG		
AN7909-BglII-R	ATTACGCGCTAGGGCAGCTGTTGACTG	Preu6*β*(ACP_{AN7909})-1 (235 bp)	YEpPreu6*β*(ACP_{AN7909})
AN7909-ACPlinker-F	CAACAGTCAACAGCTGCCCTAGCGCGTAATCG CGGCCGCTCCACCAAGCCGGTG	Preu6*β*(ACP_{AN7909})-2 (822 bp)	
DrcA-ACP-F	CAACAGTCAACAGCTGCCCTAGCGCGTAATGC TCTCAAGCAGGTTATGGCTGAAT	Preu6*β*(ACP_{DrcA})-2 (867 bp)	YEpPreu6*β*(ACP_{DrcA})
Preu3-ACPlinker-F	CAACAGTCAACAGCTGCCCTAGCGCGTAATCA GGAGCGGCCTAGCGGATCTCGC	Preu6*β*(ACP_{Preu3})-2 (672 bp)	YEpPreu6*β*(ACP_{Preu3})
Preu4-ACP-F	CAACAGTCAACAGCTGCCCTAGCGCGTAATTC GGGAATCATCAAGATTATACTGG	Preu6*β*(ACP_{Preu4})-2 (582 bp)	YEpPreu6*β*(ACP_{Preu4})
Preu4-ACP-R	GATGGTGATGGTGATGTCCGTTTAAACCTAAA GGGCCGCTTCAATGACCTCCAG		
Preu7-ACPlinker-F	CAACAGTCAACAGCTGCCCTAGCGCGTAATCC CGATGACTCCAGCCATTTGCAC	Preu6*β*(ACP_{Preu7})-2 (498 bp)	YEpPreu6*β*(ACP_{Preu7})
Preu7-ACPlinker-R	GATGGTGATGGTGATGTCCGTTTAAACCTACT AACTGTAATACTCCACCAGCC		
Preu8-ACP-F	CAACAGTCAACAGCTGCCCTAGCGCGTAATTC TCGTGTCATGAAGATCATTGC	Preu6*β*(ACP_{Preu8})-2 (645 bp)	YEpPreu6*β*(ACP_{Preu8})
Preu8-ACP-R	GATGGTGATGGTGATGTCCGTTTAAACCTACA ATTCGTTCTCGATGTCTTC		
DrcA-BglII-F	GTGTTGATTCGCTTTCCTTGATAGAGCTCAGAT CTTCAGTCCAGGAAGCATTCG	DrcA(H2663A)-1 (2,642 bp)	YEpDrcA(H2663A)
DrcA-H2663A-R	CCCCGGTTTCAATGTCAGATCAAAGAGAGCCC TTCTATGTGGCACCACTAGCAC		
DrcA-H2663A-F	GAGGTGCTAGTGGTGCCACATAGAAGGGCTCT CTTTGATCTGACATTGAAACCGG	DrcA(H2663A)-2 (177 bp)	
DrcA-TE-R	AGTGATGGTGATGGTGATGTCCGTTTAAACTC AAGTACTGTTGGCACCTGTAC		

Preu3-BglII-F	ATCAACCCGCGACACATCAAAATGCTACAAGATCTTGGCTTCTTACCGGTTTC	Preu3(H2496A)-1 (819 bp)	YEpPreu3(H2496A)
Preu3-H2496A-R	ATACTTATCAGACCAAACATCAAAACAGGCCTCCGCACCCTCAATAATCTCCAC		
Preu3-H2496A-F	AGAGTGGAGATTATTGAGGGTGCGGAGGCCTGTTTTGATGTTTGGTCTG	Preu3(H2496A)-2 (144 bp)	
Preu3-TE-R	AGTGATGGTGATGGTGATGTCCGTTTAAACCTAAGCATTCCGACACTGCTCCACCAAC		
Preu4-RruI-F	ACCGCCTACAACGTATATCATCTACGCTCGCGACGGTATGTGCAAGCACGAAAG	Preu4(H2128A)-1 (226 bp)	YEpPreu4(H2128A)
Preu4-H2128A-R	CTGCGGACCGGCTTGCATGATCGTGTAAGCATTAACGTTGCCCAAAACAGACACG		
Preu4-H2128A-F	CACGTGTCTGTTTTGGGCAACGTTAATGCTTACACGATCATGCAAGCCGGTC	Preu4(H2128A)-2 (132 bp)	
Preu4-TE-R	AGTGATGGTGATGGTGATGTCCGTTTAAACTCATCCCTGCAAAGCACGTG		
LtLasS2-RruI-F	CGAGGAAGCCGCCGCCGAGGTCGTGCTTCGCGACCACCCACAGGGGACGCGGTTC	LtLasS2(H2058A)-1 (1,825 bp)	YEpLtLasS2(H2058A)
LtLasS2-H2058A-R	CACATGCCCAGGCATCGGCATCTCCAAAGCATCCGCATCCACCGCCAAACACAAC		
LtLasS2-H2058A-F	CCGTTGTGTTTGGCGGTGGATGCGGATGCTTTGGAGATGCCGATGCCTGG	LtLasS2(H2058A)-2 (147 bp)	
LtLasS2-TE-R	GTGAAGGCATCGGTCCGCACATTTAAATCATCCGTTTAAACCGCCAGAC		
AtCurS2-Eco91I-F	ATTGACCTCCGGTATCAGGAACTCCCCCGGGTGACCTGGAAGCATGTTCTTG	AtCurS2(H2055A)-1 (1,479 bp)	YEpAtCurS2(H2055A)
AtCurS2-H2055A-R	CACATGTCCTGGCATGGGCAAATCCAAAGCATCCCCAGCCACCGACAACGC		
AtCurS2-H2055A-F	GTGTTGGCGTTGTCGGTGGCTGGGGATGCTTTGGATTTGCCCATGCCAGGAC	AtCurS2(H2055A)-2 (147 bp)	
AtCurS2-TE-R	AGTGATGGTGATGGTGATGTCCGTTTAAACTTAGGTAGACTCCCCCTCAAAATATGC		
pUSA-Preu6*β*-1F	CACAGCAAGCTCCGAATTCGAGCTCGGTACATGTCTAATTCTACACGTGACTATC	pUSA-Preu6*β*-1 (2,214 bp)	pUSA-Preu6*β*
pUSA-Preu6*β*-1R	ATGGCCAATGTTTCCTTTCACAGAGCCTAG		
pUSA-Preu6*β*-2F	CTAGGCTCTGTGAAAGGAAACATTGGCCATTGCGAAACTGGCGCTGGCGTCGCTG	pUSA-Preu6*β*-2 (2,230 bp)	
pUSA-Preu6*β*-2R	GGATGATCGACGAAATCGGCTTGAG		
pUSA-Preu6*β*-3F	AATTCCTCAAGCCGATTTCGTCGATCATCCTGCGCGAAAACGAATCCTTGGC	pUSA-Preu6*β*-3 (2,224 bp)	
pUSA-Preu6*β*-3R	CACCCTTCACGAGCTACTACAGATCCCCGGTCAAGCGTTCTGAATCAAAAAGTC		
Preu6*β*-S1681A-1F	GATGACAGGCGTGAGGTTCAACAAGATGGAGATCTCAAAGTTAGCGAAATCGCTC	Preu6*β*(S1681A)-1 (349 bp)	YEpPreu6*β*(S1681A)
Preu6*β*-S1681A-1R	AACGGAAGAGAGCGCGTCAAGACCTAAGTCGACG		
Preu6*β*-S1681A-2F	TTAGGTCTTGACGCGCTCTCTTCCGTTGAGTTCGC	Preu6*β*(S1681A)-2 (788 bp)	
Preu6*β*-S1681A-2R	GATGGGTCCATCGATGAGATTGACCTCCGGACACAGACGGTAGTCAATACTAATG		
Preu6*β*-S1790A-1R	GACGGATGACAAGGCATCCAGTCCAAGATCAATAAG	Preu6*β*(S1790A)-1 (676 bp)	YEpPreu6*β*(S1790A)
Preu6*β*-S1790A-2F	CTTGGACTGGATGCCTTGTCATCCGTCGAGTTTGCG	Preu6*β*(S1790A)-2 (1,223 bp)	
Preu6*β*-C9his-1F	GATGACAGGCGTGAGGTTCAACAAGATGGAGATCTCAAAGTTAGCGAAATCGCTC	Preu6*β*-C9his-1 (1,030 bp)	YEpPreu6*β*(C9His)
Preu6*β*-C9his-1R	TCCGAGACAAGGTCATGTGGCCACCGCCGTGGATCAGTGATGATGATGATGATGATGATGATGTGAGCGCCGTTTGTATTCGAC		
Preu6*β*-C9his-2F	GTCAAGACCGTCGAATACAAACGGCGCTCACATCATCATCATCATCATCATCATCACTGATCCACGGCGGTGGCCACATGACCTTGTC	Preu6*β*-C9his-2 (966 bp)	
Preu6*β*-C9his-2R	AATCAGTGATGGTGATGGTGATGTCCGTTTAAACTCAAGCGTTCTGAATCAAAAAG		
Preu6-F	TATGCTACGGGAGCCTCTAC	Preu6*α1* (232 bp) Preu6*β* (162 bp)	RT-PCR analysis
Preu6-R1	CCTTCCGAGACAAGGTCATGTGG		
Preu6-R2	AGACGCGTTGAACAAGAGGG	Preu6*α2* (172 bp)	
β-actin-F	CATGAAGTGCGACGTCGATGTC	*β-actin* (151 bp)	
β-actin-R	GGGAGCAATGATCTTGACCTTC		

Table S4. Plasmids used in this study

**Plasmids**	**Description**	**Source/Ref.**
YEpADH2p-FLAG-URA	*URA3*, *ADH2p*, *ADH2t*, *AmpR*	1
YEpADH2p-FLAG-TRP	*TRP1*, *ADH2p*, *ADH2t*, *AmpR*	1
pUSA	*sC*, *PamyB*, *TamyB*, *AmpR*	12
YEpPreu6*α*	a 6,618 bp fragment of *preu6α* (SAT-KS-AT-PT-ACP-ACP-TE) was inserted in YEpADH2p-FLAG-URA	2
YEpPreu3	a 7,572 bp fragment of *preu3* (SAT-KS-AT-PT-ACP-MT-TE) was inserted in YEpADH2p-FLAG-URA	2
YEpPreu6*α*(H2177A)	a 6,618 bp fragment of *preu6α* (His2177 to Ala) was inserted in YEpADH2p-FLAG-URA	3
YEpPreu4	a 6,456 bp fragment of *preu4* (SAT-KS-AT-PT-ACP-ACP-TE) was inserted in YEpADH2p-FLAG-URA	4
YEpPreu7	a 5,466 bp fragment of *preu7* (SAT-KS-AT-PT-ACP) was inserted in YEpADH2p-FLAG-URA	5
YEpPreu8	a 6,477 bp fragment of *preu8* (SAT-KS-AT-PT-ACP-ACP-TE) was inserted in YEpADH2p-FLAG-URA	6
YEpLtLasS1	a 7,173 bp fragment of *ltlasS1* (KS-AT-DH-ER-KR-ACP) was inserted in YEpADH2p-FLAG-TRP	7
YEpLtLasS2	a 6,252 bp fragment of *ltlasS2* (SAT-KS-AT-PT-ACP-TE) was inserted in YEpADH2p-FLAG-URA	7
YEpAtCurS1	a 7,164 bp fragment of *atcurS1* (KS-AT-DH-ER-KR-ACP) was inserted in YEpADH2p-FLAG-TRP	8
YEpAtCurS2	a 6,252 bp fragment of *atcurS2* (SAT-KS-AT-PT-ACP-TE) was inserted in YEpADH2p-FLAG-URA	8
pUSA(Preu6*α*)	a 6,618 bp fragment of *preu6α* (SAT-KS-AT-PT-ACP-ACP-TE) was inserted in pUSA	12
YEpPreu6*β*	A 6,548 bp *preu6α* fragment (SAT-KS-AT-PT-ACP-ACP-TE) with a 70 bp absence of intron i_3-1 (5,668-5,737 bp) , resulting in the generation of a PTC, and the actually translatable fragment is a 5,676 bp *preu6β* (SAT-KS-AT-PT-ACP-ACP), which was inserted into YEpADH2p-FLAG-URA	This study
YEpDrcA-Δ(MT+TE)	a 5,784 bp fragment of *drcA* (SAT-KS-AT-PT-ACP-ACP-ACP) was inserted in YEpADH2p-FLAG-URA	This study
YEpAN7909-ΔTE	a 5,529 bp fragment of *AN7909* (SAT-KS-AT-PT-ACP-ACP) was inserted in YEpADH2p-FLAG-URA	This study
YEpPreu3-Δ(MT+TE)	a 5,367 bp fragment of *preu3* (SAT-KS-AT-PT-ACP) was inserted in YEpADH2p-FLAG-URA	This study
YEpPreu6*β*(ACP_{AN7909})	a 4,941 bp fragment of *preu6β* (SAT-KS-AT-PT) and a 765 bp fragment of *AN7909* (ACP-ACP) were inserted in YEpADH2p-FLAG-URA	This study
YEpPreu6*β*(ACP_{DrcA})	a 4,941 bp fragment of *preu6β* (SAT-KS-AT-PT) and an 810 bp fragment of *drcA* (ACP-ACP-ACP) were inserted in YEpADH2p-FLAG-URA	This study
YEpPreu6*β*(ACP_{Preu3})	a 4,941 bp fragment of *preu6β* (SAT-KS-AT-PT) and a 612 bp fragment of *preu3* (ACP) were inserted in YEpADH2p-FLAG-URA	This study

YEpPreu6*β*(ACP_{Preu4})	a 4,941 bp fragment of *preu6β* (SAT-KS-AT-PT) and a 525 bp fragment of *preu4* (ACP-ACP) were inserted in YEpADH2p-FLAG-URA	This study
YEpPreu6*β*(ACP_{Preu7})	a 4,941 bp fragment of *preu6β* (SAT-KS-AT-PT) and a 438 bp fragment of *preu7* (ACP) were inserted in YEpADH2p-FLAG-URA	This study
YEpPreu6*β*(ACP_{Preu8})	a 4,941 bp fragment of *preu6β* (SAT-KS-AT-PT) and a 588 bp fragment of *preu8* (ACP-ACP) were inserted in YEpADH2p-FLAG-URA	This study
YEpDrcA(H2663A)	an 8,106 bp fragment of *DrcA* (His2663 to Ala) was inserted in YEpADH2p-FLAG-URA	This study
YEpPreu3(H2496A)	a 7,572 bp fragment of *Preu3* (His2496 to Ala) was inserted in YEpADH2p-FLAG-URA	This study
YEpPreu4(H2128A)	a 6,456 bp fragment of *preu4* (His2128 to Ala) was inserted in YEpADH2p-FLAG-URA	This study
YEpLtLasS2(H2058A)	a 6,252 bp fragment of *ltlasS2* (His2058 to Ala) was inserted in YEpADH2p-FLAG-URA	This study
YEpAtCurS2(H2055A)	a 6,252 bp fragment of *atcurS2* (His2055 to Ala) was inserted in YEpADH2p-FLAG-URA	This study
pUSA(Preu6*β)*	a 5,676 bp fragment of *preu6β* (SAT-KS-AT-PT-ACP-ACP) was inserted in pUSA	This study
YEpPreu6*β*(S1681A)	a 5,676 bp fragment of *preu6β* (Ser1681 to Ala) was inserted in YEpADH2p-FLAG-URA	This study
YEpPreu6*β*(S1790A)	a 5,676 bp fragment of *preu6β* (Ser1790 to Ala) was inserted in YEpADH2p-FLAG-URA	This study
YEpPreu6*β*(S1681,1790A)	a 5,676 bp fragment of *preu6β* (Ser1681, 1790 to Ala) was inserted in YEpADH2p-FLAG-URA	This study
YEpPreu6*β*(C9His)	a 5,676 bp fragment of *preu6β* (SAT-KS-AT-PT-ACP-ACP) and a 27 bp fragment of 9×His (CATCATCATCATCATCATCATCATCAC) were inserted in YEpADH2p-FLAG-URA	This study

Table S5. The HRESIMS data analysis of compounds in this study

Compounds	Molecular formula	Calculated molecular weight	Measured molecular weight	Mass error value(ppm)
**9**	$[M+H]^+$: $C_{27}H_{35}N_2O_{10}S$	579.2012	579.20056	-1.10
**10**	$[M+H]^+$: $C_{16}H_{17}O_5$	289.1076	289.10690	-2.42
**11**	$[M+H]^+$: $C_{10}H_{13}O_4$	197.0814	197.08089	-2.59
**12**	$[M+H]^+$: $C_{19}H_{29}N_2O_7S$	429.1695	429.16925	-0.58
orsellinic acid	$[M-H]^-$: $C_8H_7O_4$	167.0344	167.03461	+1.26
lecanoric acid	$[M-H]^-$: $C_{16}H_{13}O_7$	317.0661	317.06793	+5.77
prephenalenone	$[M+H]^+$: $C_{14}H_{13}O_6$	277.0712	277.07062	-2.09
atrochrysone	$[M+H]^+$: $C_{15}H_{15}O_5$	275.0919	275.09131	-2.14
desmethyl-lasiodiplodin	$[M+H]^+$: $C_{16}H_{23}O_4$	279.1596	279.15903	-2.04
10,11-dehydrocurvularin	$[M+H]^+$: $C_{16}H_{19}O_5$	291.1232	291.12256	-2.20
3-methylorsellinic acid	$[M-H]^-$: $C_9H_9O_4$	181.0501	181.05054	+2.43
3-methylorsellinic acid-PANT	$[M-H]^-$: $C_{20}H_{29}N_2O_7S$	441.1695	441.17188	+5.39
isonorobtusatic acid	$[M-H]^-$: $C_{17}H_{15}O_7$	331.0818	331.08374	+5.86
isonorobtusatic acid-PANT	$[M-H]^-$: $C_{28}H_{35}N_2O_{10}S$	591.2012	591.20465	+5.84
norobtusatic acid	$[M-H]^-$: $C_{17}H_{15}O_7$	331.0818	331.08362	+5.50
norobtusatic acid-PANT	$[M-H]^-$: $C_{28}H_{35}N_2O_{10}S$	591.2012	591.20447	+5.53
4-*O*-demethylbarbatic acid	$[M-H]^-$: $C_{18}H_{17}O_7$	345.0974	345.09930	+5.51
4-*O*-demethylbarbatic acid-PANT	$[M-H]^-$: $C_{29}H_{37}N_2O_{10}S$	605.2169	605.22034	+5.68
CJ-20,557	$[M-H]^-$: $C_{19}H_{19}O_7$	359.1131	359.11505	+5.43
CJ-20,557-PANT	$[M-H]^-$: $C_{30}H_{39}N_2O_{10}S$	619.2325	619.23596	+5.59

Table S6. ^{1}H NMR and ^{13}C NMR data

Table S6.1. Compound **9** (in methanol-d_4)

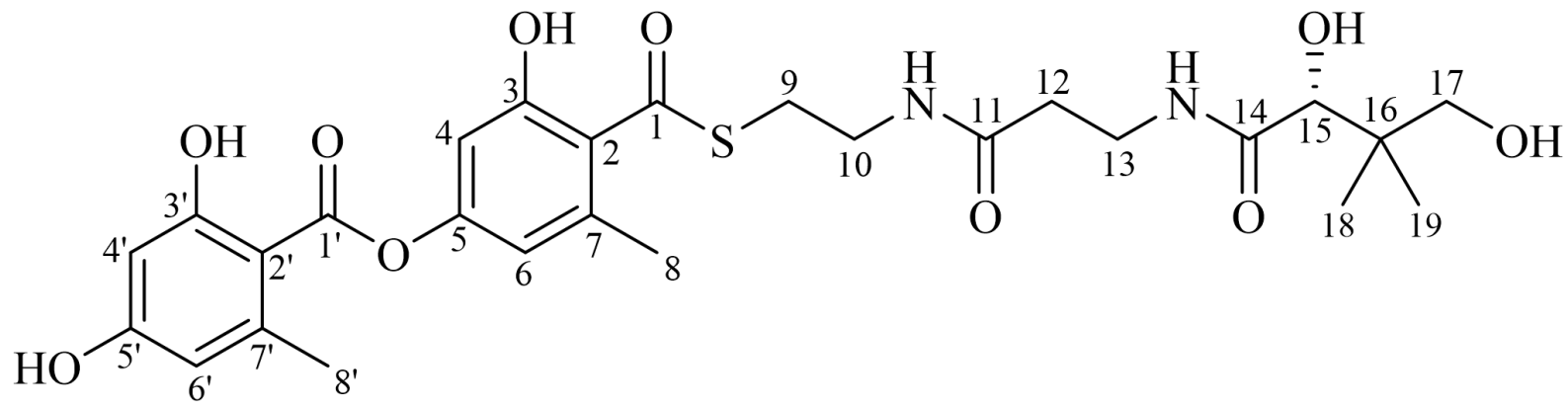

No.	δC, type	δH (m, J_{HH}, area)
1	196.6, C	
2	127.1, C	
3	156.3, C	
4	108.2, CH	6.57 (d, J = 1.7, 1H)
5	152.9, C	
6	115.6, CH	6.59 (d, J = 1.4, 1H)
7	138.3, C	
8	19.2, CH_3	2.29 (s, 3H)
9	29.9, CH_2	3.21 (t, J = 6.6, 2H)
10	40.0, CH_2	3.47 (overlap, 2H)
11	174.0, C	
12	36.4, CH_2	2.45 (t, J =6.6, 2H)
13	36.3, CH_2	3.49 (overlap, 2H)
14	176.1, C	
15	77.3, CH	3.89 (s, 1H)
16	40.4, C	
17	70.3, CH_2	3.45 (overlap, 1H), 3.39 (d, J =11.0, 1H)
18	21.3, CH_3	0.92 (s, 3H)
19	20.9, CH_3	0.92 (s, 3H)
1'	171.0, C	
2'	105.3, C	
3'	166.5, C	
4'	101.9, CH	6.22 (d, J = 2.3, 1H)
5'	164.5, C	
6'	112.9, CH	6.29 (d, J = 2.4, 1H)
7'	144.7, C	
8'	24.3, CH_3	2.56 (s, 3H)

^{1}H and ^{13}C NMR spectra were recorded at 600 and 150 MHz, respectively.

Table S6.2. Compound **10** (in methanol-d_4)

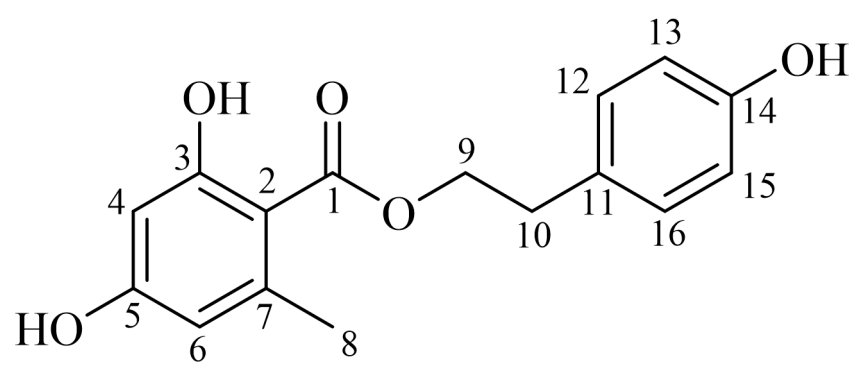

No.	δC, type	δH (m, J_{HH}, area)
1	171.6, C	
2	105.4, C	
3	166.6, C	
4	101.9, CH	6.22 (d, J = 2.5, 1H)
5	164.5, C	
6	112.9, CH	6.29 (d, J = 2.5, 1H)
7	144.8, C	
8	24.4, CH_3	2.58 (s, 3H)
9	64.1, CH_2	3.77 (t, J = 6.9, 2H)
10	39.6, CH_2	2.85 (t, J = 6.9, 2H)
11	138.5, C	
12	131.1, CH	7.31 (d, J = 8.3, 1H)
13	122.8, CH	7.12 (d, J = 8.3, 1H)
14	150.0, C	
15	122.8, CH	7.12 (d, J = 8.3, 1H)
16	131.1, CH	7.31 (d, J = 8.3, 1H)

^{1}H and ^{13}C NMR spectra were recorded at 600 and 150 MHz, respectively.

Table S7. The amino acid sequences of Preu6α and Preu6β

Protein	Amino acid sequence
Preu6α (OK493441)	MSNSTRDYPISAAFFCPQSRAPPAEYLHALYSFLSQNTLGKAFLRHIASLDEVWPIFSEAR<br>DDILRLPDARQNINVLVDWAKGGSSTPIAEARSGVIALPSVFIVQLGQYFRYLEANRLSH<br>GDFIGQLKDIGGVHGYCGGAAAALSVACAADETQLIDHAAVLLRLFVGIGCCIEAVDD<br>WTTTESTVIACRLKYEGQGDELCSRFPEPKSISITGNARTLSELFDYAVGLGLPTHKMEIT<br>GKAHNPENAELAKDFINLYRRTPALQLPPTFKLQATVRSNRTAEKLTNEGIIEDMITMIIA<br>SQCDWNTLLTRVAEDMKVSGRPFHKMVSFGMNDCVPVTPFNRQRLKTTKFEAHVLIEP<br>LKPSRISAAQYPTFSDDAIAITGASLRLPGANNLDELWDLISKGTDCHREIPKDRFDPHNI<br>YRTSQSGFSKAQKYFGNFLEDIKGFDRAYFSMGVREAANIDPQQRLLLELAVEALEASG<br>YLANHVREAGDPVGCFVGASFIEYLENTGAHPPTAYTAPGTIRAFLCGRLSYYFGWTAP<br>AEVIDTACSASMVAINRAVKSIQAGECEMALAGGVNLITGMNNYLDLAKAGFLSPTGQ<br>CKPFDQSGDGYCRSDGAGFVVLKKLSQALVNGDPIMGVIPSIETNQGGLSGSLTVPSST<br>ALQALYKRVLSKSGLEPAQITYVEAHGTGTQAGDPIEVESVRAVLGDPTRAHSLSLGSV<br>KGNIGHCETGAGVAGLLKVLAMIKHGGIPPLASHKALNPKIPALETHHMEIAKQLKPW<br>DVPLRAAFVNSYGAAGSNAAVICVEPPPVVTDGSSLIGTEPQKVTLPVIVSGATRKSLVL<br>NARALASYLSQDGSHLSIHDVAFTVNQRRKRNRFCAEVSGTDLPSLVQSLRAVDSPSFE<br>SPGKSKPVVLVFSGQNTNAVALDRTIYDTYPVFKAYIDACDSEIVKLGFPSIMEAIFQKE<br>PISTAVALQGSIFAMQYACARSWIDAGLKPRAIIGHSFGELTALAVSGALSLADSLKLVT<br>CRGHLIDTKWGEERGGMLVIHADVATVERFQSRFKAQHDGAELEIACYNSPTTTVVAG<br>PVAYMDAAEQMLATDPDFQGLRKLRIVTSNAFHSSLSDPILADLDSMADTLTWNEPSIP<br>LEACTSEGLASIKEWSASRHTRGSVYFTKAVERIEGRLGACIWVEAGLDSAIIAMARKA<br>SSKPDSQVFQSVSTKAGATSFIDGIVNNLWRQGVPLSHLNALSATVKPNPVWLPPYQFE<br>REQHWTEHIDRATEASQASTTSDTIQSTPTQTVQSPPKLISRLASLQYQINTQCERFQKIT<br>EGHAVLYEPLCPASLYMECVVMALQELAGDLGSRTLDFENLDFHAGLGLQTDRRVLL<br>DLEEARPHSWTFKVQSTKAGSSRSLLHCSGRVILTESSVPTTFQRLVDGPRSRLDQDKD<br>AEKLMSSRAYGLFSNIMTYSEFLKPISSIILRENESLATIKLPPNQPGLHESTAWKRCDAV<br>FLDGFISSSGLLLNSSSVVQSGHVLIAVGVERAILTAAFQASLASSWQAYATFTMVGETH<br>ALCDVFACTPDGEVVAMMTGVRFNKMEISKLAKSLSSVNASSPTGGRTQPPAAPKTQA<br>QPMASRPSPTPLQVSFATAEPAAPEPVQQSTAALARNDIGPVLKSLISNYTGLIEEDVSED<br>SPLVDLGLDSLSSVEFASEIGTKFGVTLDADTVGDLTLHSLCQRLSGTSNVVSQKMSETP<br>AAAPVKELIETVPSPIVTFSSPVSNSITSVLKSLLGSYTGLQEEDMPDDVPLIDLGLDSLSS<br>VEFASELNDKMGADIDSAVVADMTLSALEQQLGASATPPSTTGSSTPGDISTAATTPYA<br>TGASTPDYLVHGNKPSISNGVVAAKDSYQVKTVEYKRVSGVPIHADIYVPLVQRVSPM<br>PLALMIHGGGHMTLSRKAVRPTQAKYLLSHGFLPISIDYRLCPEVNLIDGPIADVRDAYV<br>WACQNLGTHLAEHSISVDGGRVVVVGWSTGGHLAMSLGWSLEEAGVPPPKAVLSFYA<br>PVDFESGELDNQKNPALPKPRMTLDQITKALPRTPVTQYGASSTDETNLGWLHPGDPRS<br>ELLLHVFHSDIGLPLILHGLPISGSGRPSPSLVASISPLARLRNGSYTIPTFIIHGTKDVIAPY<br>AAAERFVKIMSEKGVKSGFLSLSGTGHVFDVTMKPDSKGWEDKVKPGLDFLIQNA*

Preu6β	MSNSTRDYPISAAFFCPQSRAPPAEYLHALYSFLSQNTLGKAFLRHIASLDEVWPIFSEAR<br>DDILRLPDARQNINVLVDWAKGGSSTPIAEARSGVIALPSVFIVQLGQYFRYLEANRLSH<br>GDFIGQLKDIGGVHGYCGGAAAALSVACAADETQLIDHAAVLLRLFVGIGCCIEAVDD<br>WTTTESTVIACRLKYEGQGDELCSRFPEPKSISITGNARTLSELFDYAVGLGLPTHKMEIT<br>GKAHNPENAELAKDFINLYRRTPALQLPPTFKLQATVRSNRTAEKLTNEGIIEDMITMIIA<br>SQCDWNTLLTRVAEDMKVSGRPFHKMVSFGMNDCVPVTPFNRQRLKTTKFEAHVLIEP<br>LKPSRISAAQYPTFSDDAIAITGASLRLPGANNLDELWDLISKGTDCHREIPKDRFDPHNI<br>YRTSQSGFSKAQKYFGNFLEDIKGFDRAYFSMGVREAANIDPQQRLLLELAVEALEASG<br>YLANHVREAGDPVGCFVGASFIEYLENTGAHPPTAYTAPGTIRAFLCGRLSYYFGWTAP<br>AEVIDTACSASMVAINRAVKSIQAGECEMALAGGVNLITGMNNYLDLAKAGFLSPTGQ<br>CKPFDQSGDGYCRSDGAGFVVLKKLSQALVNGDPIMGVIPSIETNQGGLSGSLTVPSST<br>ALQALYKRVLSKSGLEPAQITYVEAHGTGTQAGDPIEVESVRAVLGDPTRAHSLSLGSV<br>KGNIGHCETGAGVAGLLKVLAMIKHGGIPPLASHKALNPKIPALETHHMEIAKQLKPW<br>DVPLRAAFVNSYGAAGSNAAVICVEPPPVVTDGSSLIGTEPQKVTLPVIVSGATRKSLVL<br>NARALASYLSQDGSHLSIHDVAFTVNQRRKRNRFCAEVSGTDLPSLVQSLRAVDSPSFE<br>SPGKSKPVVLVFSGQNTNAVALDRTIYDTYPVFKAYIDACDSEIVKLGFPSIMEAIFQKE<br>PISTAVALQGSIFAMQYACARSWIDAGLKPRAIIGHSFGELTALAVSGALSLADSLKLVT<br>CRGHLIDTKWGEERGGMLVIHADVATVERFQSRFKAQHDGAELEIACYNSPTTTVVAG<br>PVAYMDAAEQMLATDPDFQGLRKLRIVTSNAFHSSLSDPILADLDSMADTLTWNEPSIP<br>LEACTSEGLASIKEWSASRHTRGSVYFTKAVERIEGRLGACIWVEAGLDSAIIAMARKA<br>SSKPDSQVFQSVSTKAGATSFIDGIVNNLWRQGVPLSHLNALSATVKPNPVWLPPYQFE<br>REQHWTEHIDRATEASQASTTSDTIQSTPTQTVQSPPKLISRLASLQYQINTQCERFQKIT<br>EGHAVLYEPLCPASLYMECVVMALQELAGDLGSRTLDFENLDFHAGLGLQTDRRVLL<br>DLEEARPHSWTFKVQSTKAGSSRSLLHCSGRVILTESSVPTTFQRLVDGPRSRLDQDKD<br>AEKLMSSRAYGLFSNIMTYSEFLKPISSIILRENESLATIKLPPNQPGLHESTAWKRCDAV<br>FLDGFISSSGLLLNSSSVVQSGHVLIAVGVERAILTAAFQASLASSWQAYATFTMVGETH<br>ALCDVFACTPDGEVVAMMTGVRFNKMEISKLAKSLSSVNASSPTGGRTQPPAAPKTQA<br>QPMASRPSPTPLQVSFATAEPAAPEPVQQSTAALARNDIGPVLKSLISNYTGLIEEDVSED<br>SPLVDLGLDSLSSVEFASEIGTKFGVTLDADTVGDLTLHSLCQRLSGTSNVVSQKMSETP<br>AAAPVKELIETVPSPIVTFSSPVSNSITSVLKSLLGSYTGLQEEDMPDDVPLIDLGLDSLSS<br>VEFASELNDKMGADIDSAVVADMTLSALEQQLGASATPPSTTGSSTPGDISTAATTPYA<br>TGASTPDYLVHGNKPSISNGVVAAKDSYQVKTVEYKRRS*

3 SI Figures

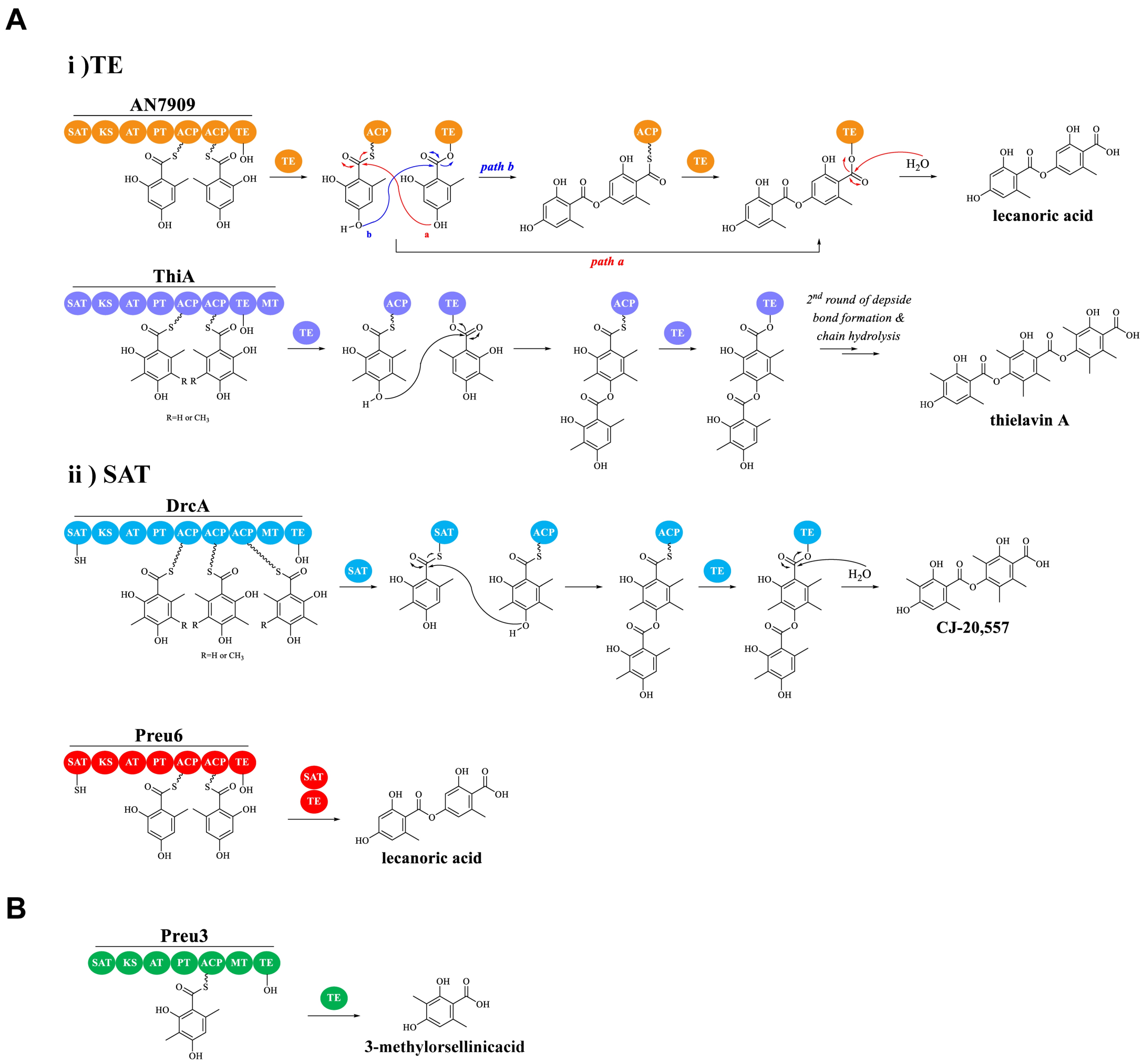

Figure S1. Biosynthetic models for the OA derivatives.

A. **i**) TE domain-catalyzed depside bond formation reactions. Reactions catalyzed by AN7909 and ThiA; **ii**) SAT domain-catalyzed depside bond formation reactions. Reactions catalyzed by DrcA and Preu6. B. OA monomer derivative (3-methyl OA) related nrPKS, Preu3.

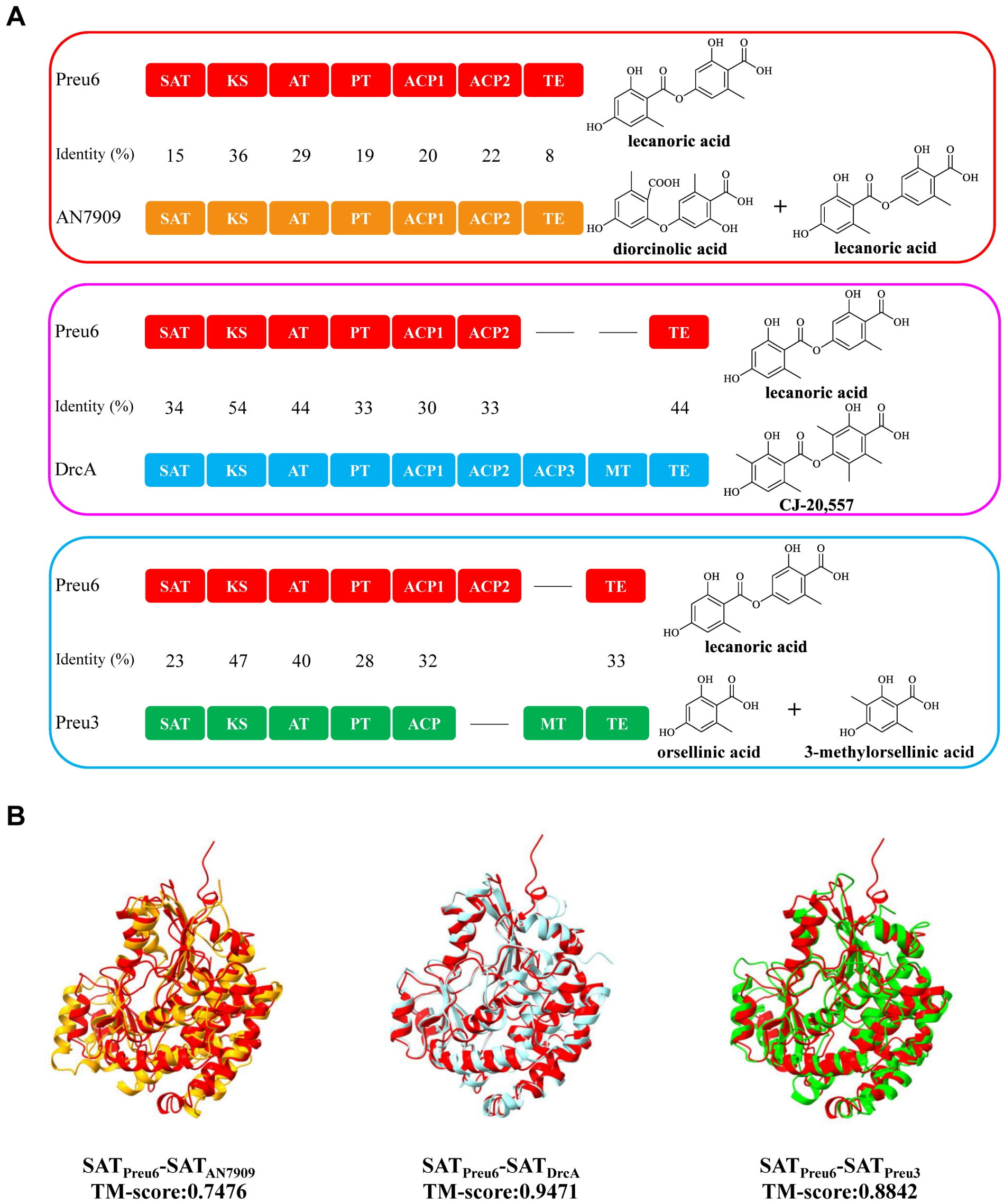

Figure S2. Comparisons between Preu6 and other OA-related nrPKSs.

A. Percent identities between the indicated protein domains conducted at BLASTP (https://blast.ncbi.nlm.nih.gov/Blast.cgi), and the main products of the enzymes were also indicated. B. 3D structural comparison of the SAT domains. Notably, although the sequence identity between the SAT domains of DrcA and Preu6 is only 34%, the TM-score of 0.9471 reflects a high degree of structural similarity between these two domains, which is due to the TM-score being based on the 3D structure rather than sequence identity, highlighting that the domains share a similar fold despite differences in their primary sequences.

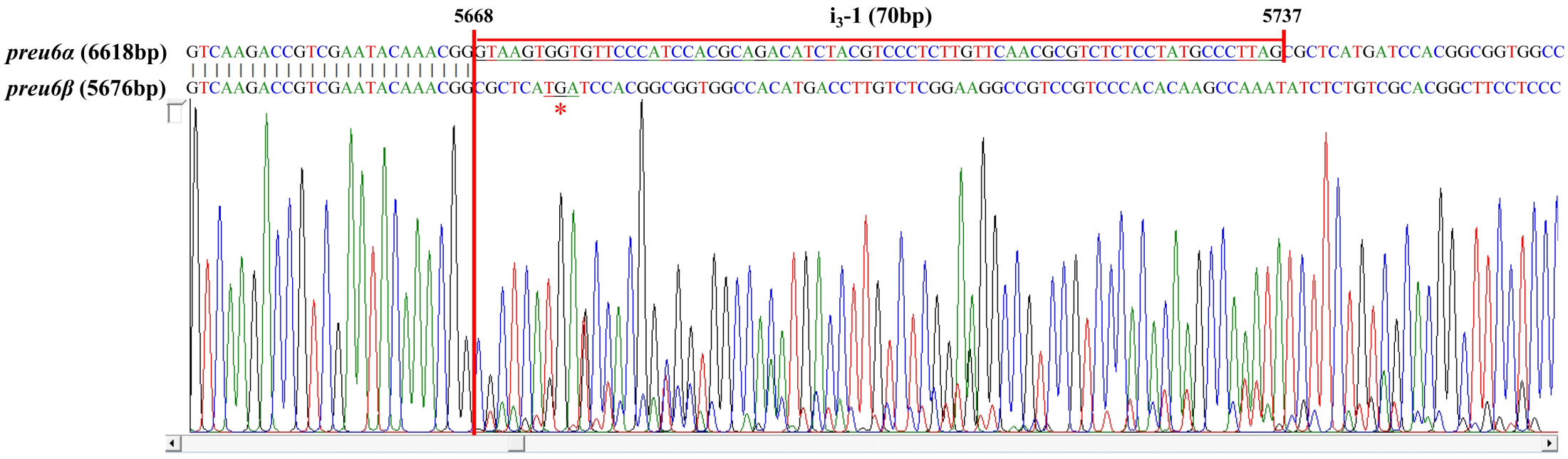

Figure S3. Sequence analysis of the *preu6α/β* transcripts.
*, indicates the premature termination codon.

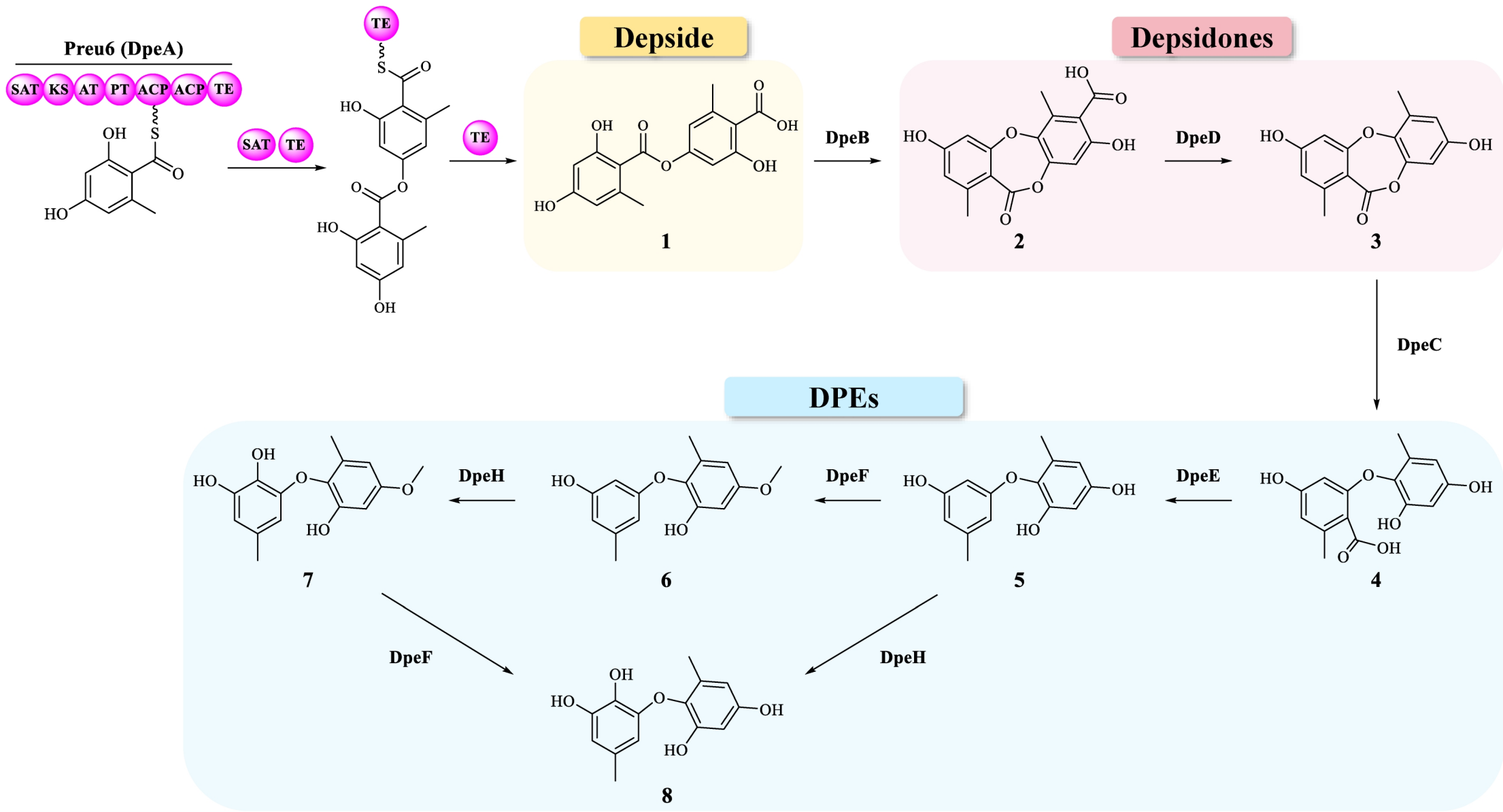

Figure S4. Biosynthetic model for DPEs **1-8**.

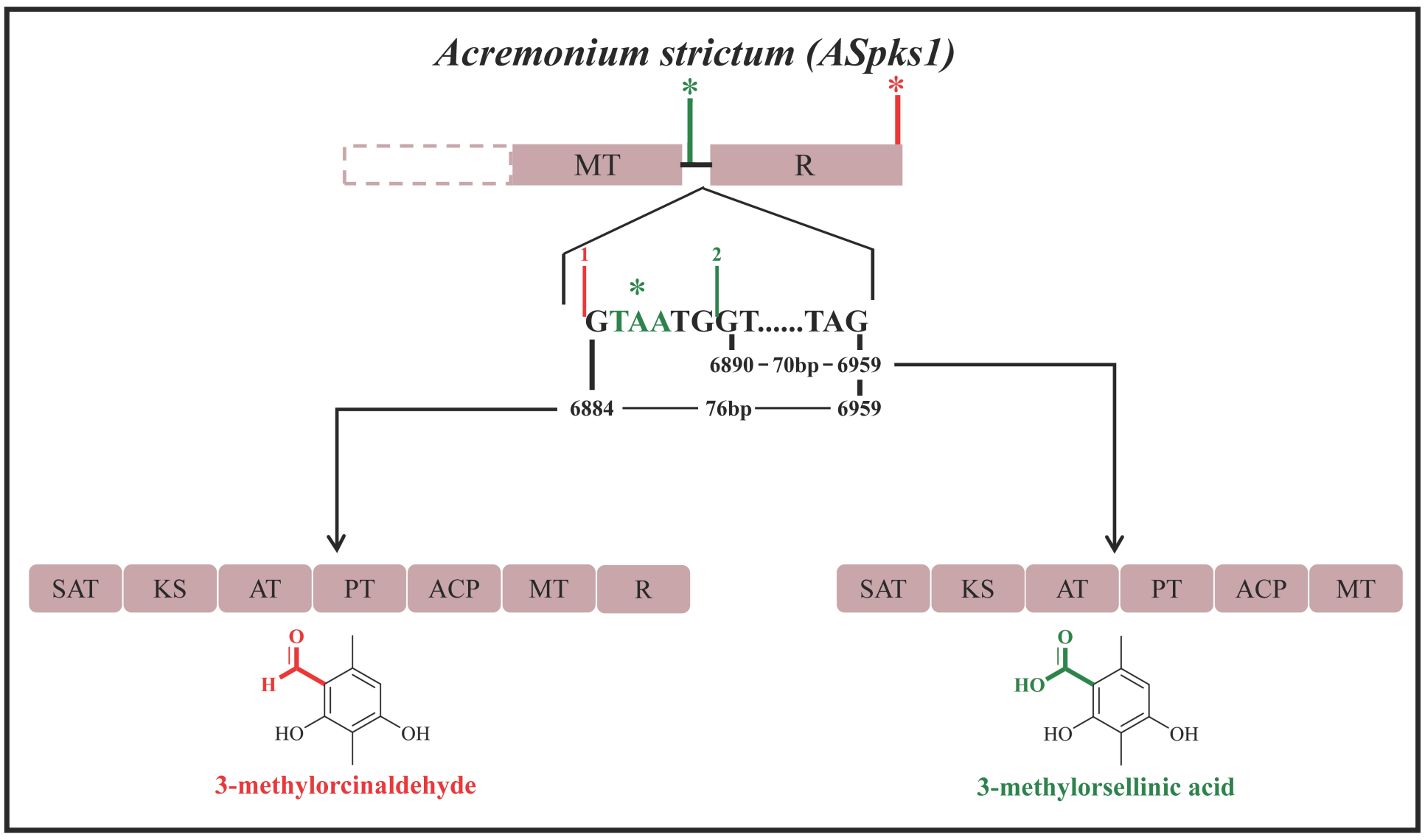

Figure S5. Alternative splicing (AS) event in *ASpks1* gene.

*, indicates the premature termination codon in green and the termination condon in red. 1, indicates regular splice site; 2, indicates alternative splice site.

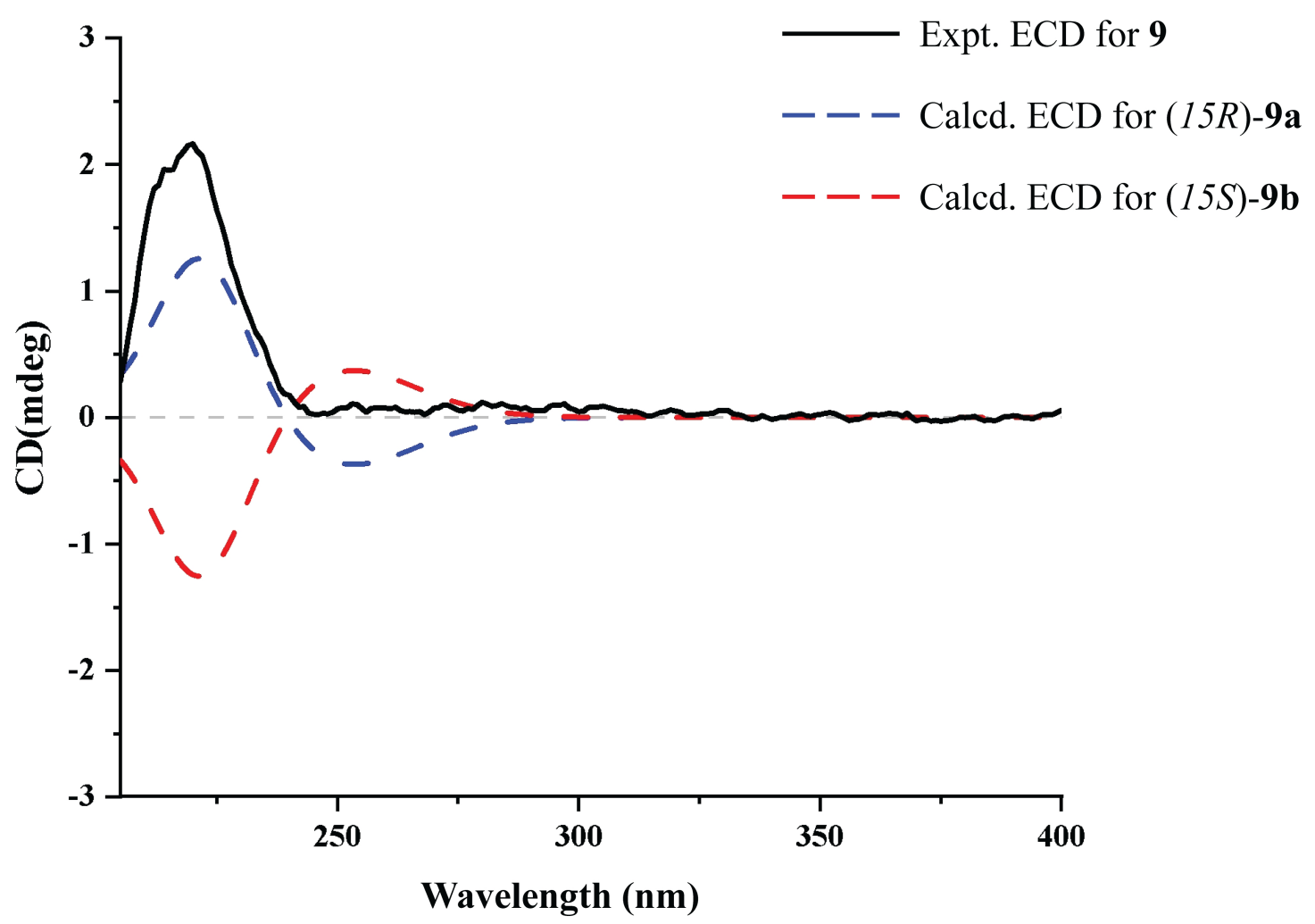

Figure S6. Experimental ECD spectra of compound **9** in MeOH.

The experimental ECD spectrum of **9** (black solid line) was compared with the ECD spectra of the two possible stereoisomers, (15*R*)-**9a** (blue dashed line) and (15*S*)-**9b** (red dashed line).

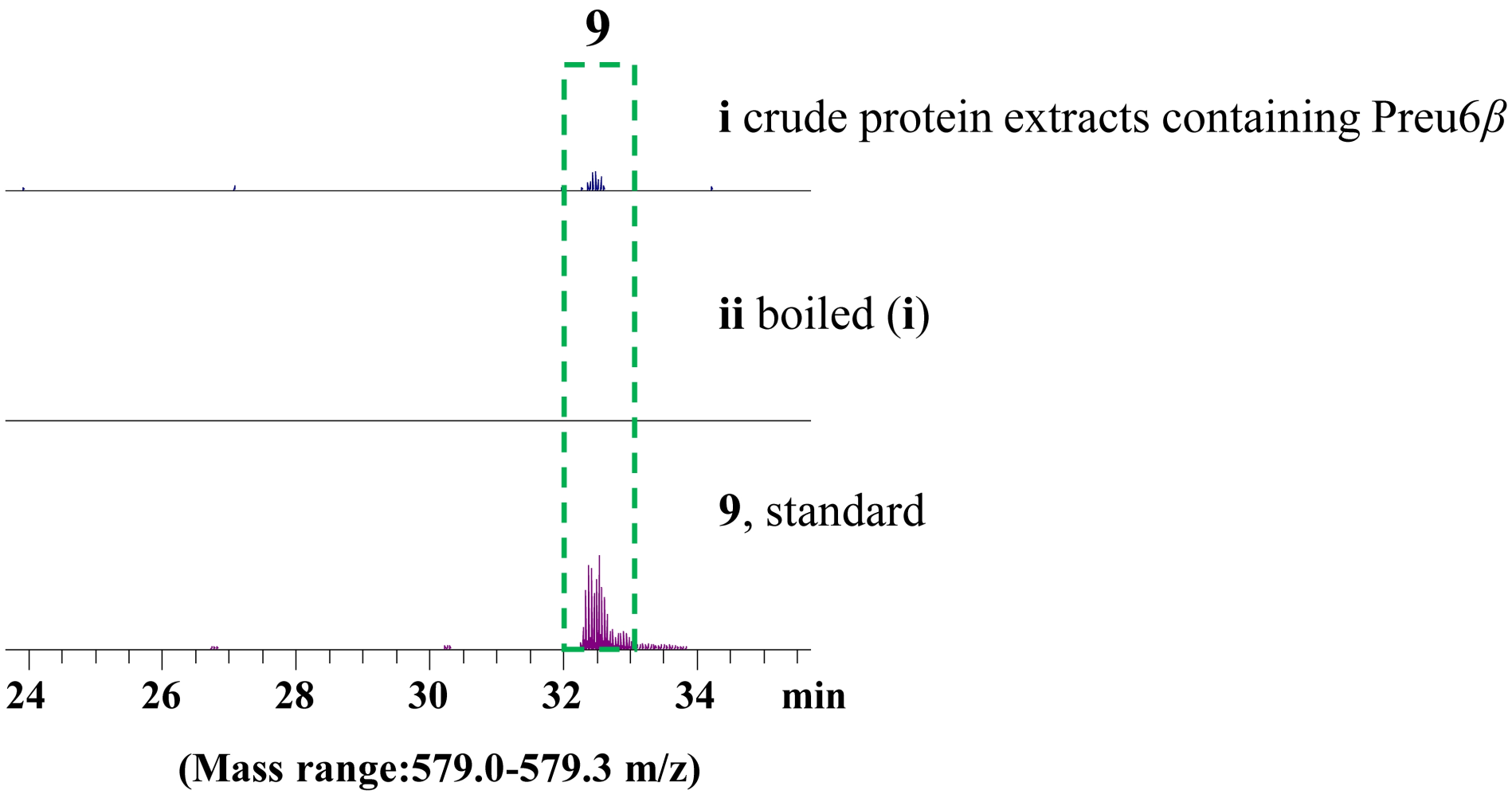

Figure S7. Confirmation of the catalytic activity of Preu6*β*.

In vitro assays showed that the crude protein extracts of *S. cerevisiae* BJ5464-NpgA expressing Preu6*β* is able to produce **9** from acetyl-CoA, malonyl-CoA, and NADPH after 20 h of incubation. The extracted ion chromatograms (EICs) were extracted at *m/z* 579.0-579.3 $[M+H]^+$ for **9**. **i**, crude protein extracts containing Preu6*β*. **ii**, boiled control of **i**. bottom, standard of **9**.

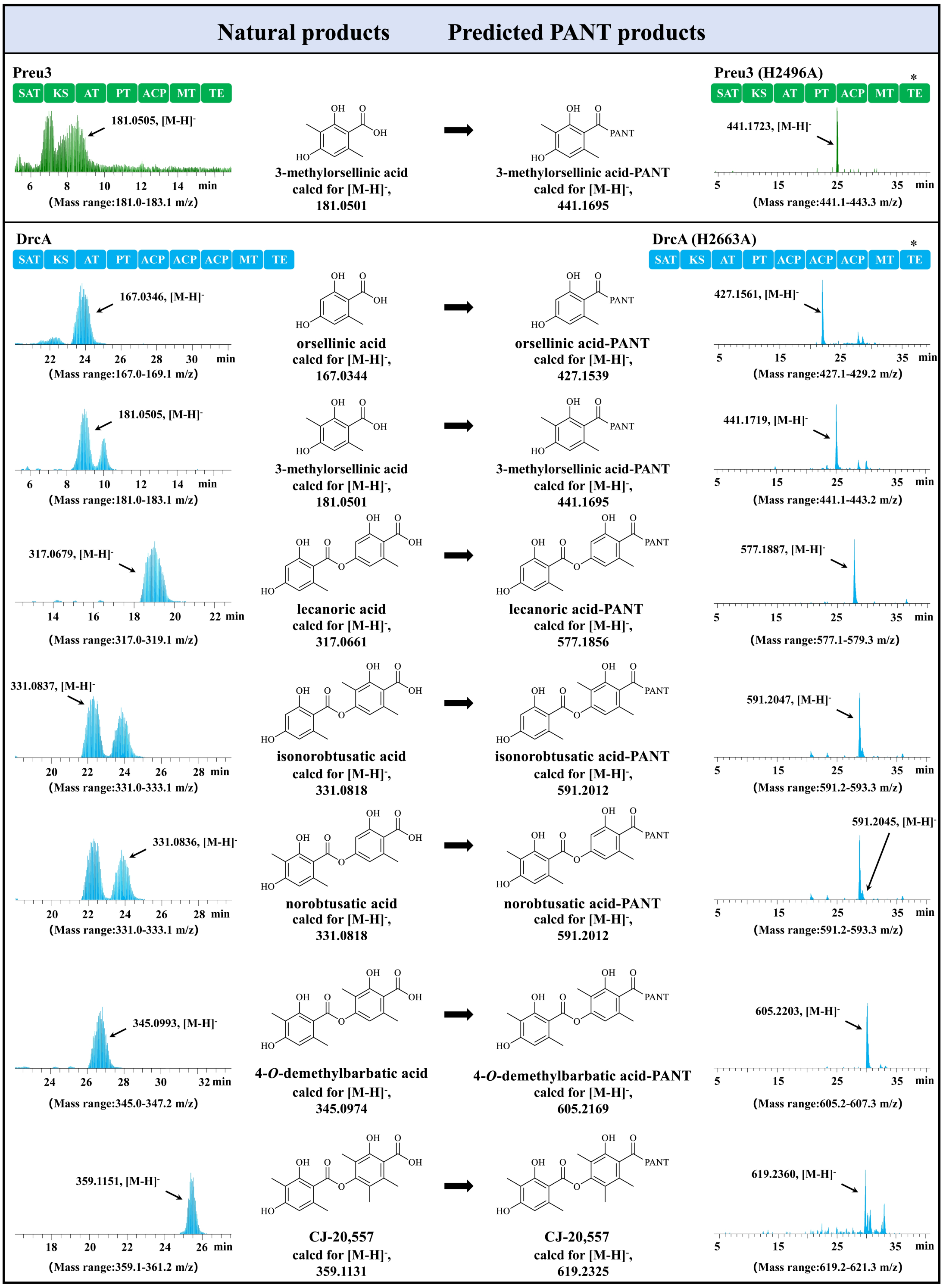

Figure S8. Searching for the corresponding PANT products in the TE-mutated Preu3 and DrcA transformants.

The *S. cerevisiae* BJ5464-NpgA transformants contain the TE-mutated Preu3 and DrcA originally synthesized a spectrum of OA monomers and dimers with different methylation levels.

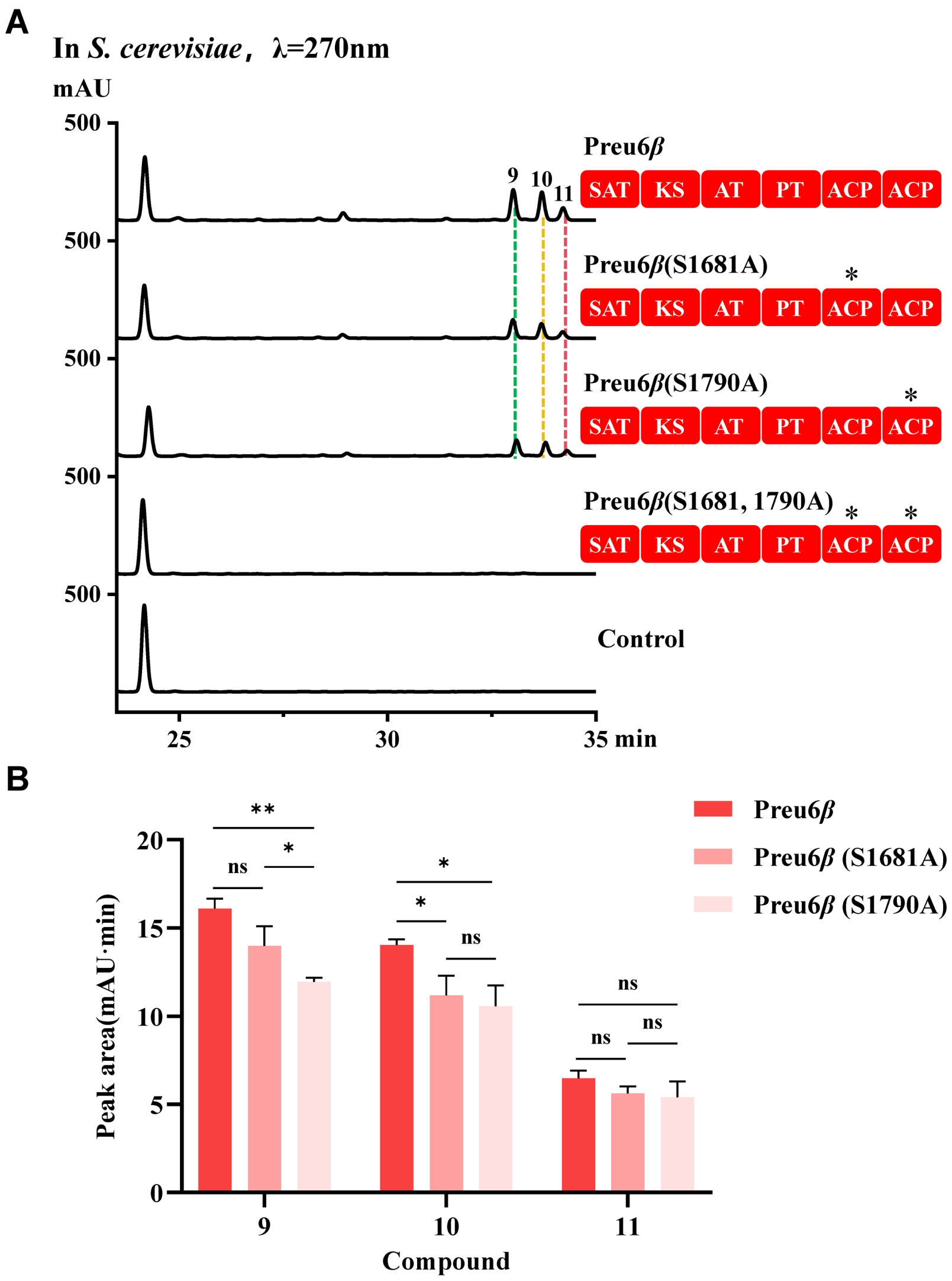

Figure S9. Functional analysis of the tandem ACP domains of Preu6*β* in the production of compounds **9-11**.

A. HPLC analysis (λ = 270 nm) of metabolites produced in *S. cerevisiae* expressing Preu6*β*, the single ACP mutants Preu6*β*(S1681A) and Preu6*β*(S1790A), the double ACP mutant Preu6*β*(S1681A, S1790A). * indicates the inactivated ACP domains.

B. Quantitative analysis of peak areas corresponding to compounds **9**, **10**, and **11**. * indicates $P < 0.05$, ** indicates $P < 0.01$, and ns indicates not significant.

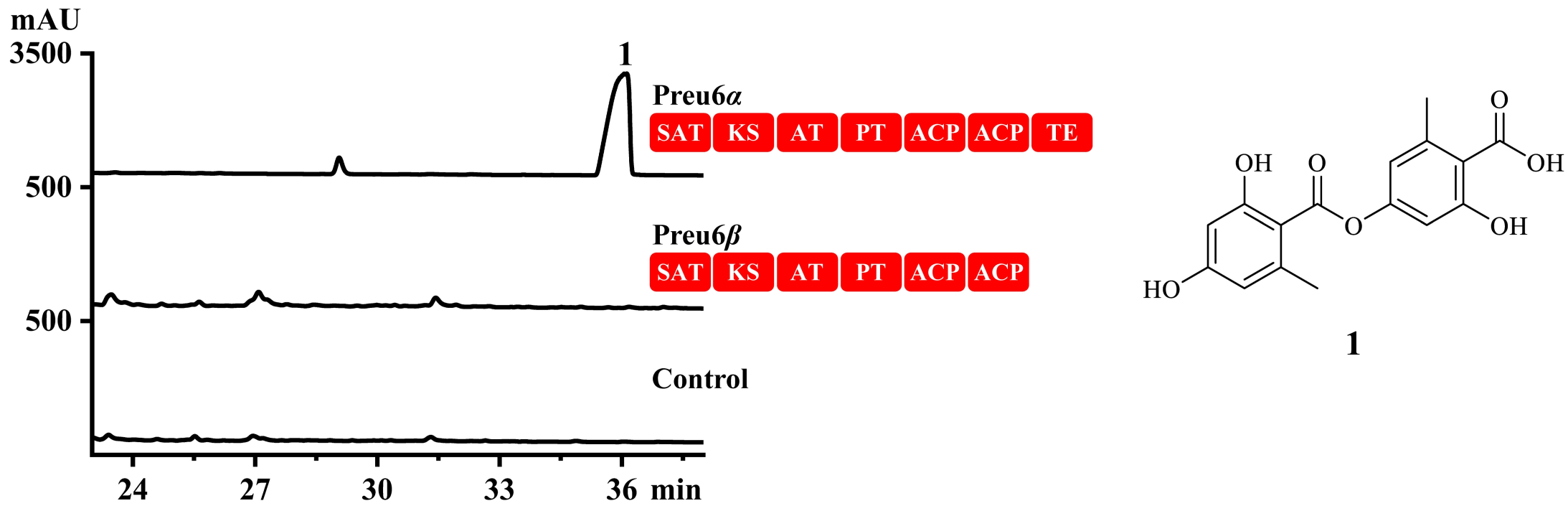

1

Figure S10. Metabolite analysis of Preu6*β* expressed in *A. oryzae* (λ = 300 nm). Control, the corresponding empty vector.

A

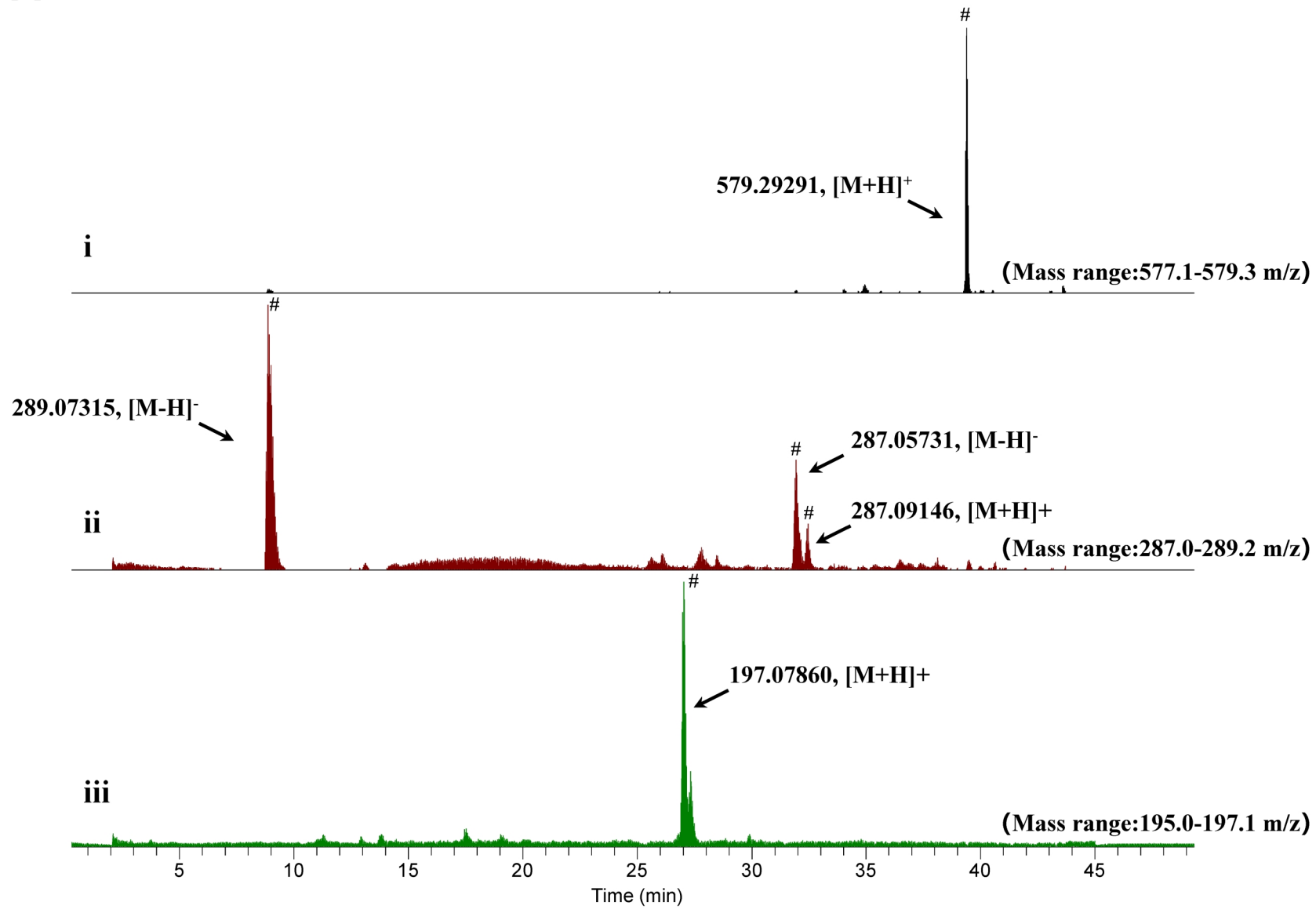

B

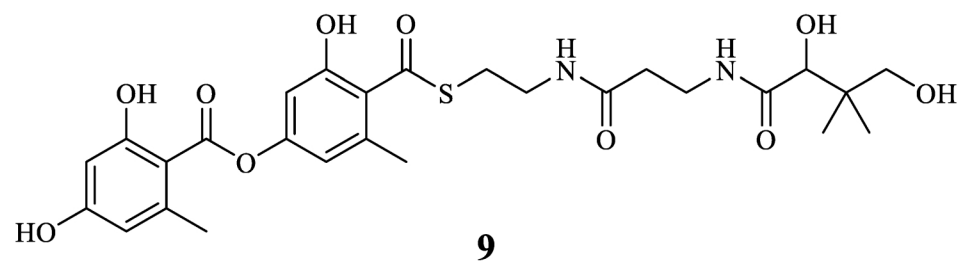

9

calcd for [M+H]+, 579.2012
[M-H]-, 577.1856

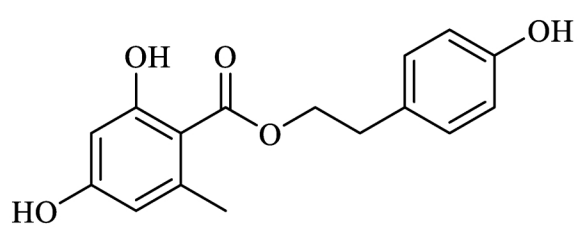

10

calcd for [M+H]+, 289.1076
[M-H]-, 287.0919

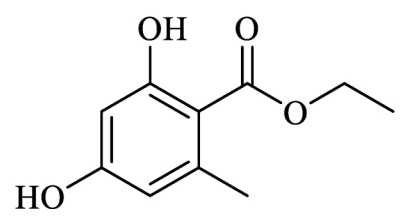

11

calcd for [M+H]+, 197.0814
[M-H]-, 195.0657

C

Retention time (min)	Calculated molecular weight	Measured molecular weight	Mass error value (ppm)
39.42	$[M+H]^+$ 579.2012	$[M+H]^+$ 579.29291	158.34
8.86	$[M-H]^-$ 287.0919	$[M-H]^-$ 289.07315	6901.10
31.95	$[M-H]^-$ 287.0919	$[M-H]^-$ 287.05731	120.48
32.39	$[M+H]^+$ 289.1076	$[M+H]^+$ 287.09146	6973.67
27.07	$[M+H]^+$ 197.0814	$[M+H]^+$ 197.07860	14.21

Figure S11. HRESIMS analysis of compounds **9-11** in the native strain XL-1326.

A. EICs of XL-1326 crude extracts at *m/z* 577.1-579.3 for **9**, *m/z* 287.0-289.2 for **10**, and *m/z* 195.0-197.1 for **11**.

B. Structures and calculated molecular weights of compounds **9-11**.

C. Measured ions and corresponding mass errors of the extra peaks, whose mass errors are greater than 10 ppm, excluding the assignment as compounds **9-11**.

Figure S12. UV-VIS spectra of compounds **9-11**.

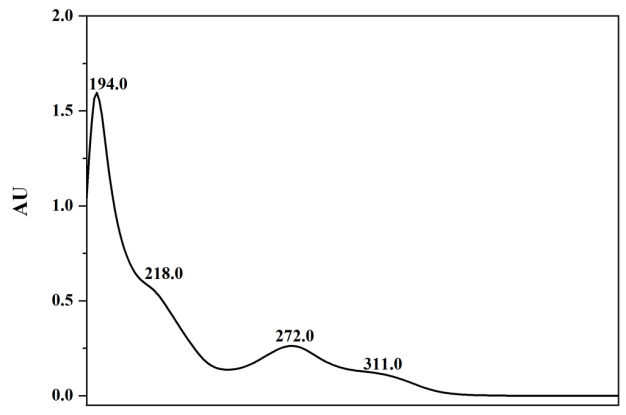

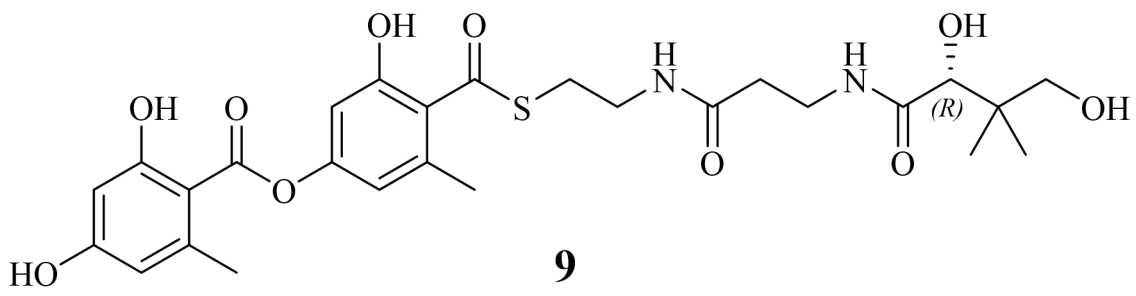

9

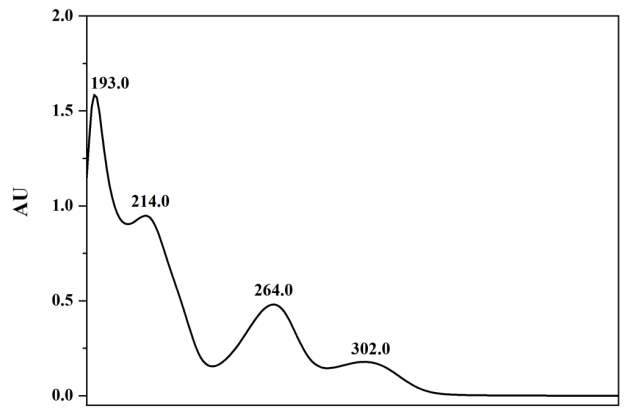

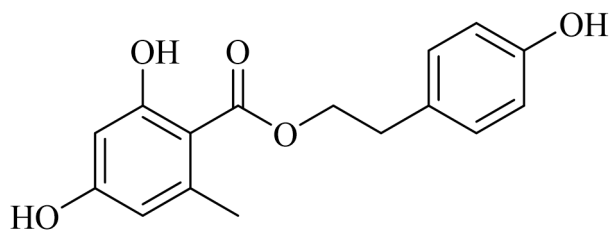

10

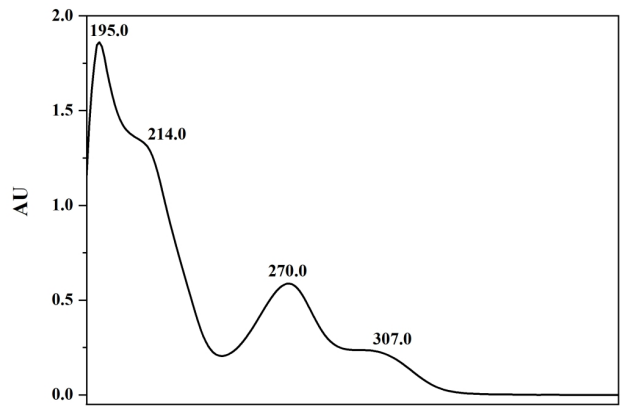

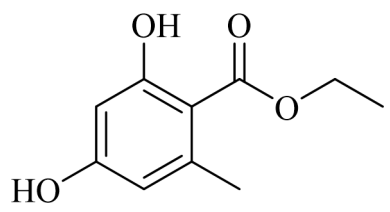

11

Figure S13. HRESIMS spectra of the related compounds in this study.

Figure S13.1. (+)-HRESIMS spectrum of **9**.

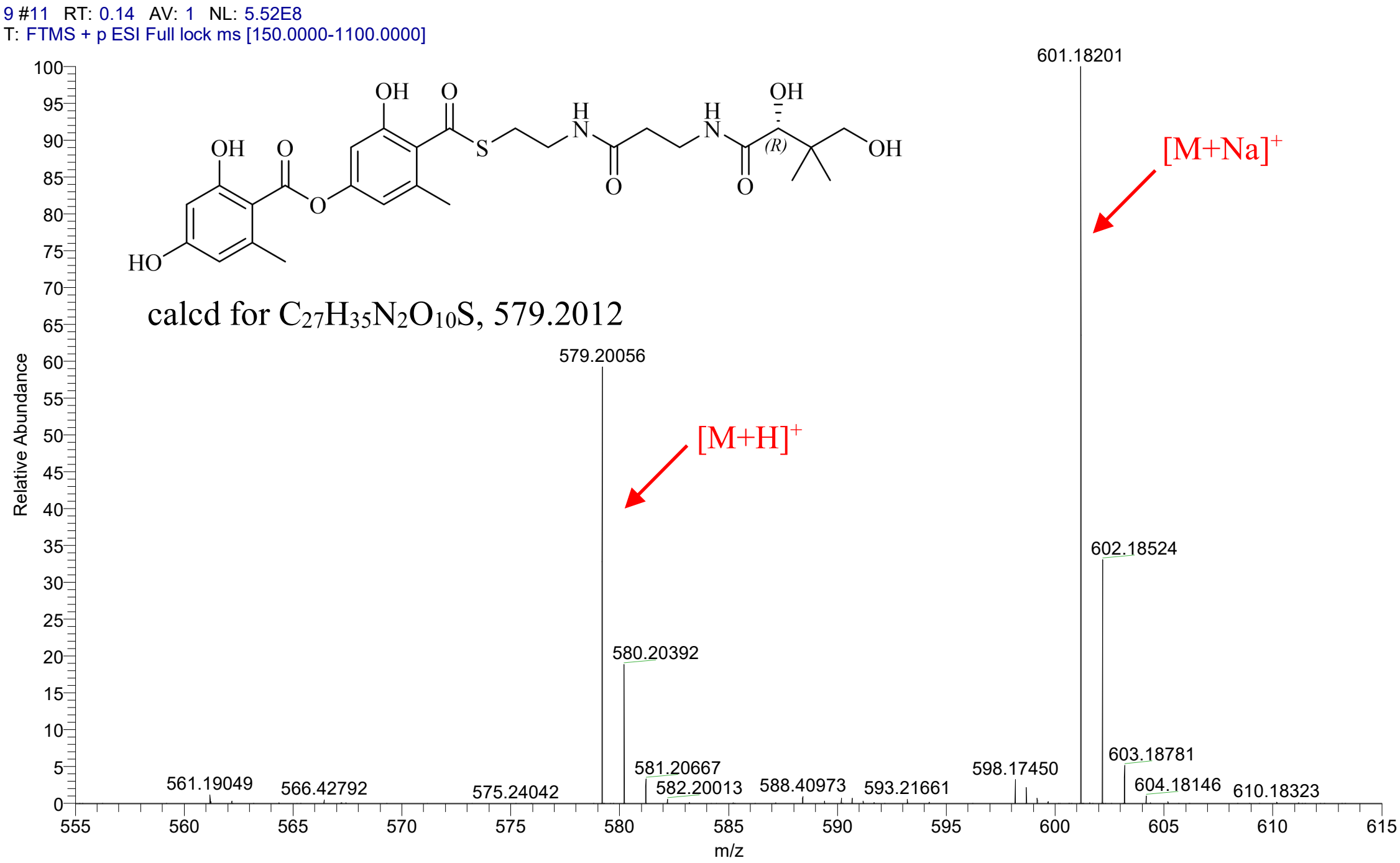

Figure S13.2. (+)-HRESIMS spectrum of **10**.

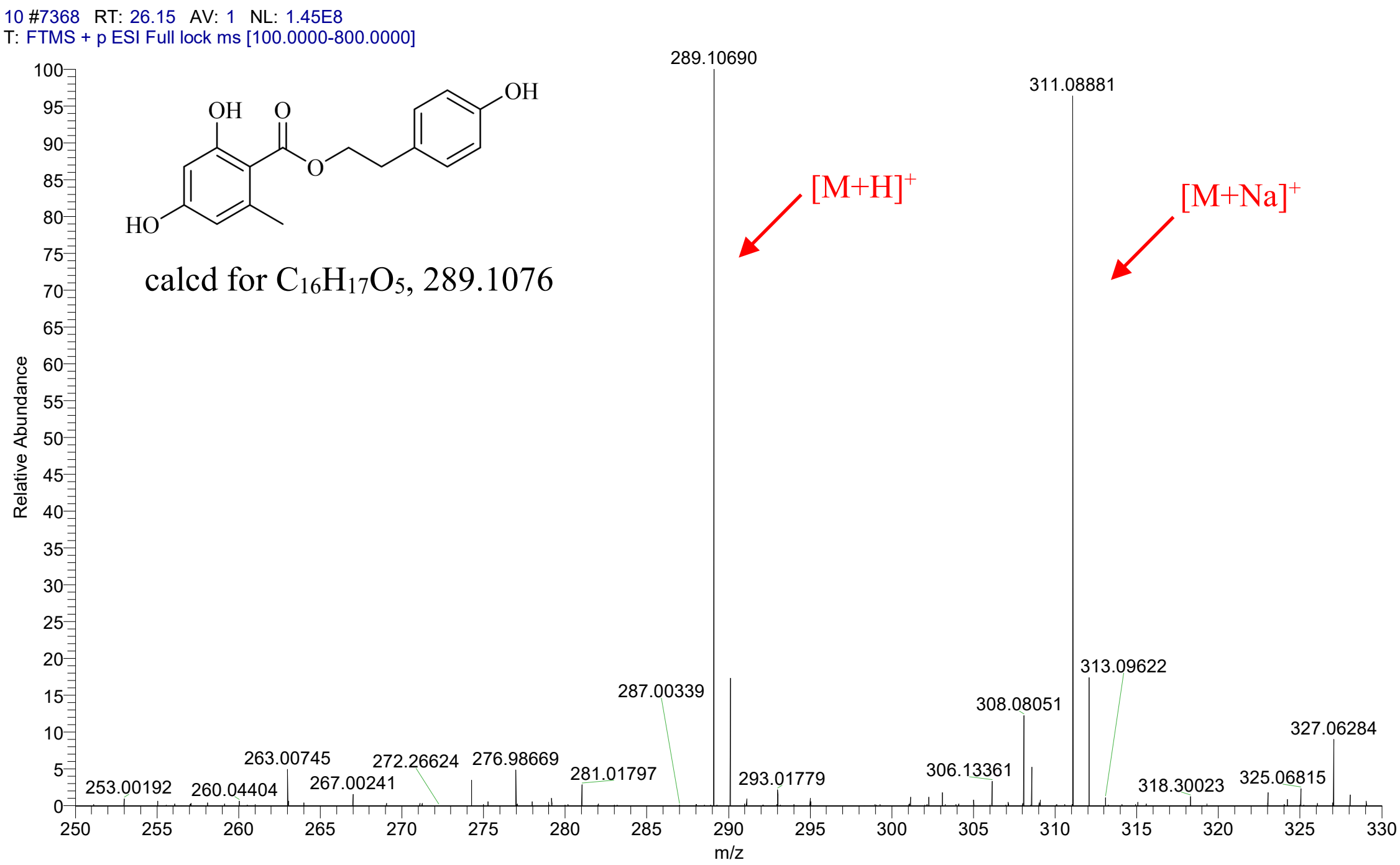

Figure S13.3. (+)-HRESIMS spectrum of **11**.

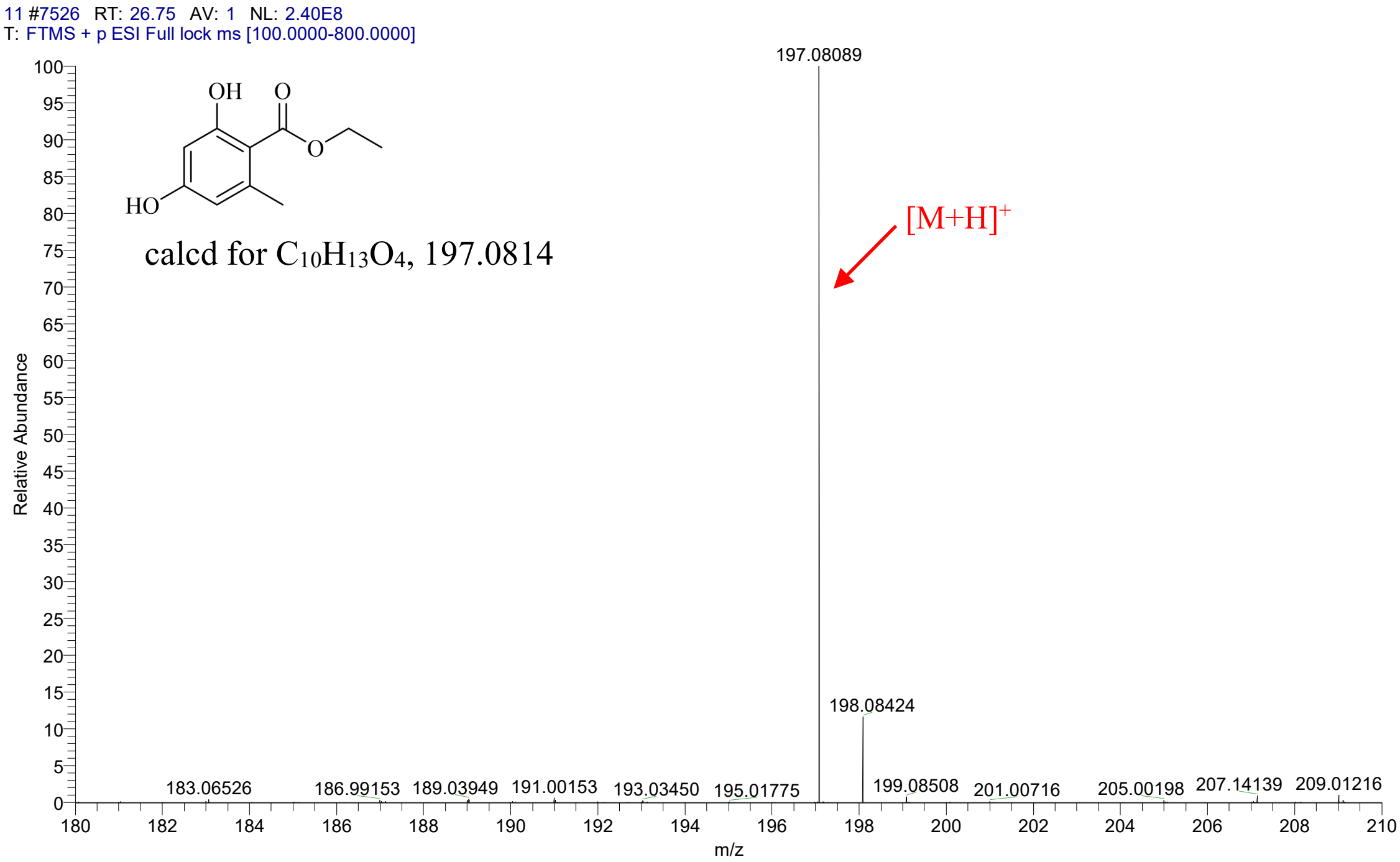

Figure S13.4. (+)-HRESIMS spectrum of **12**.

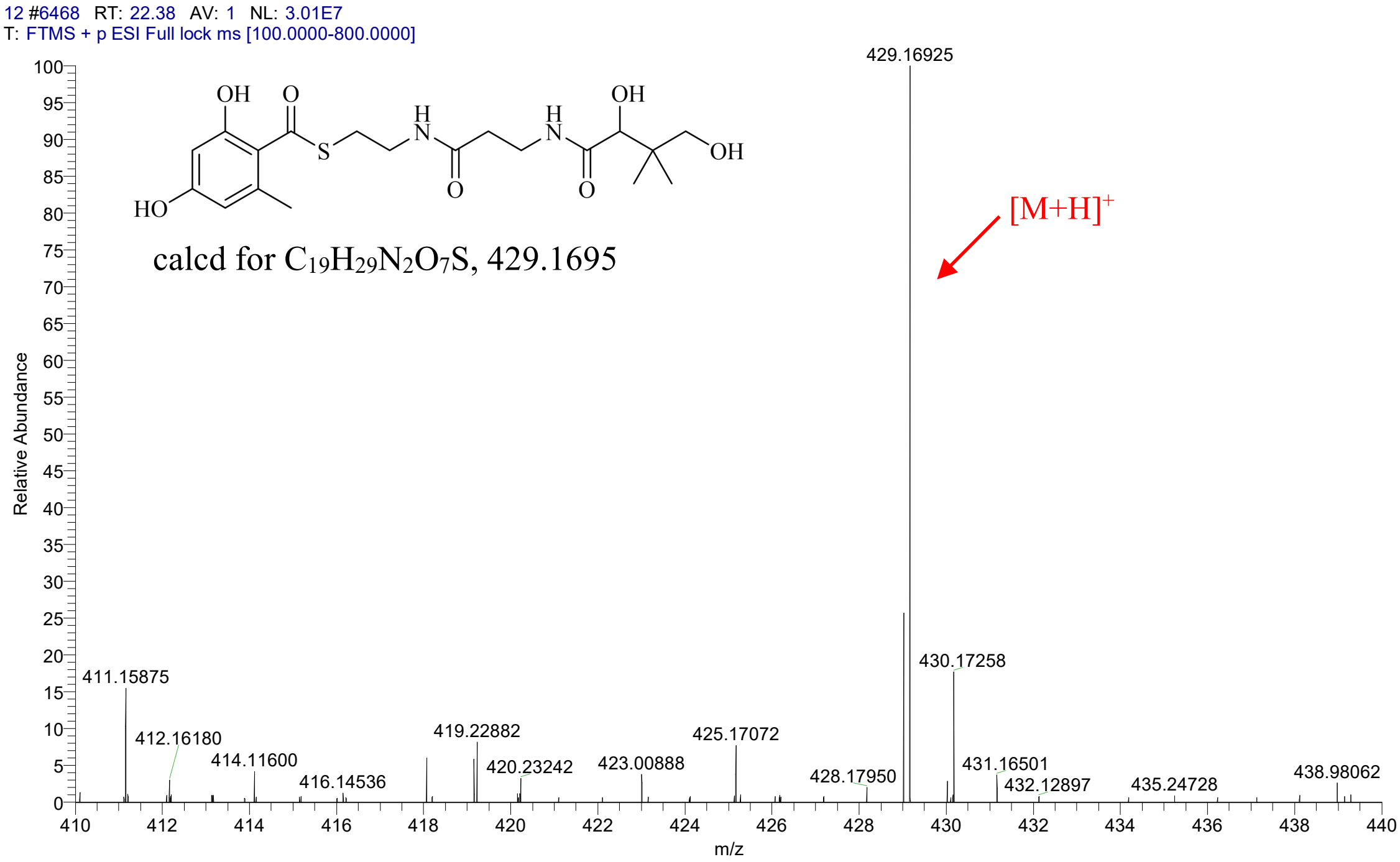

Figure S13.5. (-)-HRESIMS spectrum of orsellinic acid.

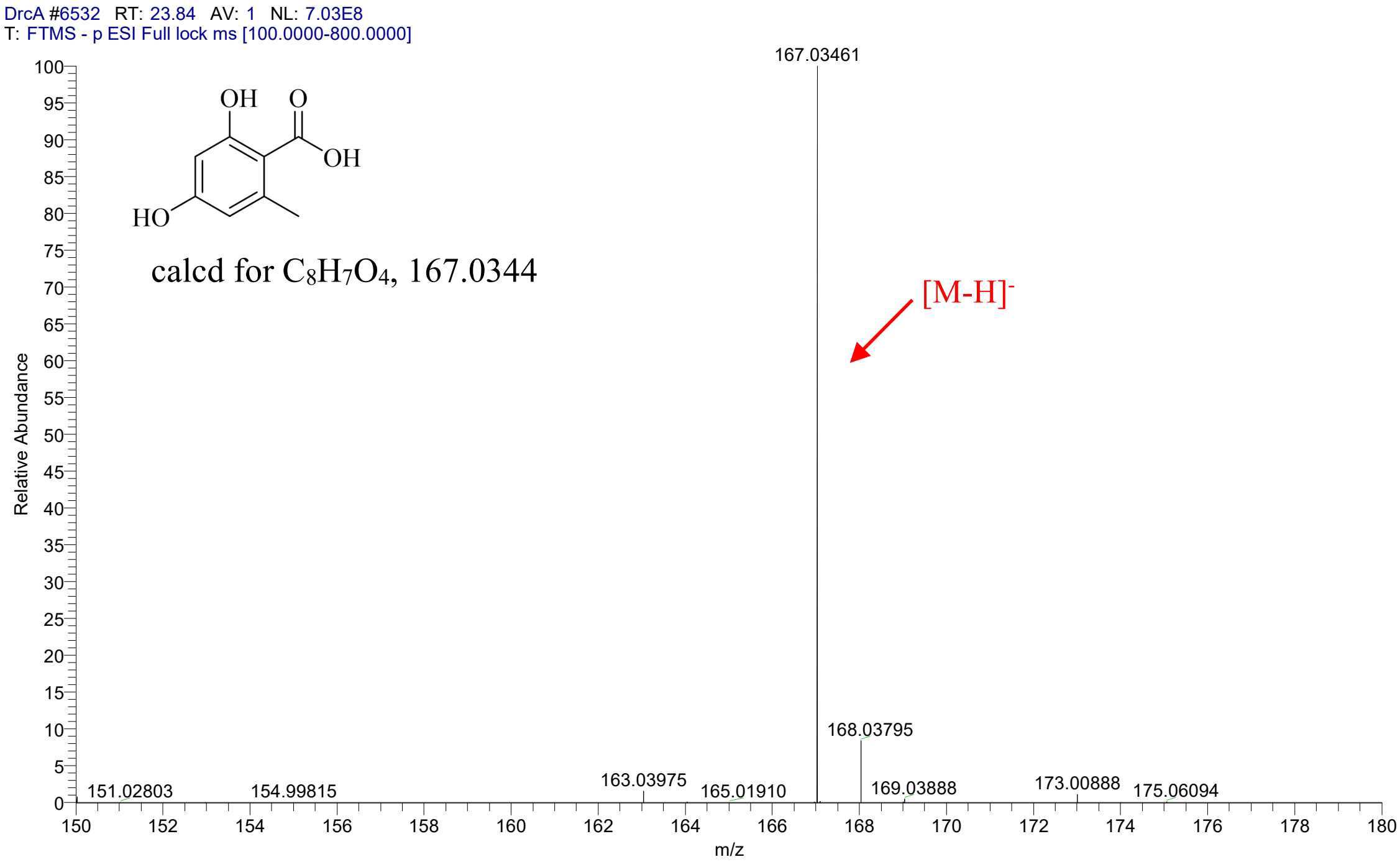

Figure S13.6. (-)-HRESIMS spectrum of lecanoric acid.

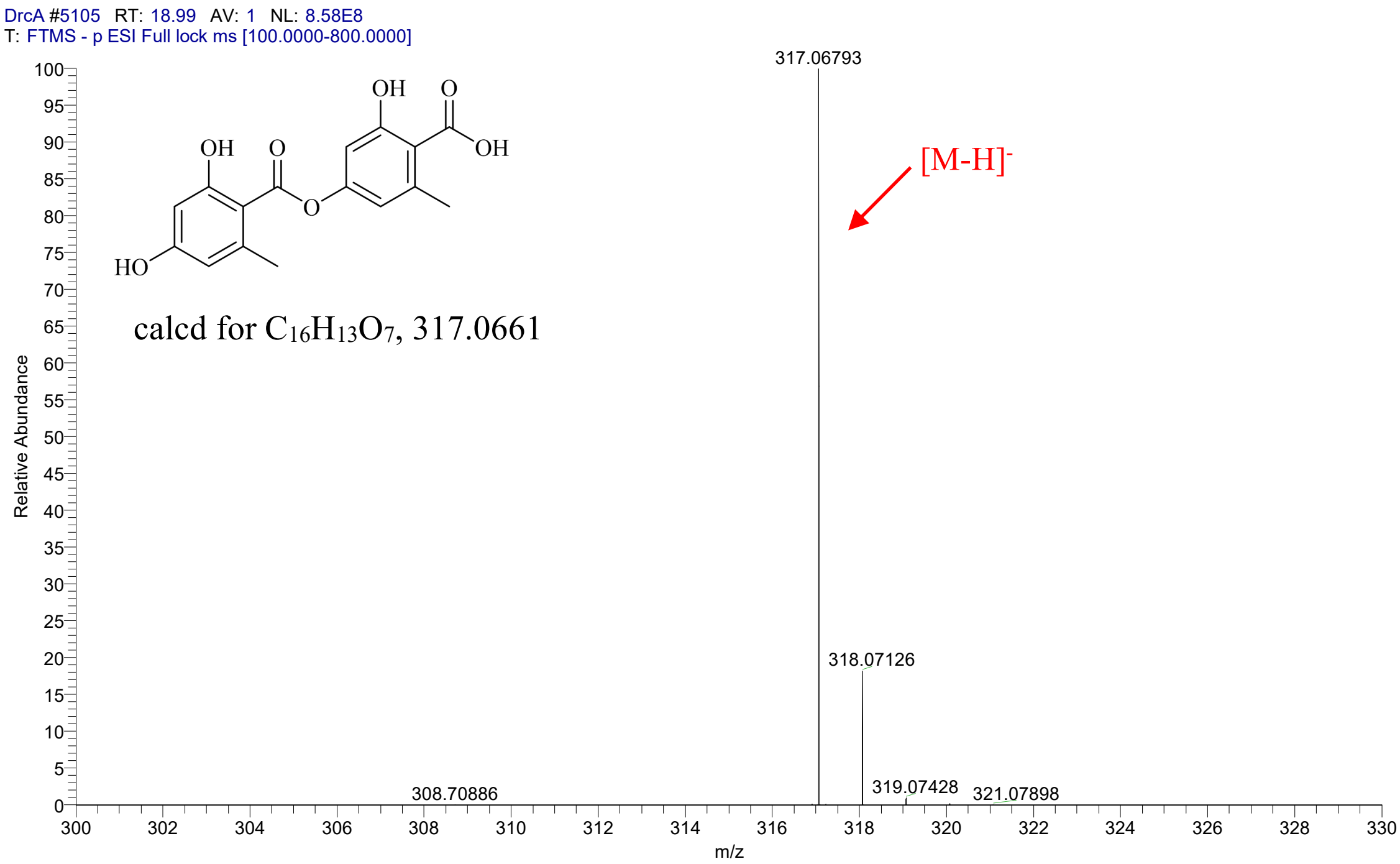

Figure S13.7. (+)-HRESIMS spectrum of prephenalenone.

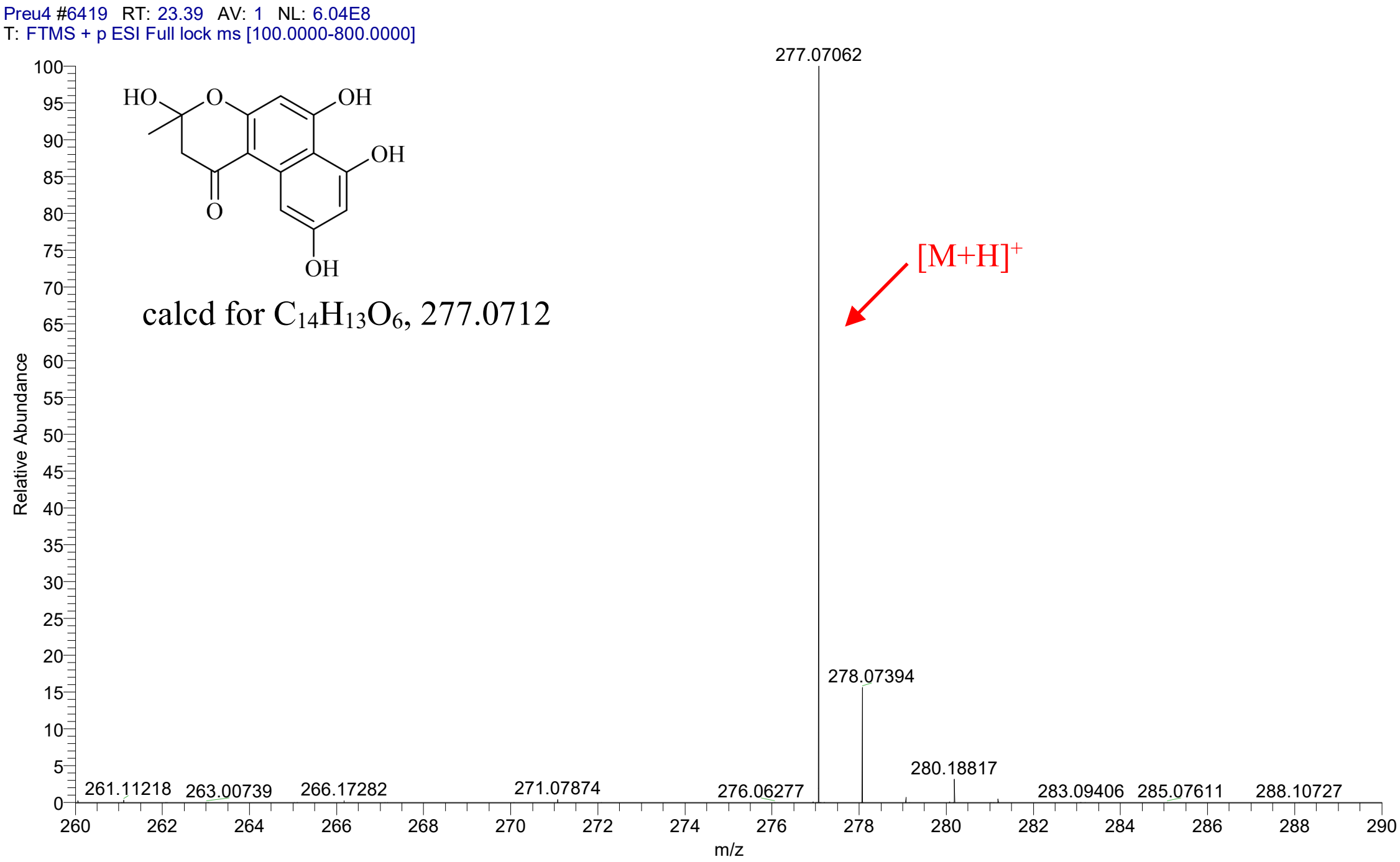

Figure S13.8. (+)-HRESIMS spectrum of atrochrysone.

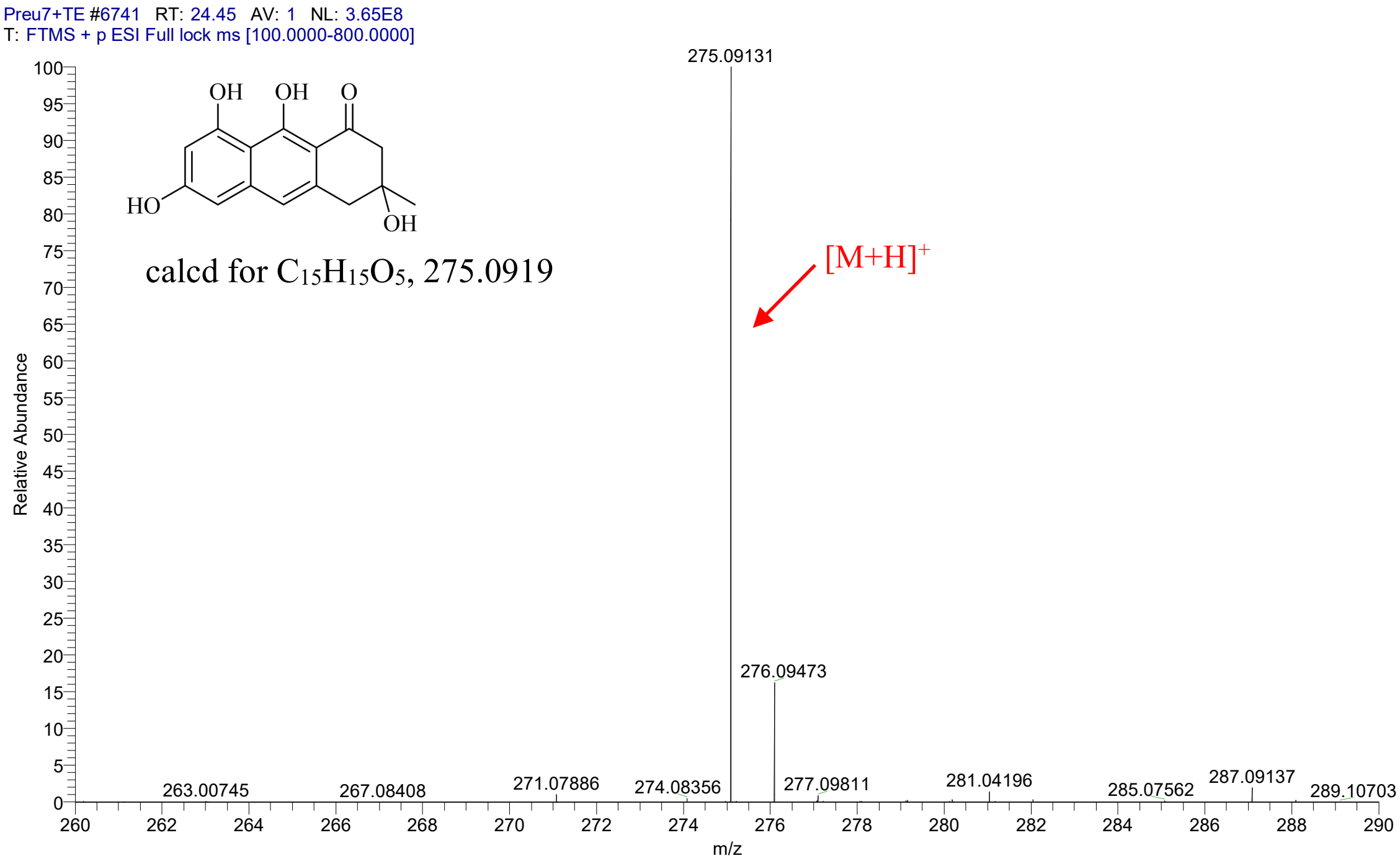

Figure S13.9. (+)-HRESIMS spectrum of desmethyl-lasiodiplodin.

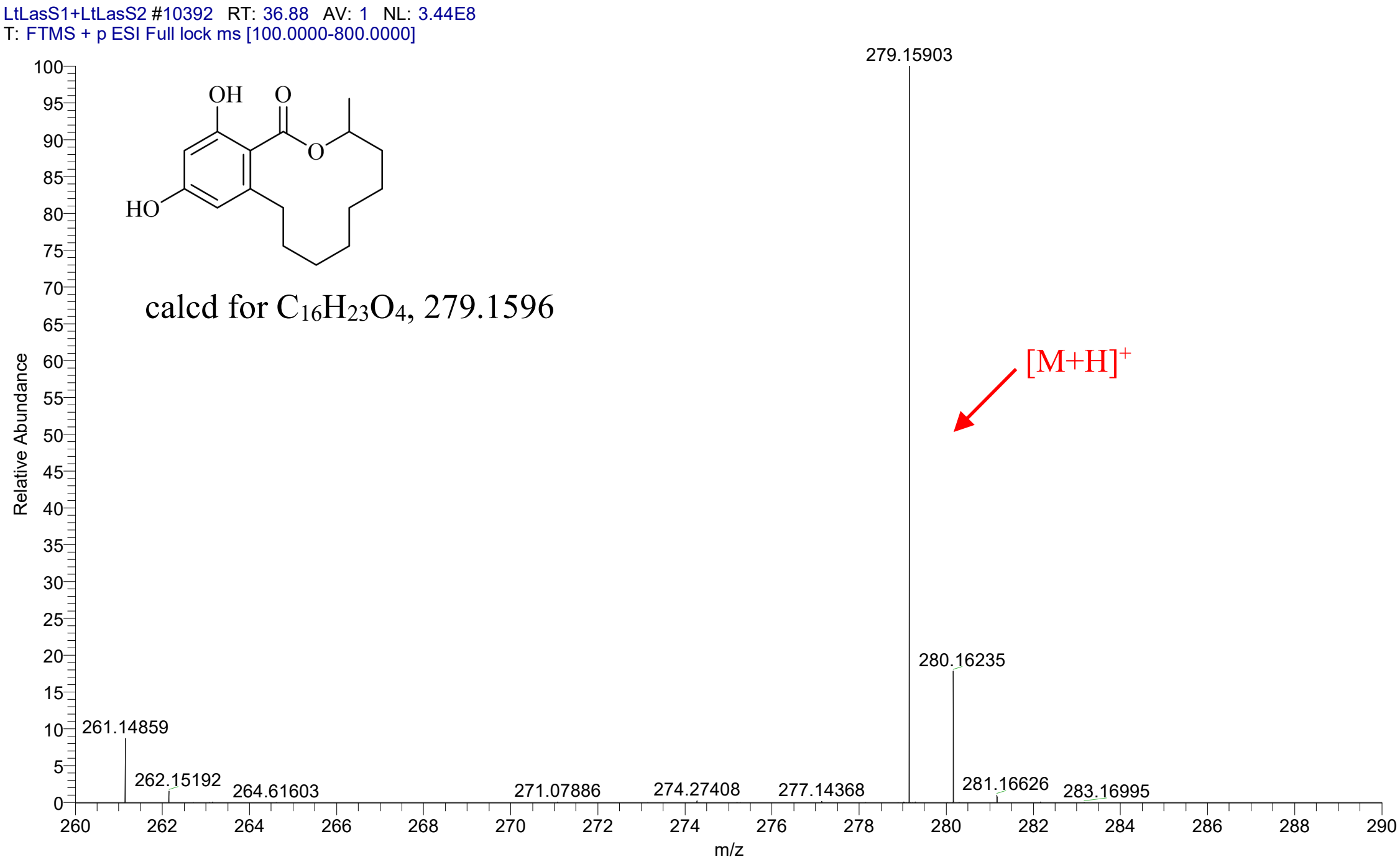

Figure S13.10. (+)-HRESIMS spectrum of 10,11-dehydrocurvularin.

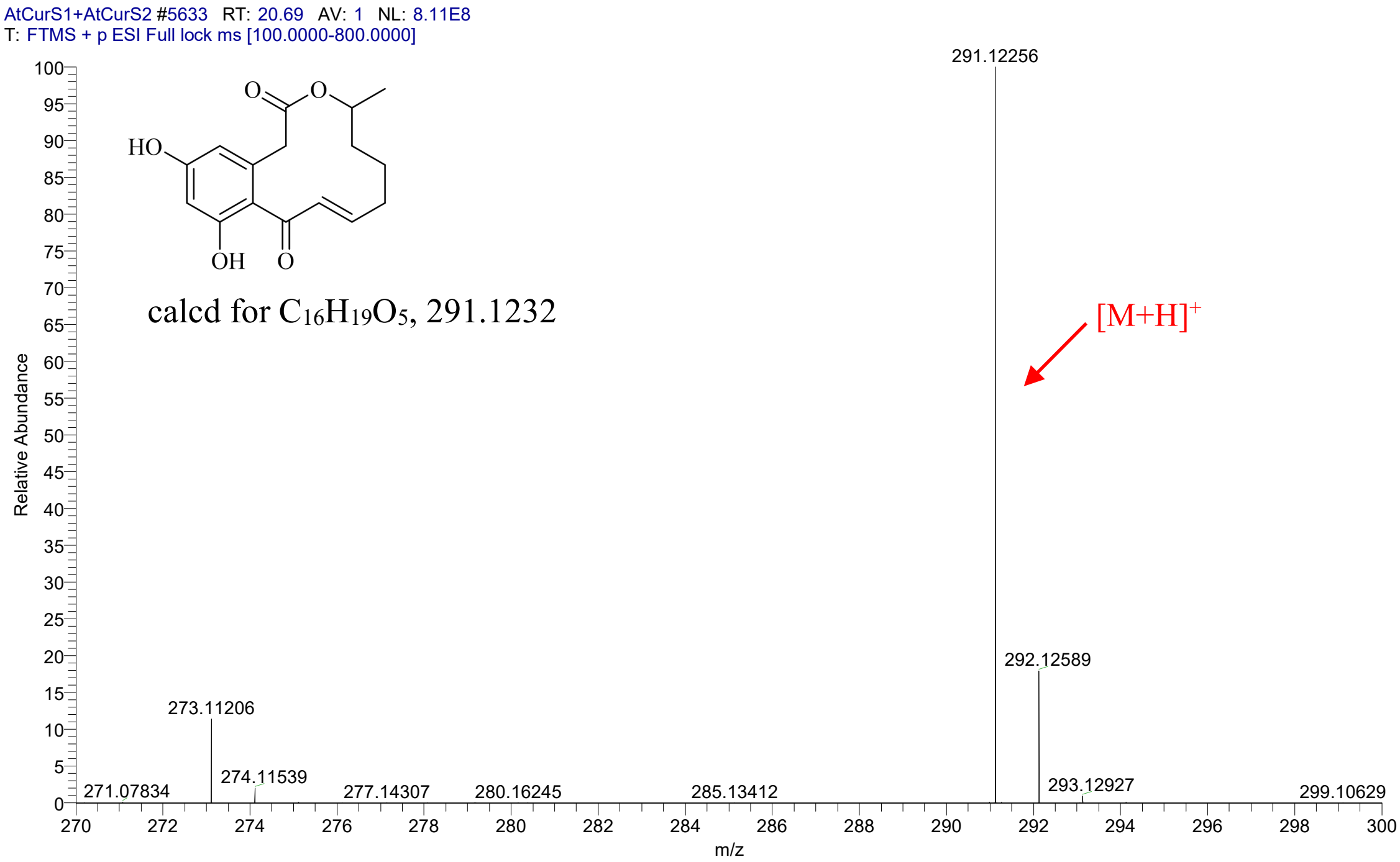

Figure S13.11. (-)-HRESIMS spectrum of 3-methylorsellinic acid.

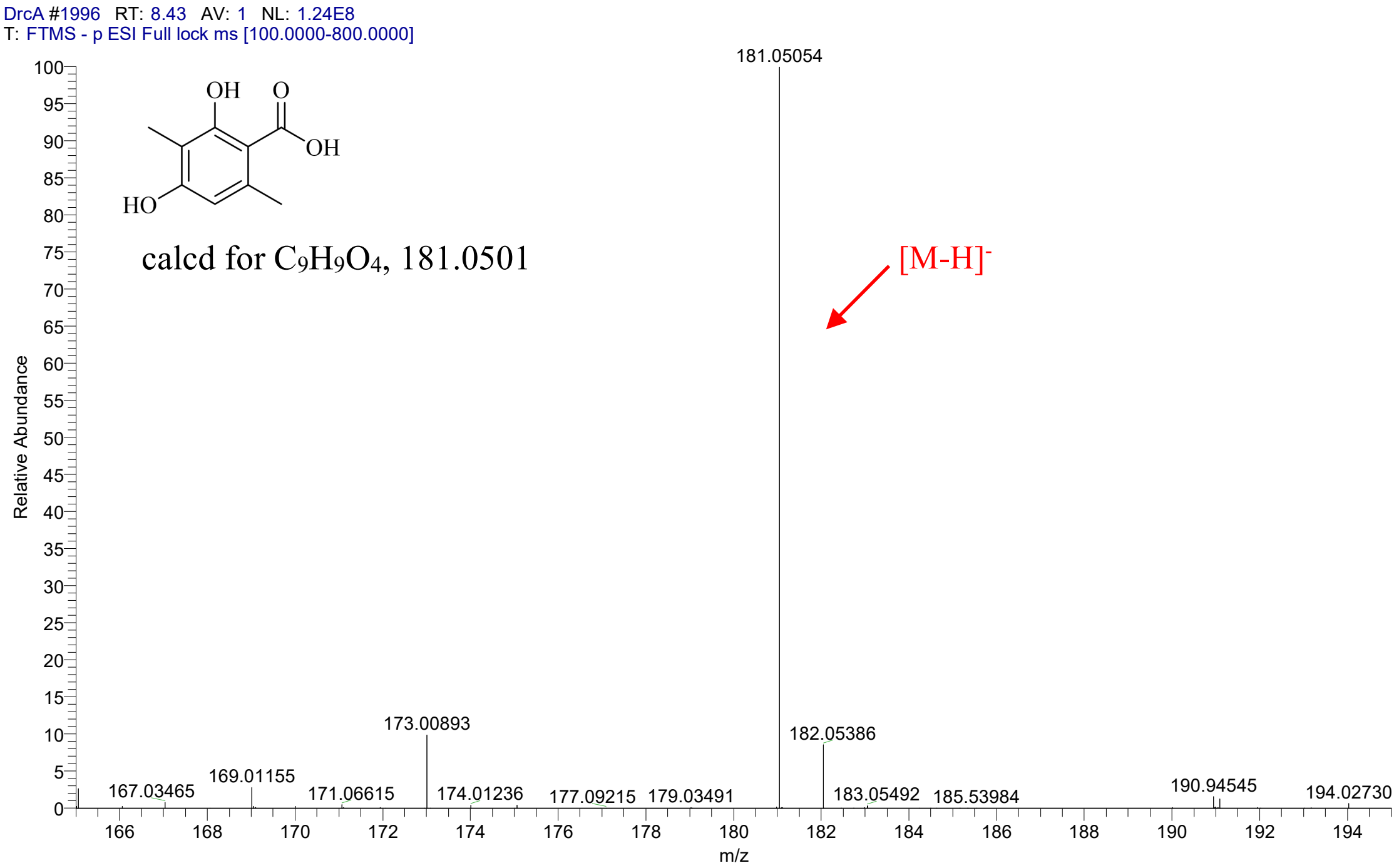

Figure S13.12. (-)-HRESIMS spectrum of 3-methylorsellinic acid-PANT.

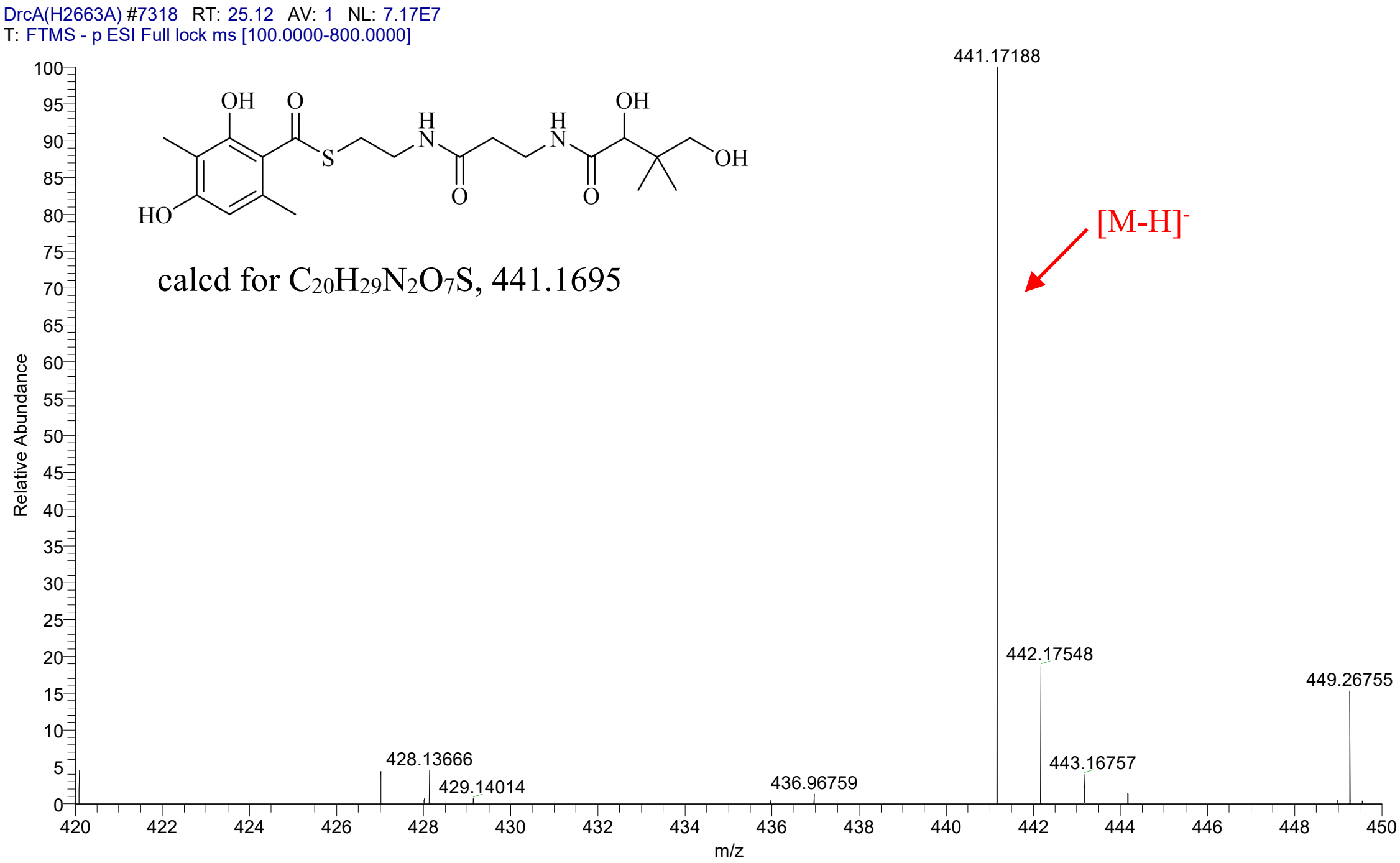

Figure S13.13. (-)-HRESIMS spectrum of isonorobtusatic acid.

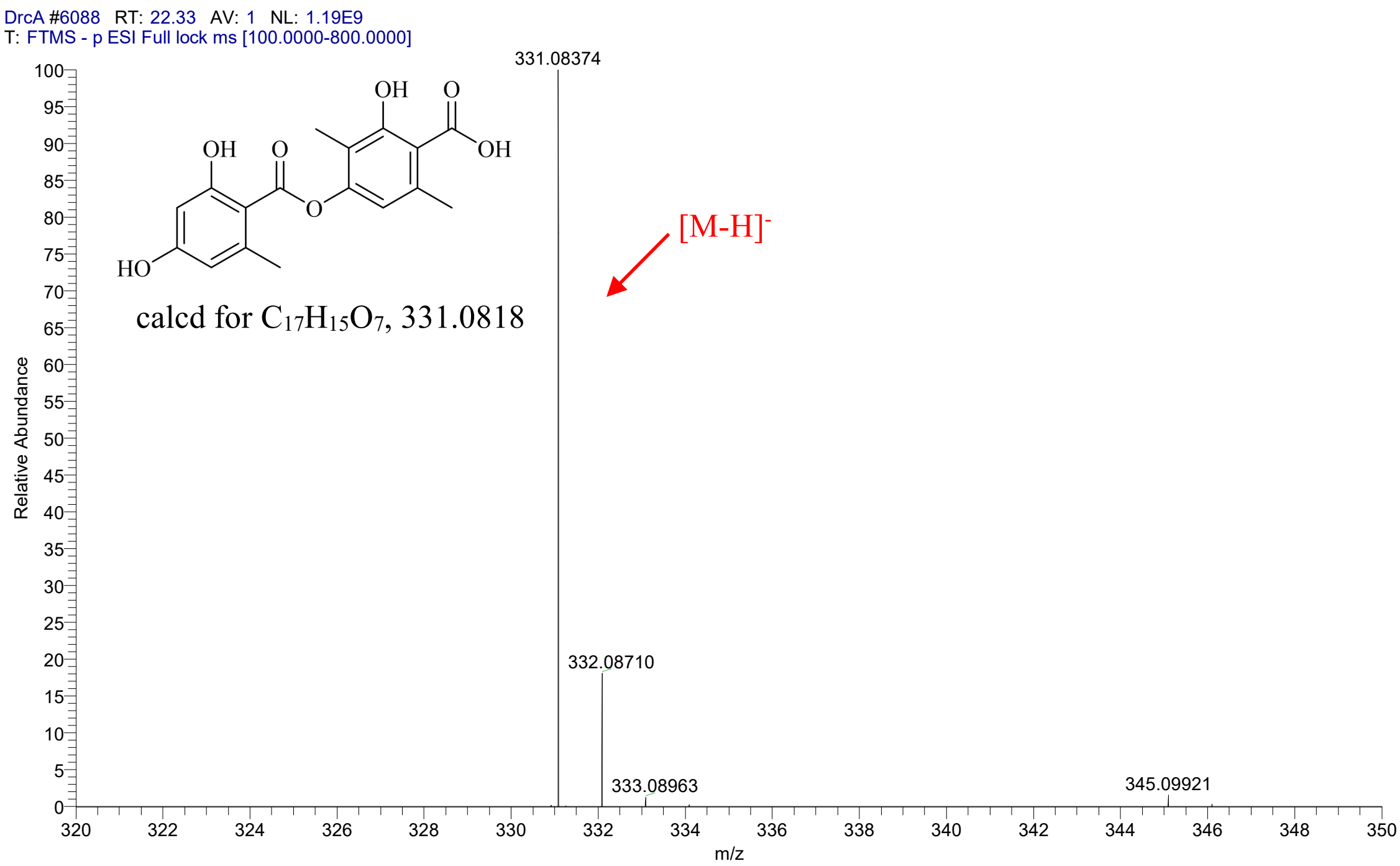

Figure S13.14. (-)-HRESIMS spectrum of isonorobtusatic acid-PANT.

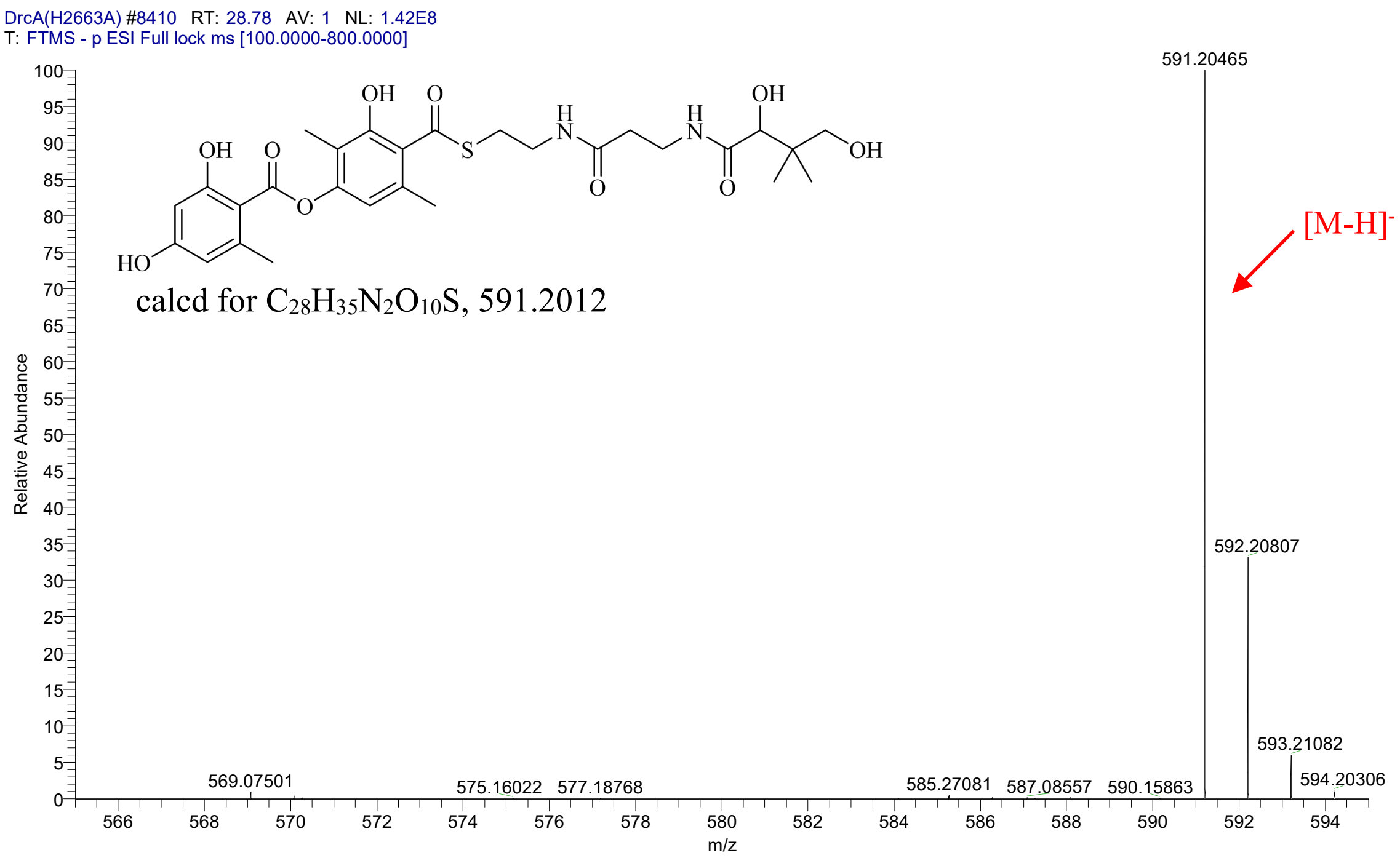

Figure S13.15. (-)-HRESIMS spectrum of norobtusatic acid.

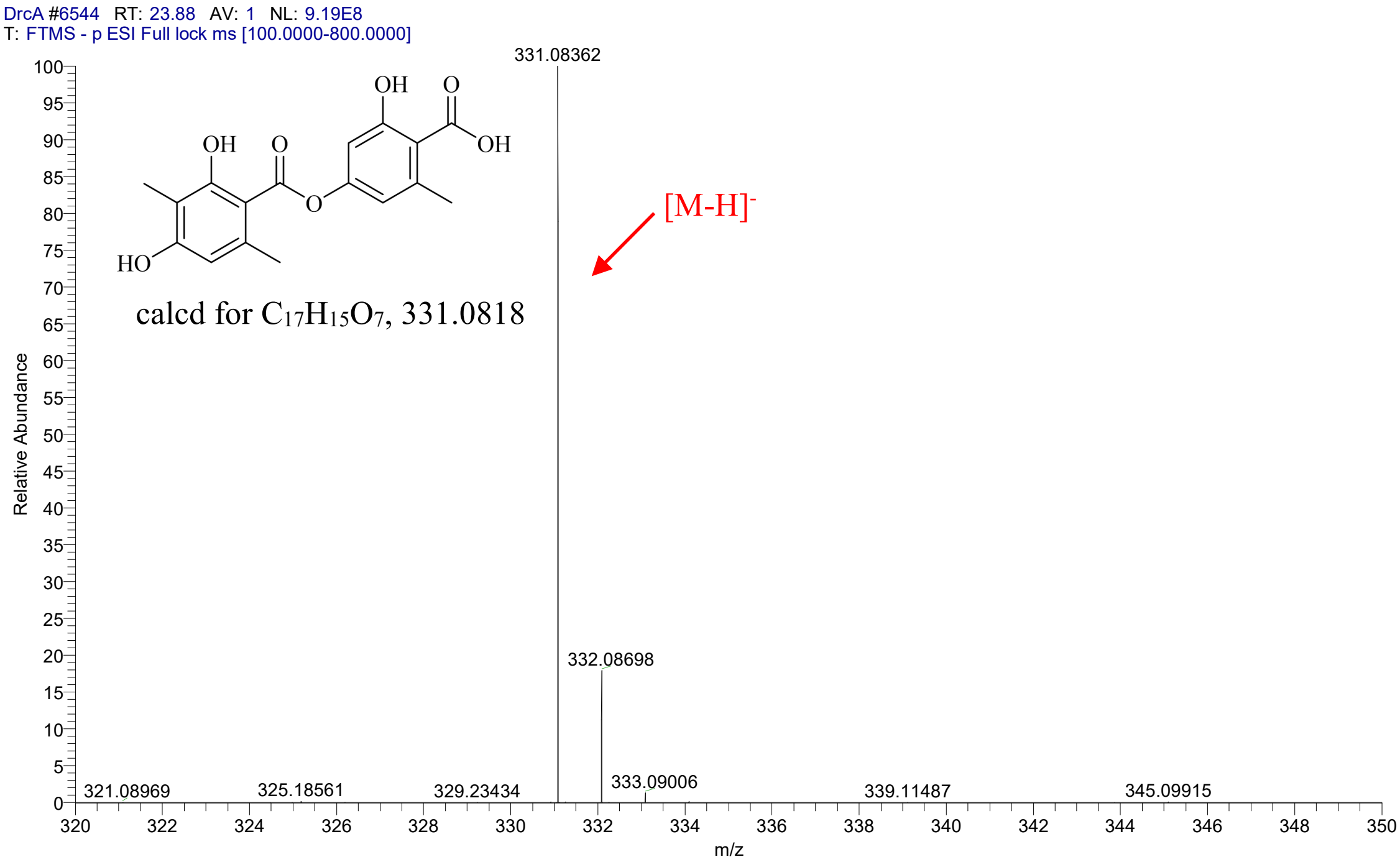

Figure S13.16. (-)-HRESIMS spectrum of norobtusatic acid-PANT.

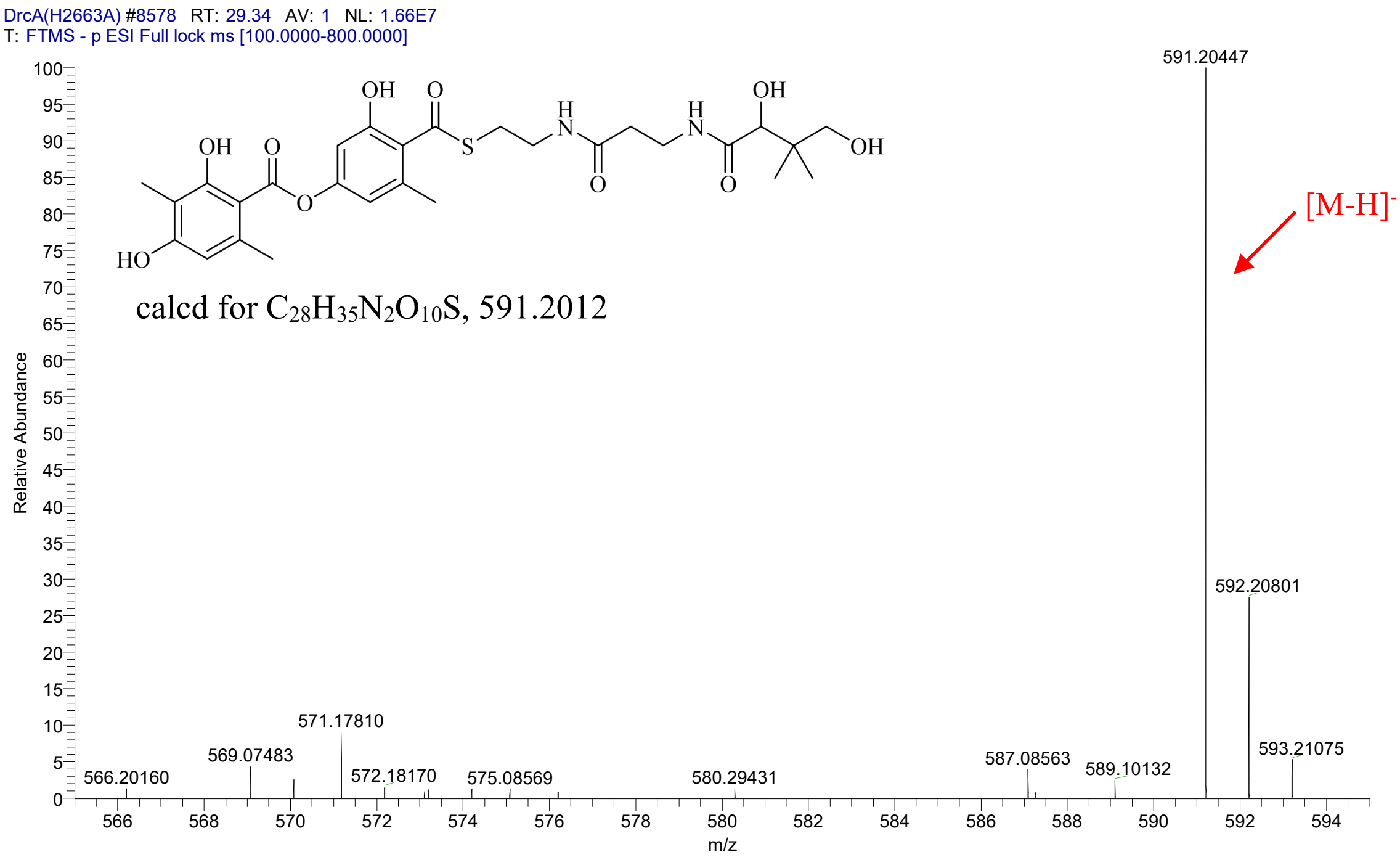

Figure S13.17. (-)-HRESIMS spectrum of 4-*O*-demethylbarbatic acid.

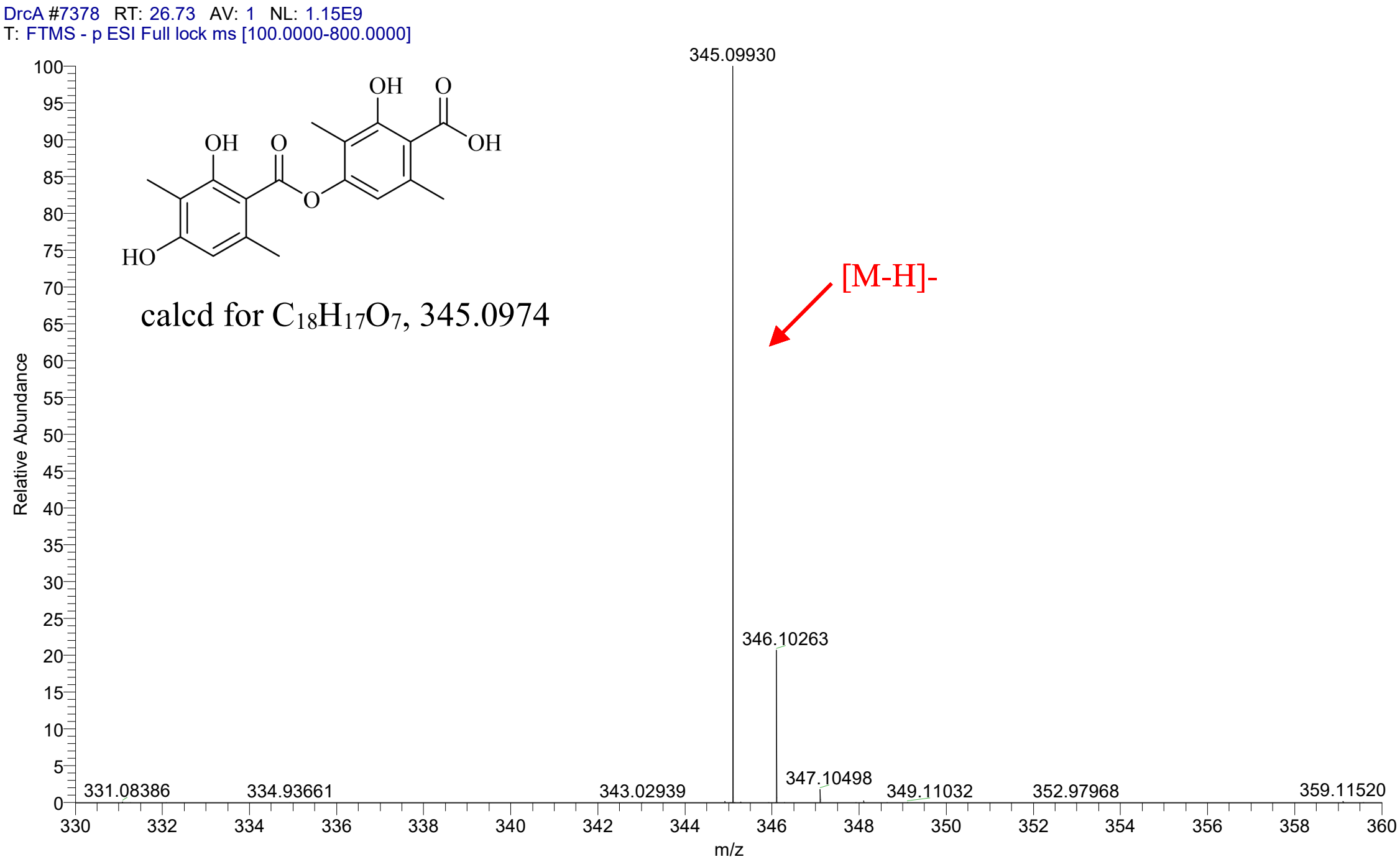

Figure S13.18. (-)-HRESIMS spectrum of 4-*O*-demethylbarbatic acid-PANT.

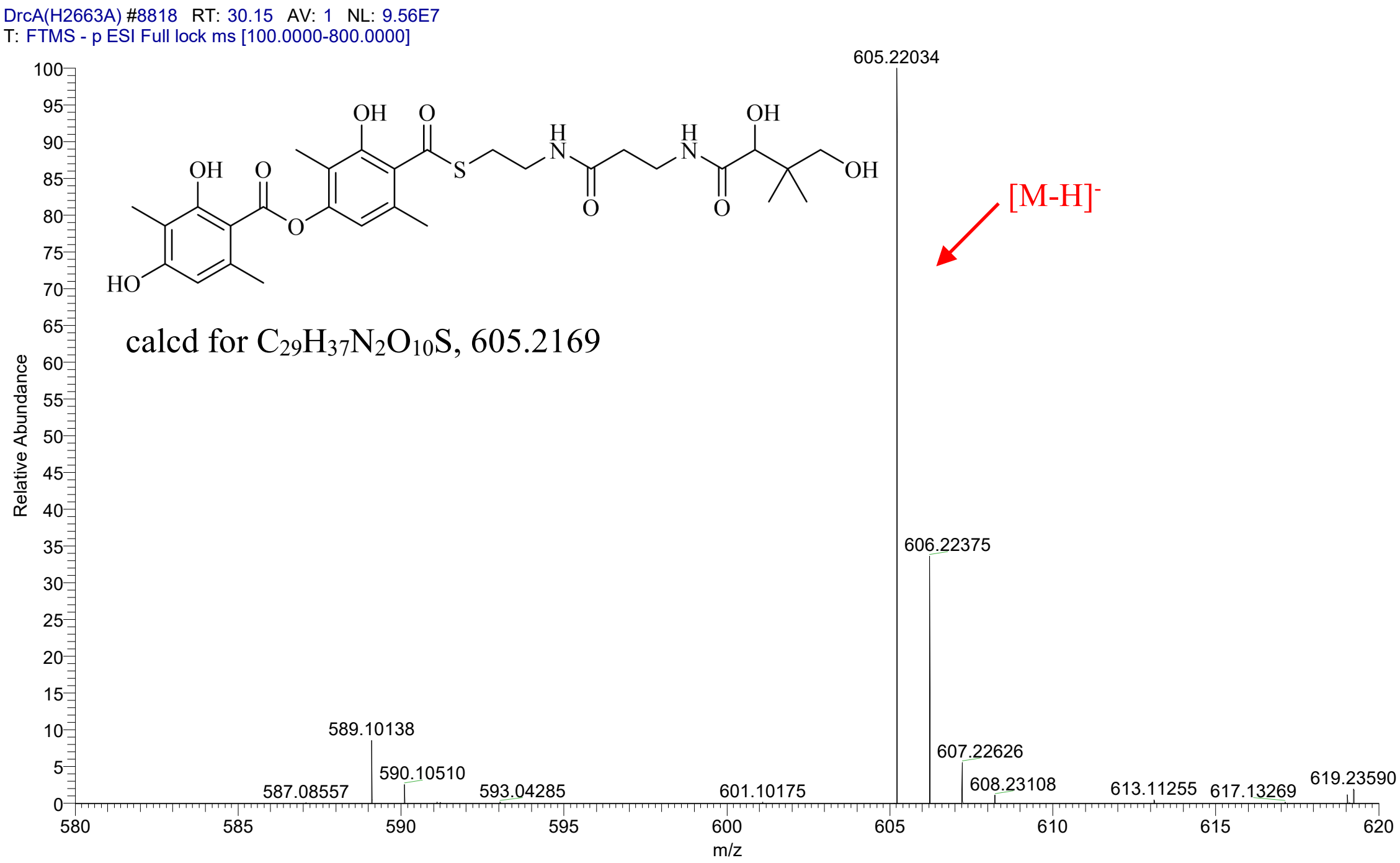

Figure S13.19. (-)-HRESIMS spectrum of CJ-20,557.

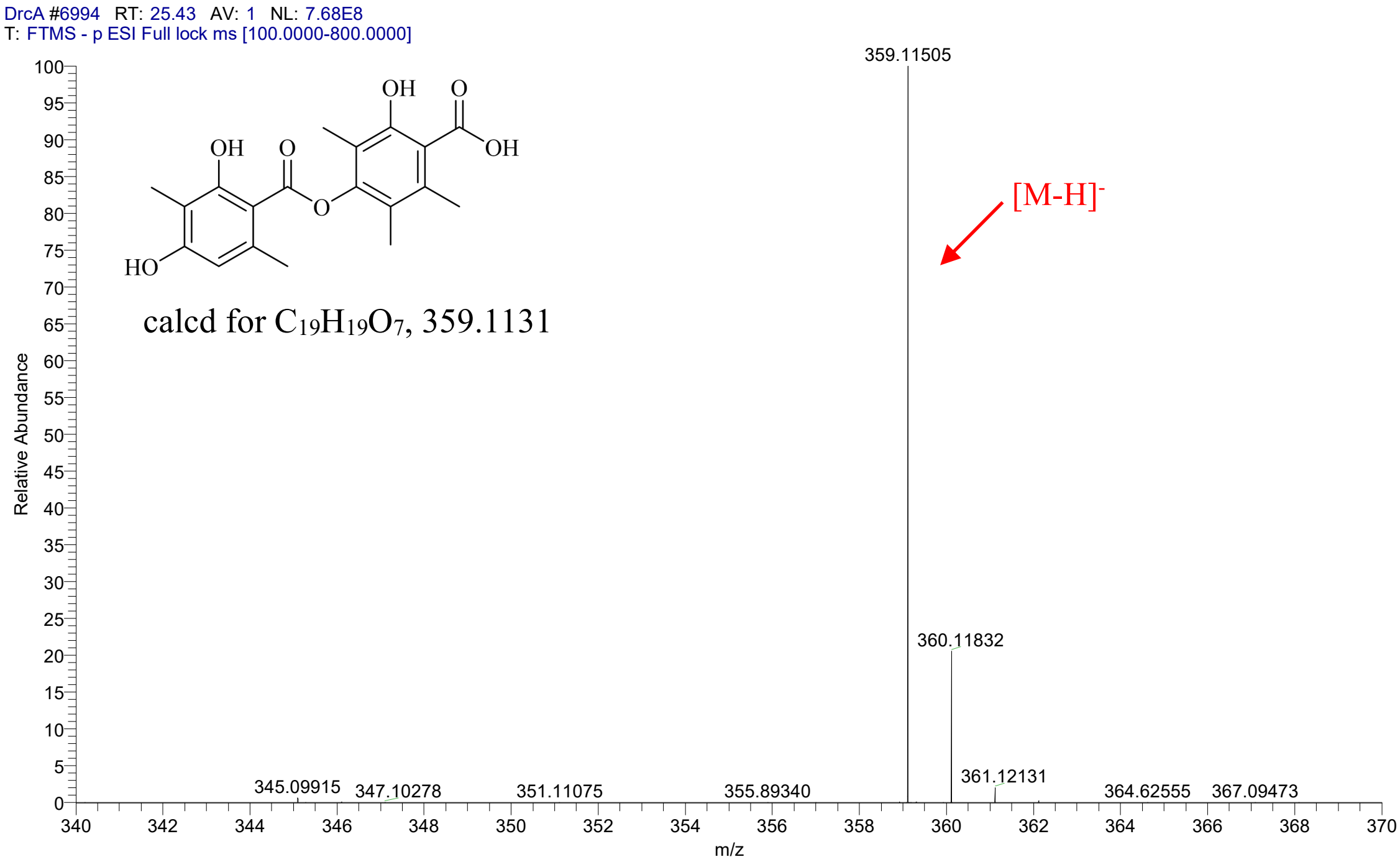

Figure S13.20. (-)-HRESIMS spectrum of CJ-20,557-PANT.

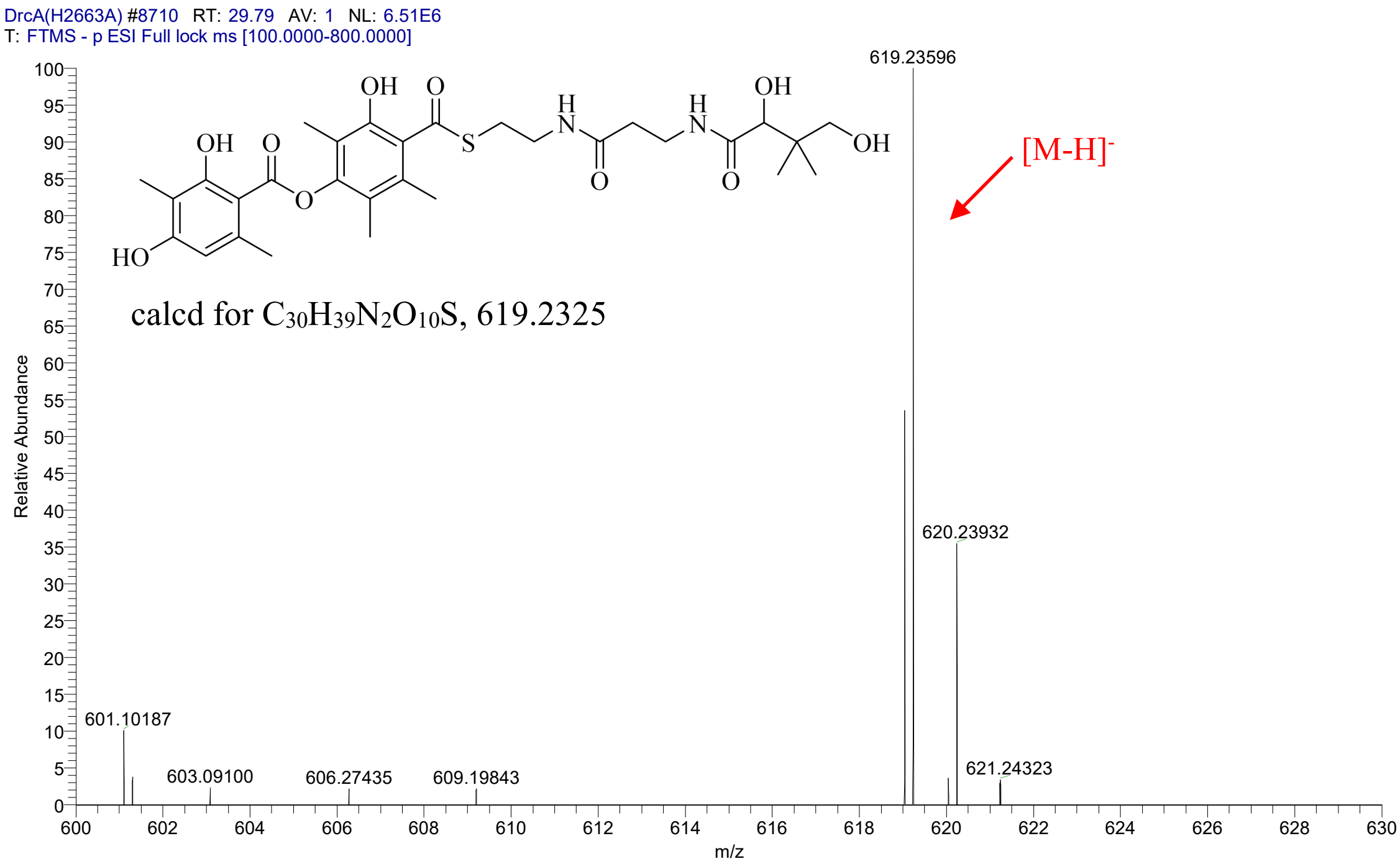

Figure S14. NMR spectra of the OA derivatives.

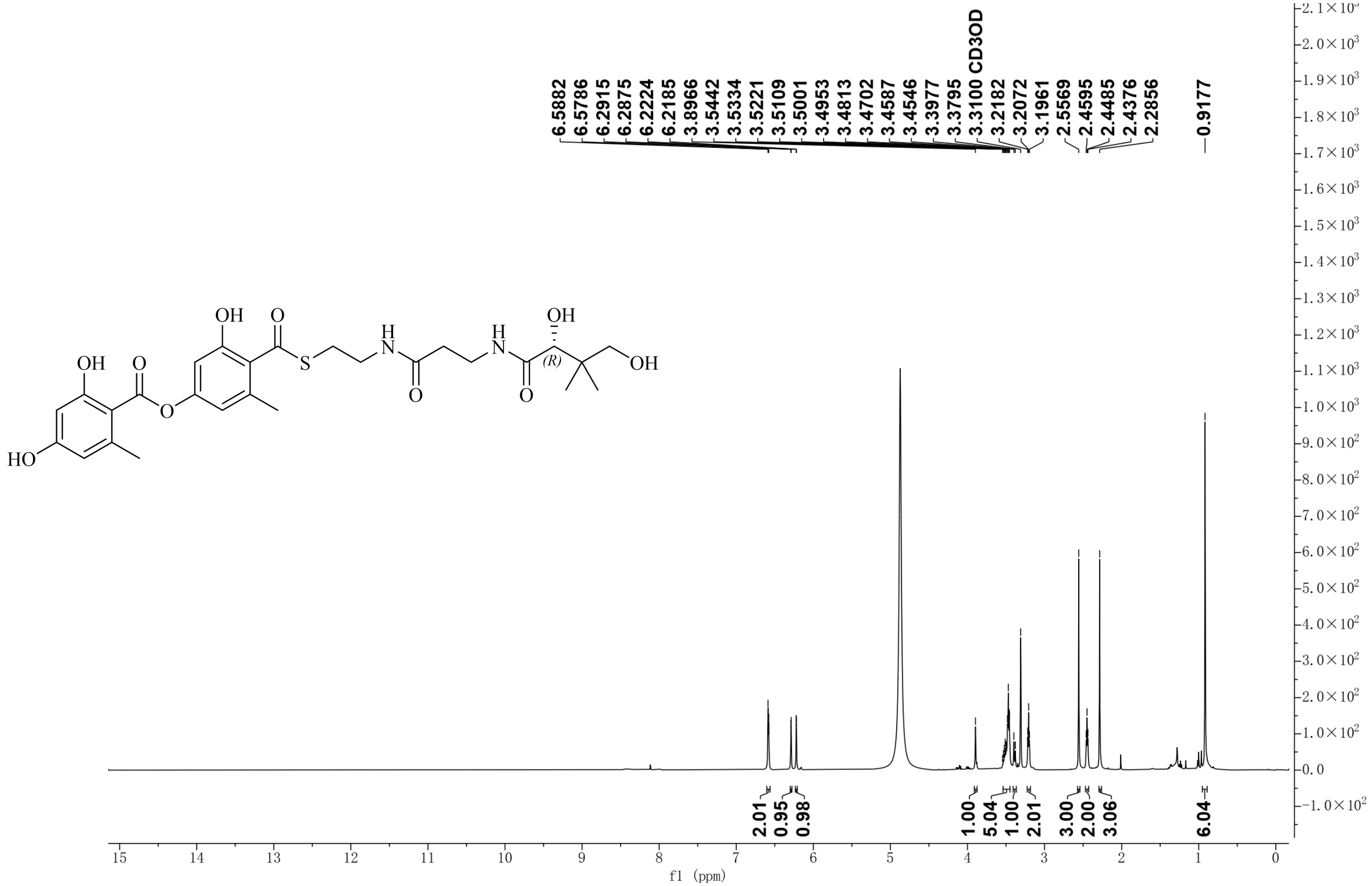

Figure S14.1. ^{1}H NMR spectrum of **9** in methanol-d_4.

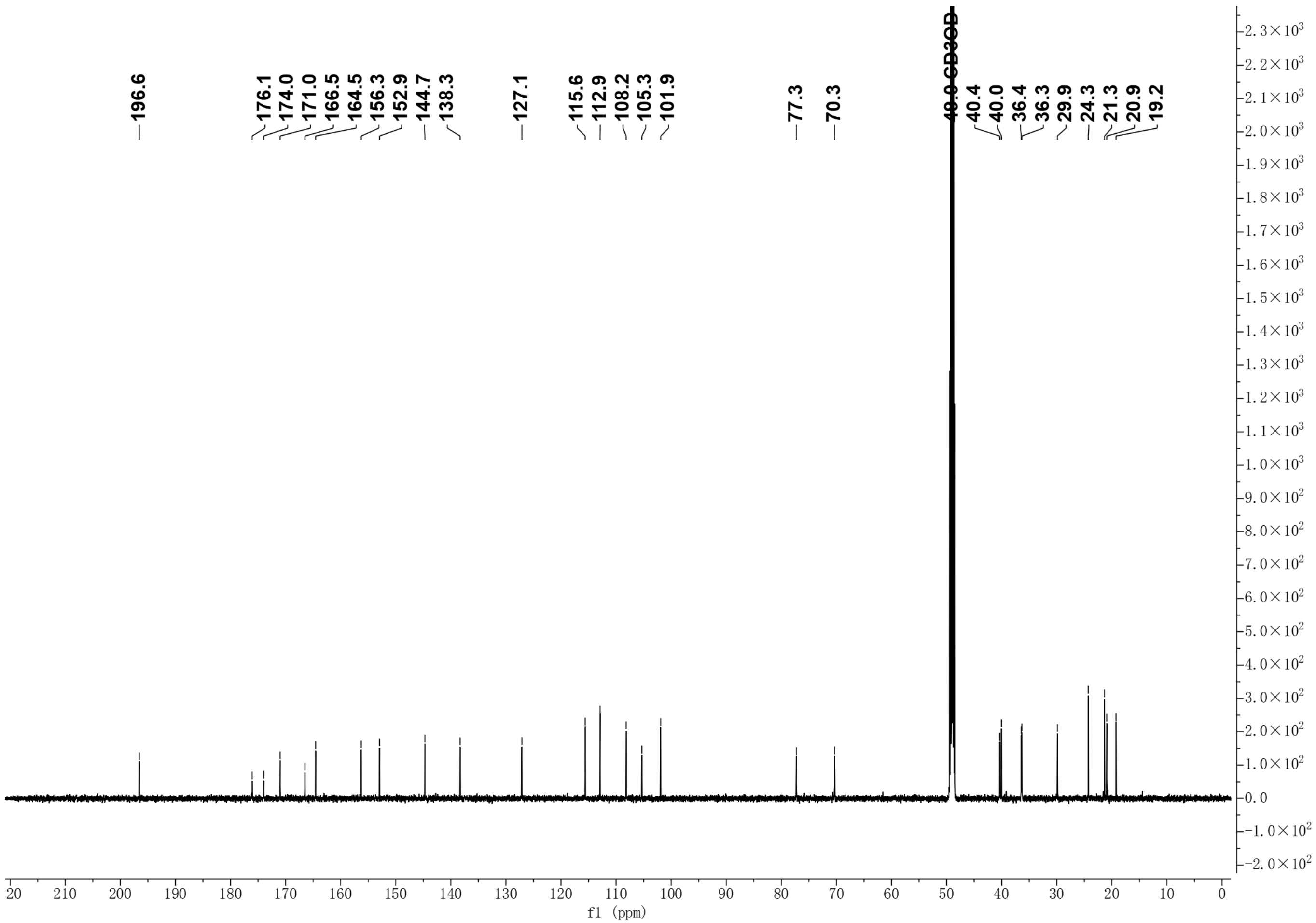

Figure S14.2. ^{13}C NMR spectrum of **9** in methanol-d_4.

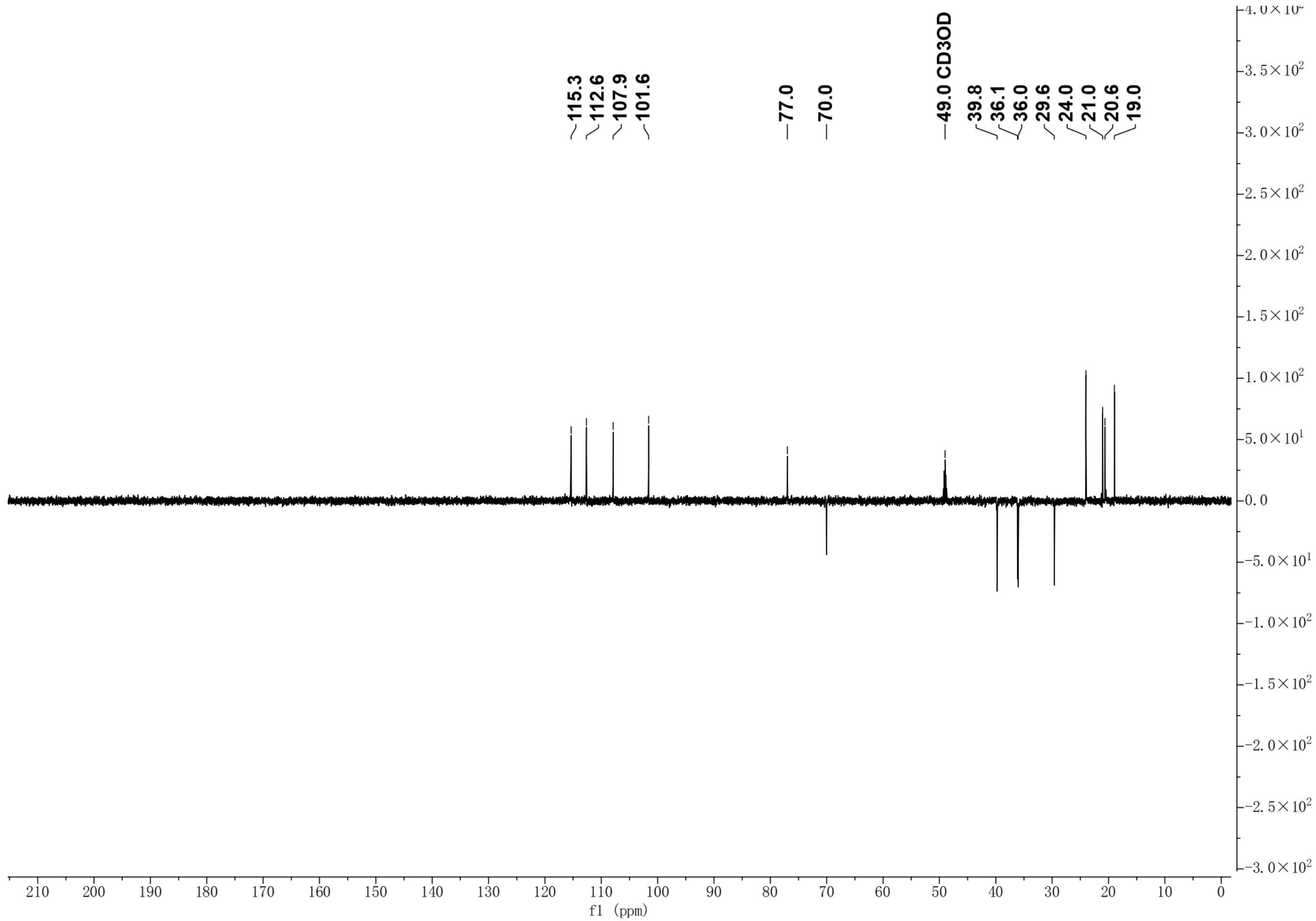

Figure S14.3. DEPT 135 spectrum of **9** in methanol-d_4.

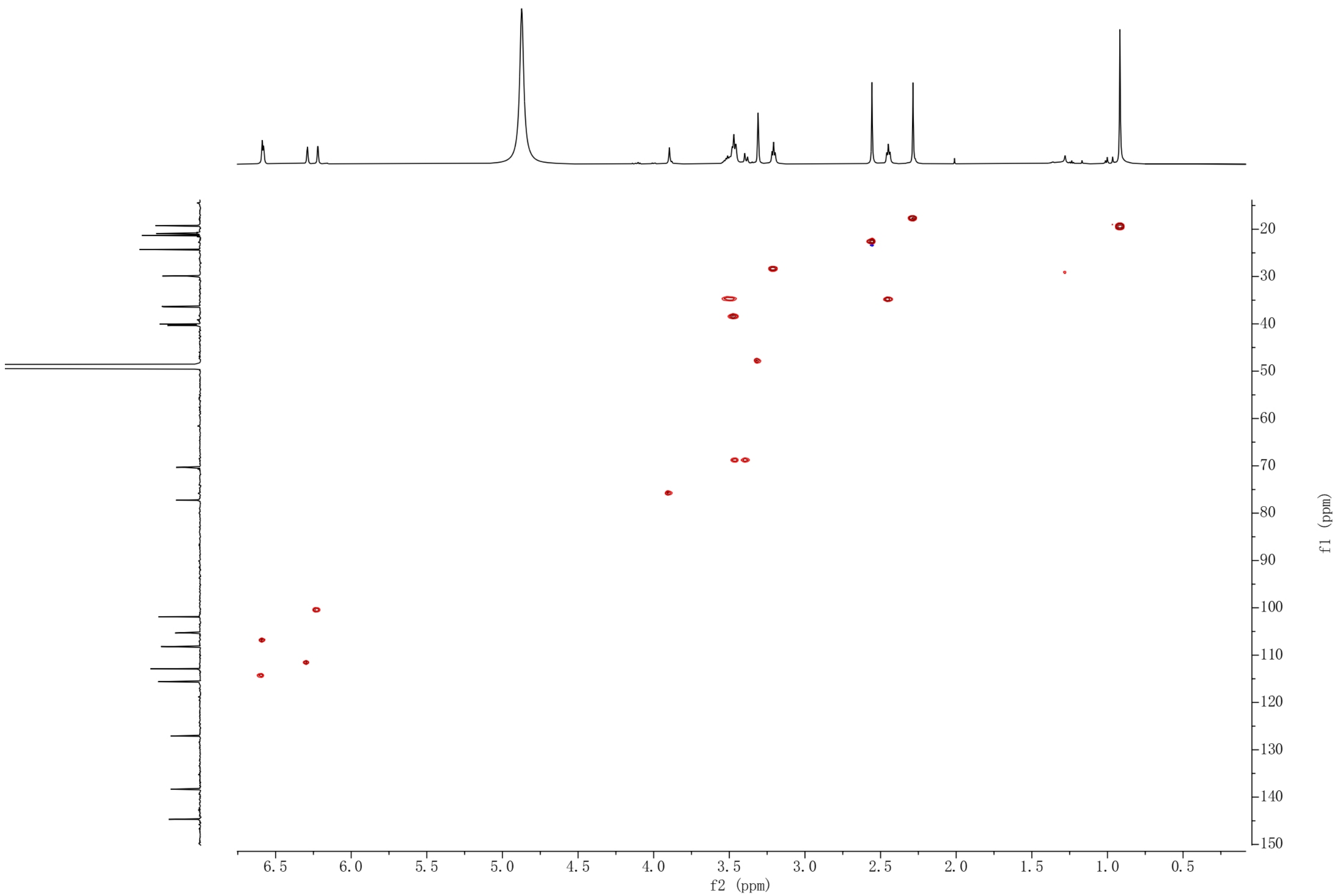

Figure S14.4. HSQC spectrum of **9** in methanol-d_4.

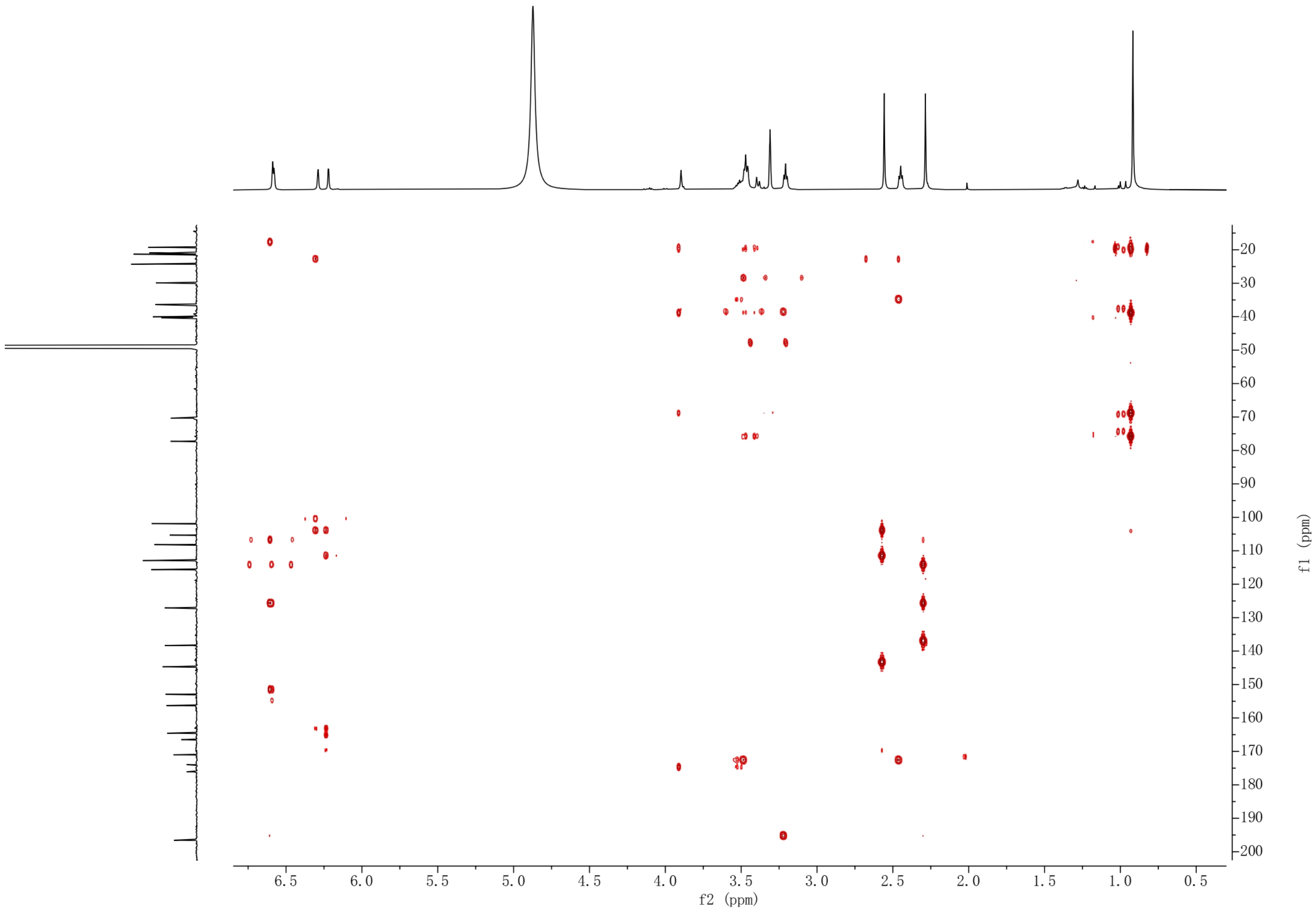

Figure S14.5. HMBC spectrum of **9** in methanol-d_4.

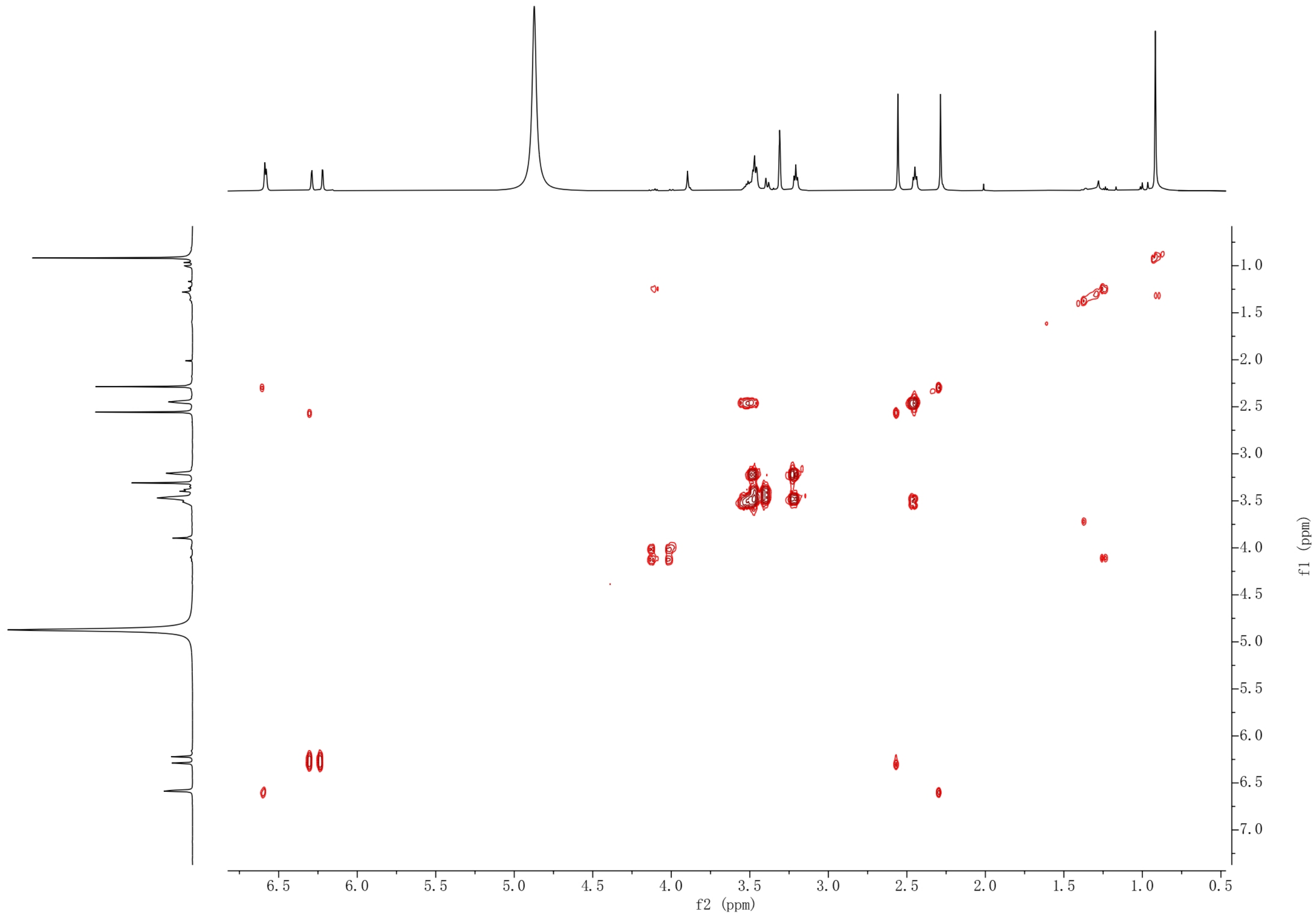

Figure S14.6. ^{1}H-^{1}H COSY spectrum of **9** in methanol-d_4.

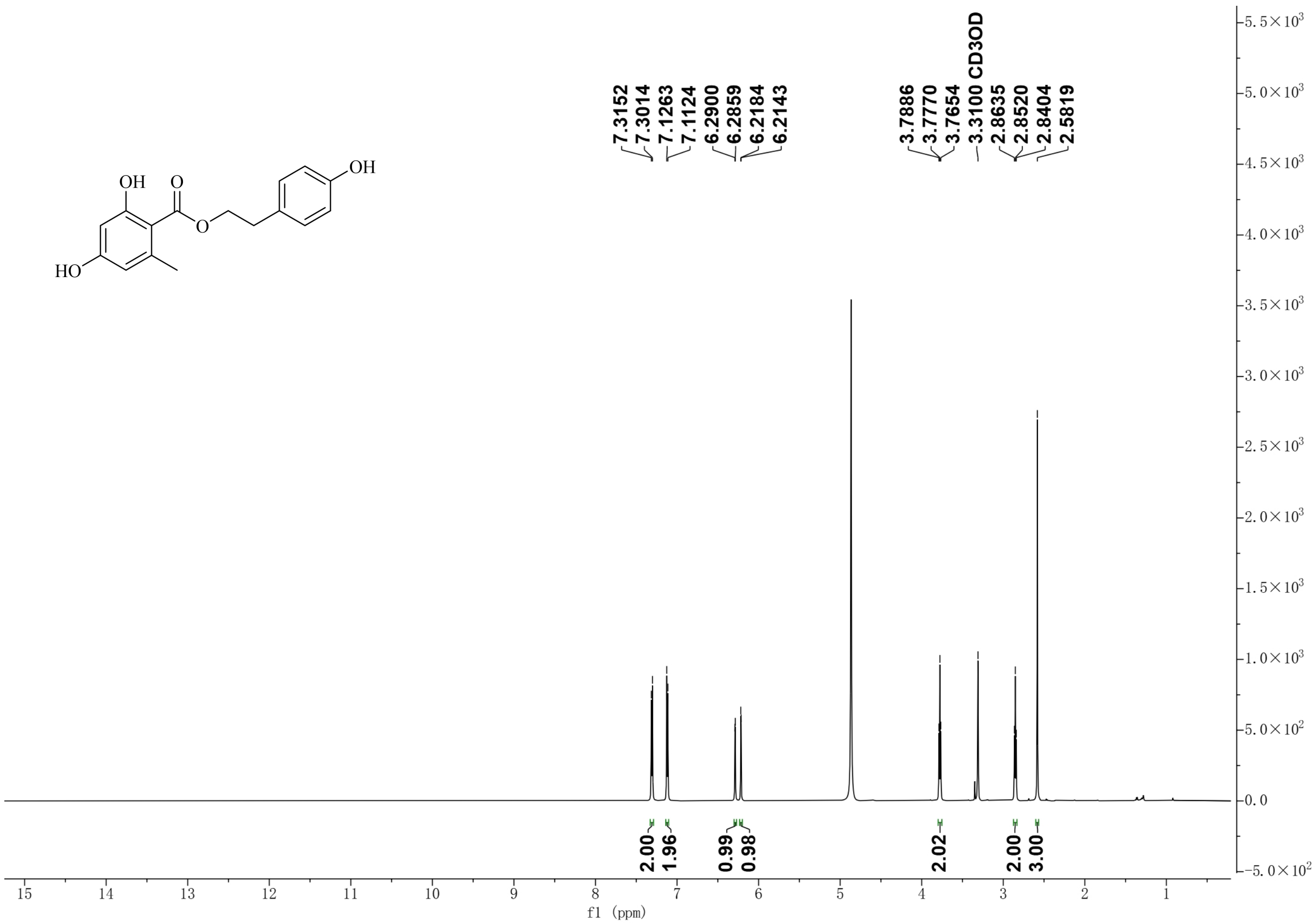

Figure S14.7. ^{1}H NMR spectrum of **10** in methanol-d_4.

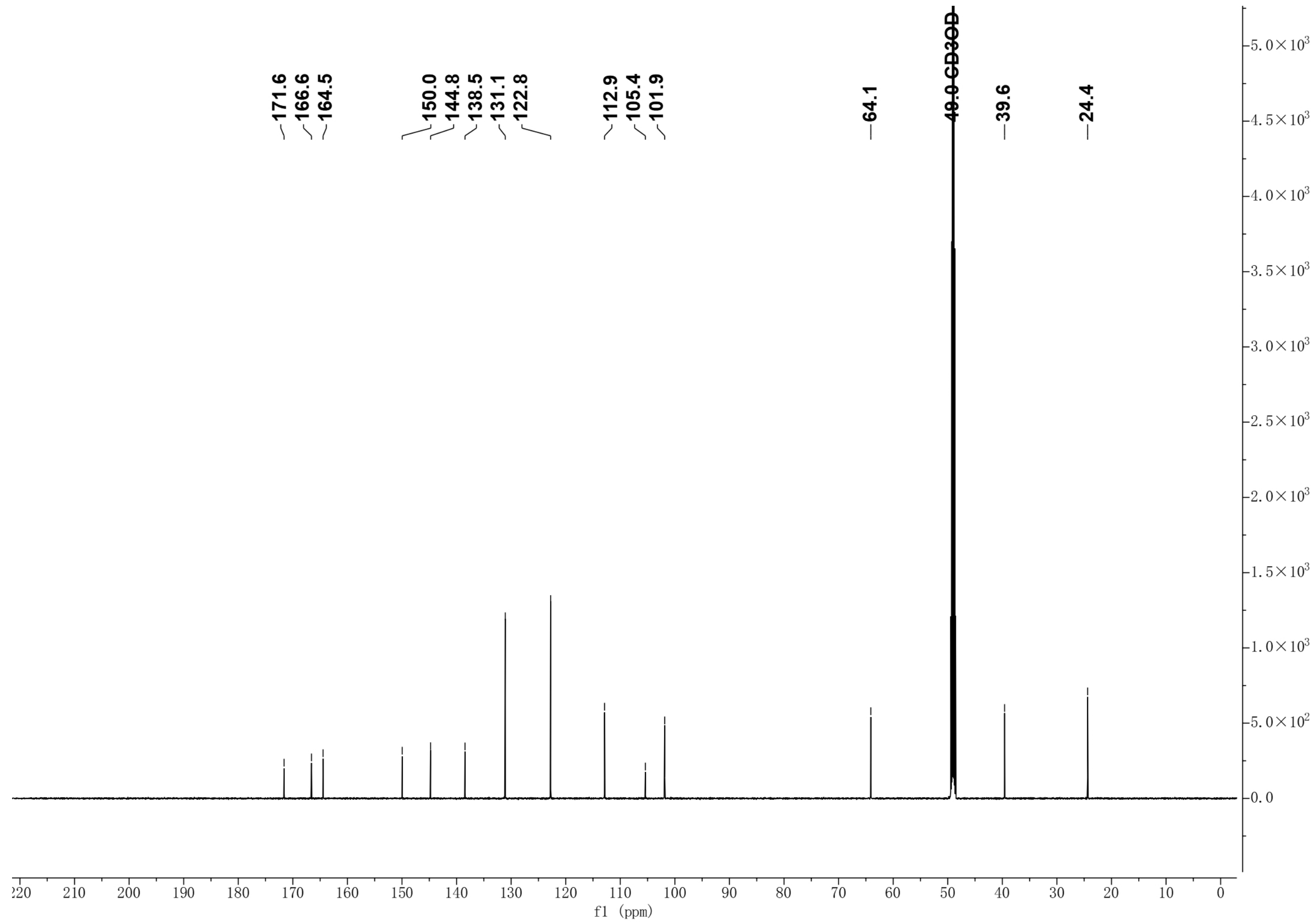

Figure S14.8. ^{13}C NMR spectrum of **10** in methanol-d_4.

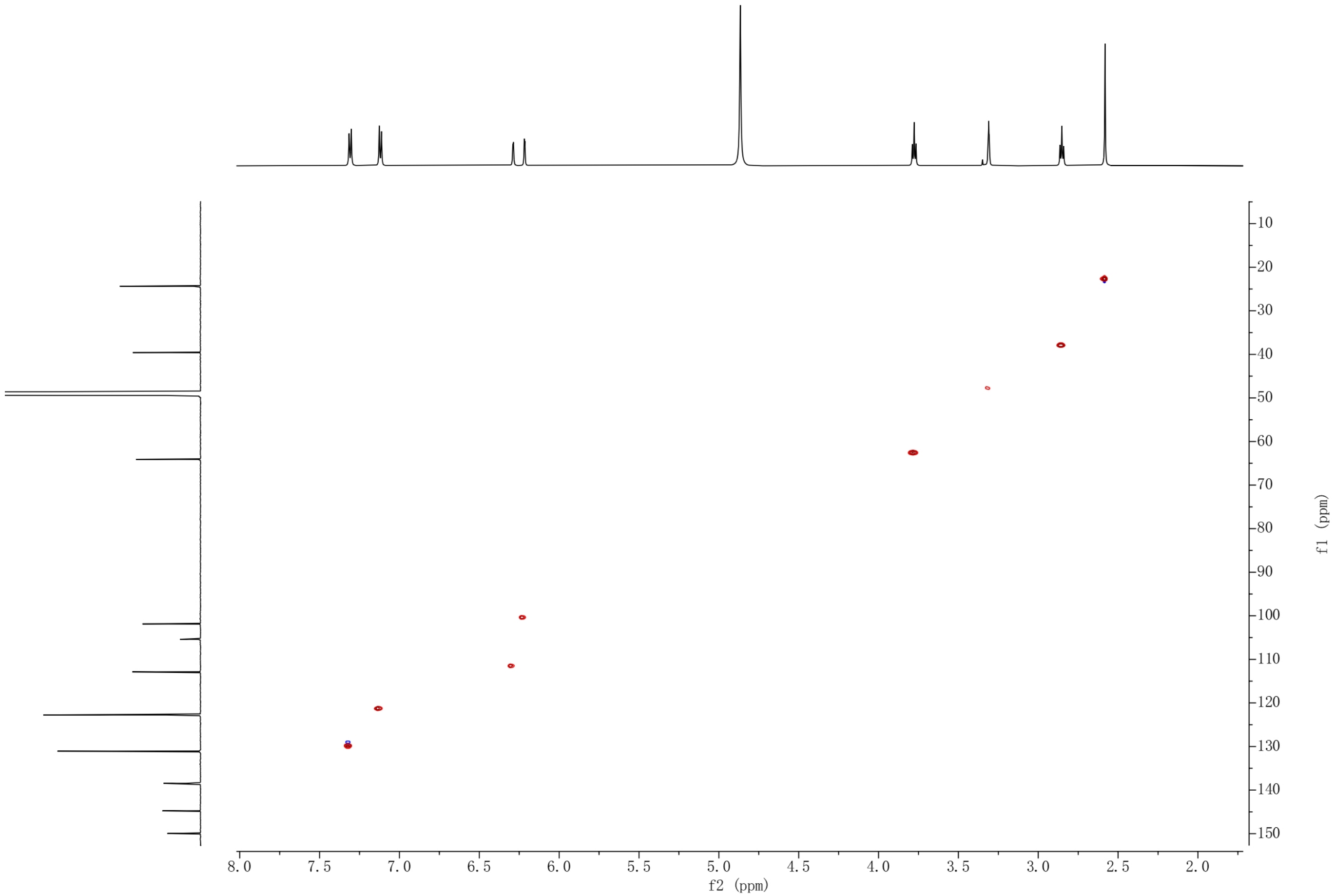

Figure S14.9. HSQC spectrum of **10** in methanol-d_4.

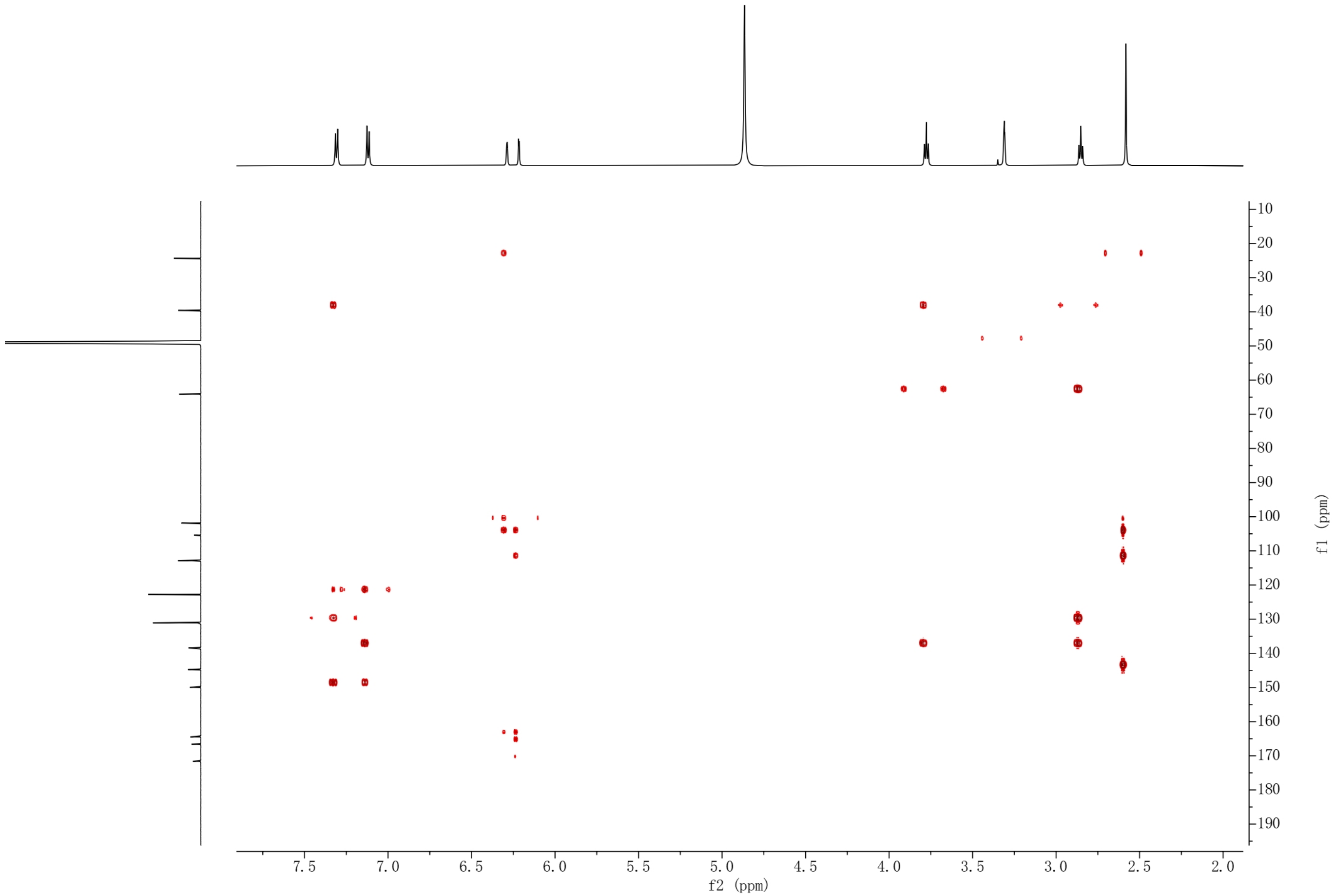

Figure S14.10. HMBC spectrum of **10** in methanol-d_4.

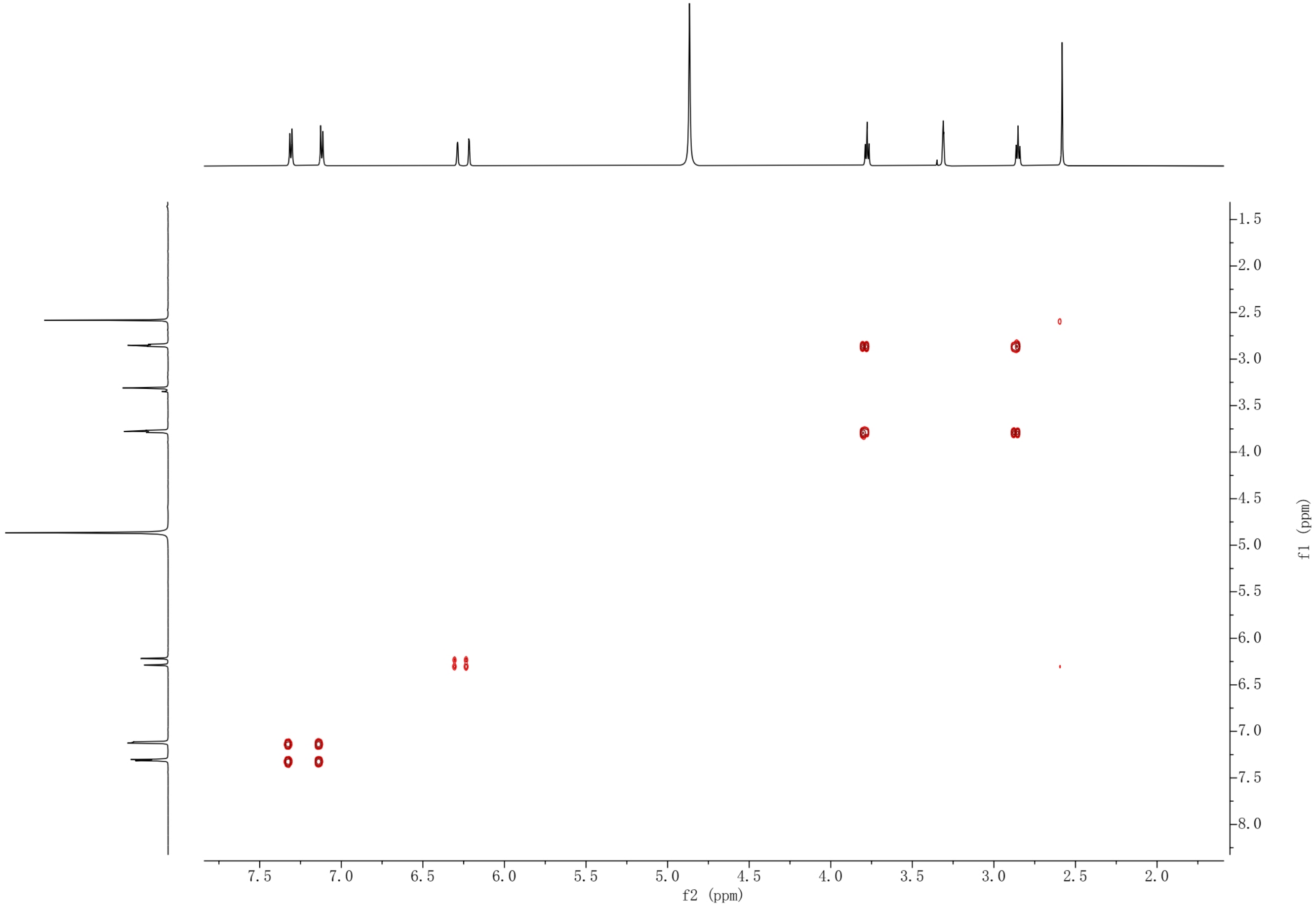

Figure S14.11. ^{1}H-^{1}H COSY spectrum of **10** in methanol-d_4.

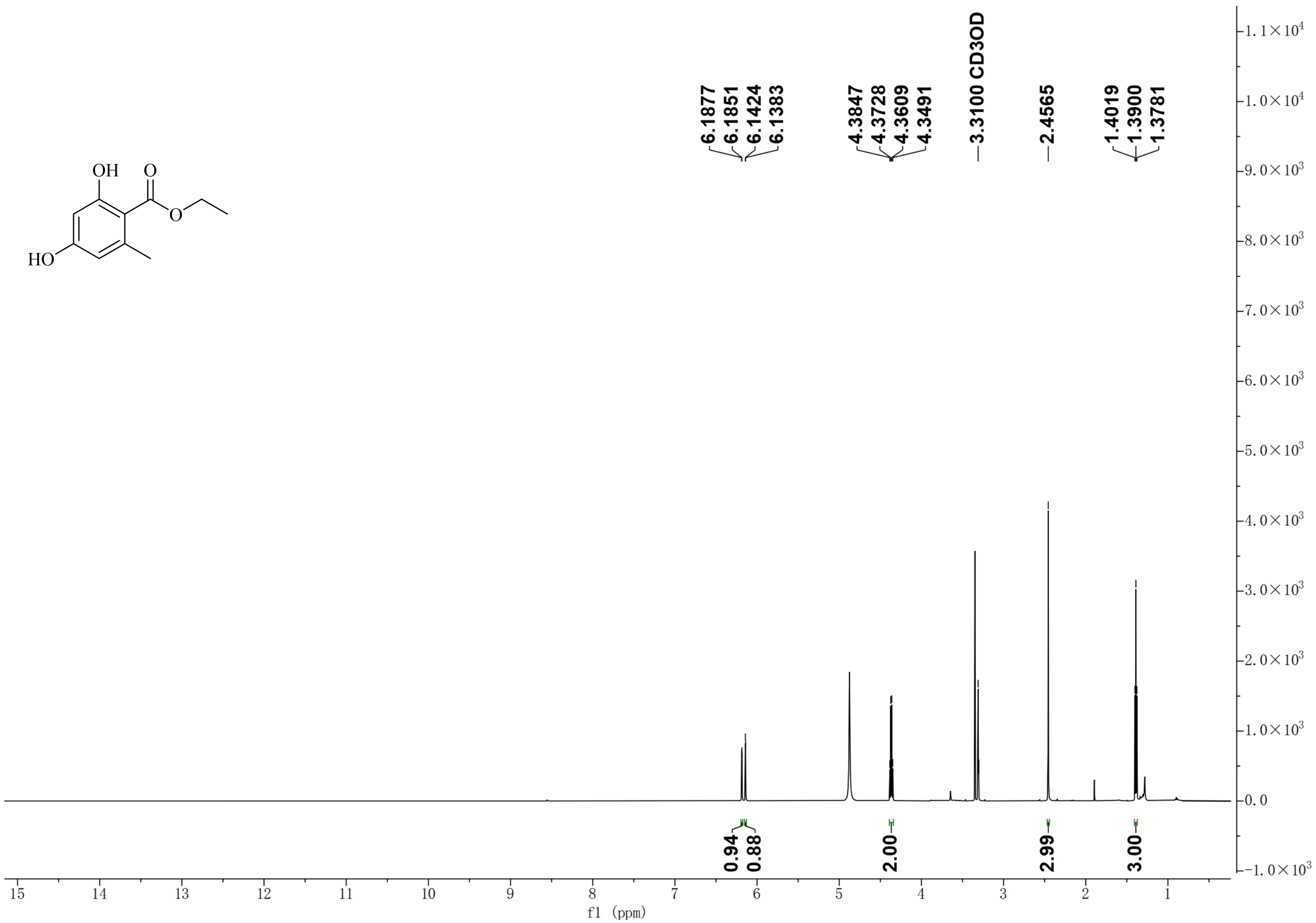

Figure S14.12. ^{1}H NMR spectrum of **11** in methanol-d_4.

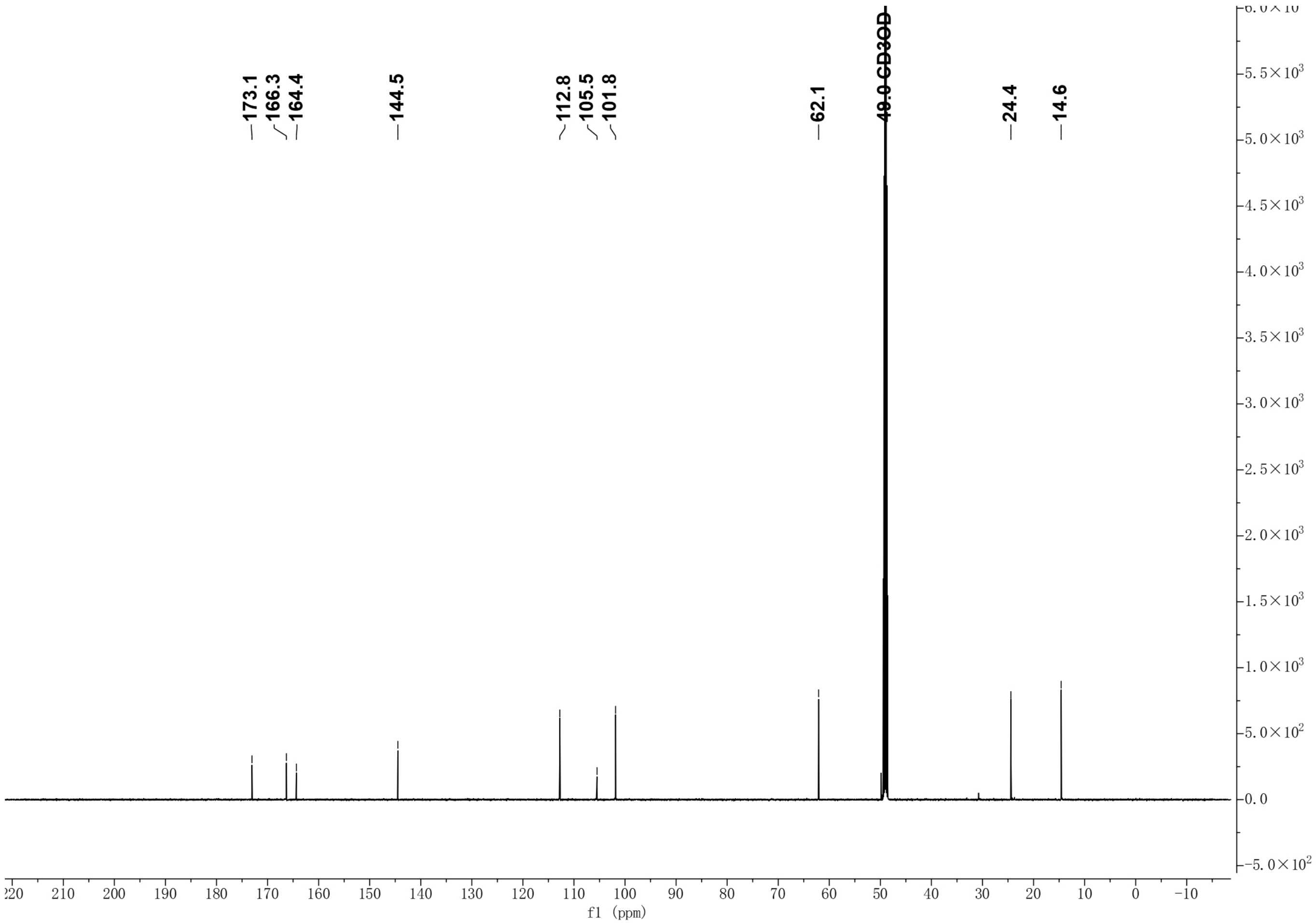

Figure S14.13. ^{13}C NMR spectrum of **11** in methanol-d_4.

REFERENCE

(1) Wang, X. J.; Wang, C.; Duan, L. X.; Zhang, L. W.; Liu, H.; Xu, Y. M.; Liu, Q. P.; Mao, T. L.; Zhang, W.; Chen, M.; Lin, M.; Gunatilaka, A. A. L.; Xu, Y. Q.; Molnár, I. Rational reprogramming *O*-methylation regioselectivity for combinatorial biosynthetic tailoring of benzenediol lactone scaffolds. *J. Am. Chem. Soc.* **2019**, *141*, 4355-4364. DOI: 10.1021/jacs.8b12967.

(2) Liu, Q. P.; Zhang, D.; Xu, Y.; Gao, S. B.; Gong, Y. F.; Cai, X. H.; Yao, M.; Yang, X. L. Cloning and functional characterization of the polyketide synthases based on genome mining of *Preussia isomera* XL-1326. *Front. Microbiol.* **2022**, *13*, 819086. DOI: 10.3389/fmicb.2022.819086.

(3) Liu, Q. P.; Zhang, D.; Gao, S. B.; Cai, X. H.; Yao, M.; Xu, Y.; Gong, Y. F.; Zheng, K.; Mao, Y. G.; Yang, L. Y.; Yang, D. F.; Molnár, I.; Yang, X. L. Didepside formation by the nonreducing polyketide synthase Preu6 of *Preussia isomera* requires interaction of starter acyl transferase and thioesterase domains. *Angew. Chem., Int. Ed.* **2023**, *62*, e202214379. DOI: 10.1002/anie.202214379.

(4) Zheng, Y. L. Mining and characterization of the related genes for the biosynthesis of spirocyclic polyketide minimoidione B. Master's Thesis, South-Central Minzu University, Wuhan, China, **2025**.

(5) Cai, X. H. Identification of the polyketide synthase Preu7 from *Preussia isomera*. Bachelor's Thesis, South-Central Minzu University, Wuhan, China, **2023**.

(6) Mao, Y. G. Mining of antimicrobial polyketides based on heterologous expression technology. Master's Thesis, South-Central Minzu University, Wuhan, China, **2023**.

(7) Xu, Y. Q.; Zhou, T.; Espinosa-Artiles, P.; Tang, Y.; Zhan, J. X.; Molnár, I. Insights into the biosynthesis of 12-membered resorcylic acid lactones from heterologous production in *Saccharomyces cerevisiae. ACS Chem. Biol.* **2017**, *12*, 216-223. DOI: 10.1021/cb500043g.

(8) Xu, Y. Q.; Espinosa-Artiles, P.; Schubert, V.; Xu, Y. M.; Zhang, W.; Lin, M.; Gunatilaka, A. A. L.; Süssmuth, R.; Molnár, I. Characterization of the biosynthetic genes for 10,11-Dehydrocurvularin, a heat shock response-modulating anticancer fungal polyketide from *Aspergillus terreus*. *Appl. Environ. Microbiol.* **2013**, *79*, 2038-2047. DOI: 10.1128/AEM.03334-12.

(9) Udwary, D. W.; Merski, M.; Townsend, C. A. A method for prediction of the locations of linker regions within large multifunctional proteins, and application to a type I polyketide synthase. *J. Mol. Biol.* **2002**, *323*, 585-598. DOI: 10.1016/S0022-2836(02)00972-5.

(10) Chen, L.; Wei, X. X.; Matsuda, Y. Depside bond formation by the starter-unit acyltransferase domain of a fungal polyketide synthase. *J. Am. Chem. Soc.* **2022**, *144*, 19225-19230. DOI: 10.1021/jacs.2c08585.

(11) Wei, Q.; Wang, Z. P.; Zhang, X.; Zou, Y. Diaryl ether formation by a versatile thioesterase domain. *J. Am. Chem. Soc.* **2022**, *144*, 9554-9558. DOI: 10.1021/jacs.2c02813.

(12) Liu, Q. P.; Gao, S. B.; Fang, J.; Gong, Y. F.; Zheng, Y. L.; Xu, Y.; Zhang, D.; Wei, J. Y.; Liao, L. X.; Yao, M.; Wang, W. J.; Han, X. L.; Chen, F. S.; Molnár, I.; Yang, X. L. Novel fungal diphenyl ether biosynthetic gene clusters encode a promiscuous oxidase for elevated antibacterial activities. *Chem. Sci.* **2024**, *15*, 14248-14253. DOI: 10.1039/d4sc01435a.

(13) Ma, S. M.; Li, J. W. H.; Choi, J. W.; Zhou, H.; Lee, K. K. M.; Moorthie, V. A.; Xie, X. K.; Kealey, J. T.; Da Silva, N. A.; Vederas, J. C.; Tang, Y. Complete reconstitution of a highly reducing iterative polyketide synthase. *Science* **2009**, *326*, 589-592. DOI: 10.1126/science.1175602.

(14) Gietz, R. D.; Schiestl, R. H. Quick and easy yeast transformation using the LiAc/SS carrier DNA/PEG method. *Nat. Protoc.* **2007**, *2*, 35-37. DOI: 10.1038/nprot.2007.14.

(15) Abramson, J.; Adler, J.; Dunger, J.; Evans, R.; Green, T.; Pritzel, A.; Ronneberger, O.; Willmore, L.; Ballard, A. J.; Bambrick, J.; Bodenstein, S. W.; Evans, D. A.; Hung, C.-C.; O'Neill, M.; Reiman, D.; Tunyasuvunakool, K.; Wu, Z.; Žemgulytė, A.; Arvaniti, E.; Beattie, C.; Bertolli, O.; Bridgland, A.; Cherepanov, A.; Congreve, M.; Cowen-Rivers, A. I.; Cowie, A.; Figurnov, M.; Fuchs, F. B.; Gladman, H.; Jain, R.; Khan, Y. A.; Low, C. M. R.; Perlin, K.; Potapenko, A.; Savy, P.; Singh, S.; Stecula, A.; Thillaisundaram, A.; Tong, C.; Yakneen, S.; Zhong, E. D.; Zielinski, M.; Žídek, A.; Bapst, V.; Kohli, P.; Jaderberg, M.; Hassabis, D.; Jumper, J. M. Accurate structure prediction of biomolecular interactions with AlphaFold 3. *Nature* **2024**, *630*, 493-500. DOI: 10.1038/s41586-024-07487-w.

(16) Zhang, C. X.; Shine, M.; Pyle, A. M.; Zhang, Y. US-align: universal structure alignments of proteins, nucleic acids, and macromolecular complexes. *Nat. Methods* **2022**, *19*, 1109-1115. DOI: 10.1038/s41592-022-01585-1.

(17) Pettersen, E. F.; Goddard, T. D.; Huang, C. C.; Meng, E. C.; Couch, G. S.; Croll, T. I.; Morris, J. H.; Ferrin, T. E. UCSF ChimeraX: Structure visualization for researchers, educators, and developers. *Protein Sci.* **2021**, *30*, 70-82. DOI: 10.1002/pro.3943.

(18) Wei, Q.; Wang, Z. P.; Zhang, X.; Zou, Y. Diaryl ether formation by a versatile thioesterase domain. *J. Am. Chem. Soc.* **2022**, *144*, 9554-9558. DOI: 10.1021/jacs.2c02813.

(19) Kong, D.; He, Q.; Lin, D. M.; Zhang, H.; Chen, L.; Fan, Y.; Tang, M. C.; Zou, Y. Serine hydrolase-catalyzed polyol lipids are necessary for rodlet layer formation on the cell wall of entomopathogenic fungi. *J. Am. Chem. Soc.* **2025**, *147*, 4701-4706. DOI: 10.1021/jacs.4c15577.

(20) Liu, S. Z.; Wang, W. J.; Liu, Q. P.; Yao, M.; Liao, L. X.; Gao, S. B.; Yu, Y.; Yang, X. L. Emerindanols A and B: two bipolyhydroindenol sesterterpenes with 5/6−6/5 coupled ring system discovered by genome mining. *Org. Lett.* **2024**, *26*, 4475-4479. DOI: 10.1021/acs.orglett.4c01272.

(21) Lu, H. M.; Tan, Y. H.; Zhang, Y. T.; Li, Z. C.; Chen, J. Y.; Gao, C. H.; Liu, Y. H.; Luo, X. W. Osteoclastogenesis inhibitory phenolic derivatives produced by the Beibu Gulf Coral-associated fungus *Acremonium sclerotigenum* GXIMD 02501. *Fitoterapia* **2022**, *159*, 105201. DOI: 10.1016/j.fitote.2022.105201.

(22) Wu, X.; Fang, L. Z.; Liu, F. L.; Pang, X. J.; Qin, H. L.; Zhao, T.; Xu, L. L.; Yang D. F.; Yang, X. L. New prenylxanthones, polyketide hemiterpenoid pigments from the endophytic fungus *Emericella* sp. XL029 and their anti-agricultural pathogenic fungal and antibacterial activities. *RSC Adv.* **2017**, *7*, 31115-31122. DOI: 10.1039/C7RA04762B

(23) Liao, L. X.; Huang, J. G.; Zhu, T.; Ran, S.; Liu, Q. P.;Wang, W. J.; Yang, X. L. HDAC Inhibitor SAHA triggers the production of previously undescribed sesquiterpenes and undergoes biotransformation by the fungus *Robillarda sessilis* from *Verbena officinalis* L. *Phytochemistry* **2025**, *233*, 114411. DOI: 10.1016/j.phytochem.2025.114411.
